# Footwear Heel Height and Gait Biomechanics in Healthy Young Women: A Within-Subject Analysis of Spatiotemporal Parameters, Propulsion, and Pelvic Kinematics

**DOI:** 10.3390/life16060977

**Published:** 2026-06-10

**Authors:** Alina-Daniela Totorean, Oana Cristina Radulescu, Alexandra-Magdalena Ioana, Laura Maghiar, Andreea Nita, Andreea-Adriana Neamțu, Elena Amaricai, Roxana Ramona Onofrei, Oana Suciu, Cristina Dumitrescu, Dan Iliescu, Florin Huț

**Affiliations:** 1 Department of Rehabilitation, Physical Medicine and Rheumatology, “Victor Babes” University of Medicine and Pharmacy, Eftimie Murgu Square, No. 2, 300041 Timisoara, Romania; totorean.alina@umft.ro (A.-D.T.); amaricai.elena@umft.ro (E.A.); onofrei.roxana@umft.ro (R.R.O.); oanasuciu78@umft.ro (O.S.); 2 Department of Orthopedics and Traumatology II, Compartment of Medical Rehabilitation, “Pius Brinzeu” Clinical County Emergency Hospital Timisoara, Liviu Rebreanu Boulevard, No. 156, 300723 Timisoara, Romania; 3 Doctoral School, “Victor Babes” University of Medicine and Pharmacy, Eftimie Murgu Square, No. 2, 300041 Timisoara, Romania; oana.radulescu@umft.ro (O.C.R.); alexandra.ioana@umft.ro (A.-M.I.); 4 Department of Anatomy and Embriology, “Victor Babes” University of Medicine and Pharmacy, Eftimie Murgu Square, No. 2, 300041 Timisoara, Romania; 5 Department of Psycho-Neurosciences and Rehabilitation, Faculty of Medicine and Pharmacy, University of Oradea, Universității Str., No. 1, 410087 Oradea, Romania; laura.maghiar@uoradea.ro; 6 Department of Dermatovenerology, Clinical County Emergency Hospital Bihor, 410169 Oradea, Romania; 7 Research Center for Assessment of Human Motion, Functionality and Disability, “Victor Babes” University of Medicine and Pharmacy, Eftimie Murgu Square, No. 2, 300041 Timisoara, Romania; 8 Department of Toxicology, “Victor Babes” University of Medicine and Pharmacy, Eftimie Murgu Square, No. 2, 300041 Timisoara, Romania; cristina.grosu@umft.ro; 9 Research Centre for Pharmaco-Toxicological Evaluation, “Victor Babes” University of Medicine and Pharmacy, Eftimie Murgu Square, No. 2, 300041 Timisoara, Romania; 10 Department of Pathology, Clinical County Emergency Hospital of Arad, Andrenyi Karoly Str., No. 2–4, 310037 Arad, Romania; 11 Department of Pathology, “Pius Brinzeu” Clinical County Emergency Hospital Timisoara, Liviu Rebreanu Boulevard, No. 156, 300723 Timisoara, Romania; 12 Department of Orthopedics and Traumatology I, Compartment of Medical Rehabilitation, “Pius Brinzeu” Clinical County Emergency Hospital Timisoara, Liviu Rebreanu Boulevard, No. 156, 300723 Timisoara, Romania; 13 Transdisciplinary Research Center in Medical Rehabilitation, Balneology and Rheumatology, “Victor Babes” University of Medicine and Pharmacy, 300041 Timisoara, Romania; 14 Department of Surgery I—Clinic of Surgical Semiotics & Thoracic Surgery, Center for Hepato-Biliary and Pancreatic Surgery, Faculty of Medicine, “Victor Babes” University of Medicine and Pharmacy, Eftimie Murgu Square, No. 2, 300041 Timisoara, Romania; dan.iliescu@umft.ro (D.I.); florin.hut@umft.ro (F.H.)

**Keywords:** high-heeled shoes, gait analysis, gait spatiotemporal parameters, propulsion index, pelvic kinematics, inertial measurement, footwear biomechanics, stride length

## Abstract

Background: High-heeled footwear is widely used by women, yet its systemic influence on spatiotemporal gait parameters, pelvic kinematics, and propulsion across a range of heel heights remains incompletely characterised. This study aimed to quantify gait changes across four footwear conditions and assess the contribution of anthropometric characteristics to observed gait variability. Methods: A within-subject repeated-measures study was conducted with 75 healthy young adult women (mean age 24.3 years, BMI 21.3 kg/m^2^) assessed barefoot, in ballerina flats, 8 cm heels, and 12 cm heels using the G-WALK inertial measurement system (BTS Bioengineering). Thirty gait parameters were analysed using the Friedman test with Bonferroni-corrected Wilcoxon post hoc comparisons (α_adj_ = 0.0083), Spearman rank correlations, multiple linear regression, and Kruskal–Wallis tertile analysis. Results: Footwear significantly affected 22 of 30 parameters. Walking speed was higher in all shod conditions than barefoot (up to +9.2%), driven entirely by stride elongation with cadence unchanged, indicating a general effect of footwear rather than heel elevation specifically. Stride length peaked at 8 cm heel (+8.9% vs. barefoot) and declined at 12 cm. Gait symmetry decreased progressively with heel height. Ballerina shoes produced a distinctively dynamic temporal profile—shortest stance duration, lowest double support, and highest single support time—significantly different from both barefoot and heeled conditions. The propulsion index increased height-dependently with heel height, rising 23.3% from barefoot (8.20) to 12 cm heel (10.11; *p* < 0.001). Pelvic obliquity symmetry was disrupted at 12 cm heel, while tilt symmetry was unaffected. Anthropometric analysis identified 110/600 significant Spearman correlations (23 surviving Benjamini–Hochberg FDR correction) and 29/120 significant regression models (14 surviving FDR); age, body weight, and shoe size were the most consistent predictors, most reliably in the barefoot condition. Conclusions: Heel height exerts condition-specific effects on gait biomechanics. Ballerina shoes produce a gait pattern distinct from both barefoot and heeled walking. Propulsion demand increases height dependently with heel elevation. Because participants walked in their own footwear, the observed effects reflect the combined characteristics of each shoe type rather than heel elevation in isolation. Anthropometric characteristics—particularly age, body weight, and shoe size—are modestly associated with footwear–gait responses and may inform future biomechanical research, although clinical application requires confirmation in standardised-footwear studies and clinical populations.

## 1. Introduction

The human foot represents one of the most complex anatomical structures within the musculoskeletal system. Its unique architecture—including the medial and lateral longitudinal arches and the transverse arch—provides both support for body weight in upright posture and an elastic mechanism for efficient locomotion, redistributing ground reaction forces during gait and reducing energetic cost (arches of the foot) [1].

Foot skeletal and soft-tissue interactions allow it not only to bear mechanical loads but also to serve as a rich source of somatosensory input that contributes to postural control. Sensory feedback from plantar cutaneous receptors and intrinsic foot muscles integrates with vestibular and visual systems to detect shifts in body orientation and trigger corrective postural responses (foot sole sensory role) [2].

Compared with other weight-bearing joints such as the knee or hip, the joints and bones of the foot may be subjected to additional mechanical stresses. These arise not only from body weight and ground contact but also from modifications induced by footwear design, which can alter the foot–ground interaction and consequently change gait mechanics and posture [3]. Shoes with elevated heels impose a plantarflexed foot posture, limiting natural ankle range of motion and shifting the centre of pressure, which has been linked to decreased postural stability and altered balance strategies in both static and dynamic conditions (high heel effects on postural stability) [4].

Variations in heel height and shoe design can significantly affect gait and stability: elevated heels reduce stride length and walking speed, alter foot clearance and joint kinematics, and may increase the risk of imbalance or fall, especially when habitual gait patterns are disrupted (effects of high-heeled shoes on gait parameters) [4]. This biomechanical perturbation presumably arises from changes in somatosensory input, altered ankle strategy reliance, and adjustments in motor control to maintain equilibrium [3,4].

Maintaining balance requires the integration of plantar somatosensory input to regulate the centre of mass within the base of support, and alterations at the foot can impair both postural control and walking performance [5,6].

Intrinsic foot-muscle activity is closely associated with the control of postural sway, and disruption of this plantar afferent feedback can alter gait patterns and muscle activation [7].

Footwear represents one of the main external factors influencing both foot statics and dynamics. In particular, high-heeled shoes have been shown to significantly affect postural control, increasing instability and altering plantar pressure distribution [4,8]. These changes can lead to compensatory biomechanical adaptations and increased stress on the musculoskeletal system.

The type of footwear, particularly in adult women, becomes an important factor influencing both posture and gait. While during childhood and adolescence footwear is generally uniform and low-heeled, thereafter is an increased use of shoes with varying heel heights. This transition contributes to measurable changes in biomechanics, gait parameters, and postural control [9,10].

Although heel height is known to alter individual gait parameters, few studies have examined its effects across the full temporal structure of the gait cycle, propulsion, and pelvic kinematics within a single within-subject design, or have systematically tested whether individual anthropometric characteristics are associated with the magnitude of these responses. The primary aim of this study was therefore to quantify changes in spatiotemporal gait parameters, propulsion, and pelvic kinematics across four footwear conditions (barefoot, ballerina flats, 8 cm heels, and 12 cm heels) in healthy young women, with walking speed, stride length, and the propulsion index as the principal outcomes. A secondary, exploratory aim was to assess the extent to which individual anthropometric characteristics (age, height, body weight, BMI, and shoe size) are associated with these gait responses. We hypothesised that (i) increasing heel height would progressively reduce gait symmetry and increase the propulsion index, whereas walking speed and stride length would change non-linearly across conditions, and (ii) anthropometric characteristics would show only modest associations with the footwear-related gait changes.

## 2. Materials and Methods

### 2.1. Study Design and Participants

This study was designed as a within-subject repeated-measures (experimental) study and included 75 healthy young adult female volunteers. All participants were clinically healthy and had no known medical conditions that could influence gait parameters. Particular attention was given to excluding individuals according to the criteria detailed below.

### 2.2. Inclusion Criteria

Participants were eligible for inclusion if they met the following criteria:

- Female, aged ≥ 18 years.
- Habitual, comfortable use of—and ability to provide—their own well-fitting closed-toe footwear across the tested heel-height range (ballerina flats 1 cm, 8 cm heels, 12 cm heels), such that participants were accustomed to walking in heeled shoes.

### 2.3. Exclusion Criteria

The exclusion criteria were:

- History of lower-limb trauma, or known rheumatic, musculoskeletal, neurological, or systemic condition affecting gait.
- Current or previous pregnancy.
- Recent (<1 year) major (>10%) change (loss or gain) in body weight.

### 2.4. Footwear Conditions and Gait Assessment

Each participant was assessed under four walking conditions. Barefoot walking (1) was used as the reference condition. Subsequently, gait parameters were recorded while participants walked wearing their own closed-toe shoes with different heel heights: 1 cm ballet flats (2), 8 cm heels (3), and 12 cm heels (4). Participants used their own footwear in order to reproduce habitual walking conditions as closely as possible. Shoes with thin soles and comparable general characteristics were selected, with heel height being the main variable of interest.

### 2.5. Gait Analysis System

Gait assessment was performed using the G-WALK system (BTS Bioengineering S.p.A., Garbagnate Milanese, Italy), which includes an inertial sensor, the G-SENSOR, used for data acquisition. The validated G-WALK protocol [11] was applied in order to obtain information regarding spatiotemporal gait parameters, as well as general kinematic parameters and pelvic kinematics during walking.

For the “Walk” protocol, the sensor was positioned at the level of the lumbosacral transition, below the line connecting the two posterior superior iliac spine dimples, commonly referred to as the “dimples of Venus”. This anatomical landmark corresponds approximately to the S1–S2 vertebral level. The sensor was fixed according to the manufacturer’s recommendations in order to ensure stable data acquisition throughout the walking trial [11].

### 2.6. Testing Procedure

Before the start of the test, each participant received standardised instructions regarding the correct execution of the walking task. The test began with the participant standing in an upright position for a few seconds to allow stabilisation and sensor calibration. Participants were then instructed to walk at their natural, self-selected walking speed along a straight path.

The walking distance was greater than 7 m, allowing the recording of at least five complete gait cycles [11]. At the end of the straight path, participants performed a 180° turn with an approximate turning radius of 1 m, after which they continued walking at the same natural speed until returning to the initial starting position.

To improve the reproducibility and accuracy of the test, the starting and finishing points, the walking path, and the turning point were clearly marked on the floor before data collection. The same testing protocol was applied for the barefoot control and all footwear conditions.

### 2.7. Data Collected

A total of 30 gait parameters were recorded for each participant and each footwear condition using the G-WALK inertial measurement system. These encompassed spatiotemporal parameters—including cadence (steps/min), walking speed (m/s), stride duration (s), stride length (m), stride length normalised to participant height (%), stance duration (%), swing duration (%), double support duration (%), and single support duration (%)—as well as propulsion index and pelvic kinematic symmetry indices for tilt, obliquity, and rotation. Where applicable, parameters were recorded separately for the left and right sides.

The propulsion index is a dimensionless index derived by the G-WALK system from antero-posterior trunk acceleration during push-off; it serves as a proxy for propulsive demand and does not represent a directly measured kinetic force.

For each participant and condition, three walking trials were performed and the resulting values were averaged to yield a single representative measurement per parameter per condition. For bilateral parameters (left and right), the mean of the two sides was computed to obtain a bilateral average for primary analyses. The bilateral average was used in all statistical comparisons unless the left and right sides were analysed separately for descriptive purposes.

### 2.8. Data Analysis

#### 2.8.1. Descriptive Statistics

Descriptive statistics were calculated for all 30 gait parameters across the four footwear conditions. Results are presented as mean and 95% confidence interval (95% CI [lower–upper]), calculated using Student’s t-distribution (df = 74). Baseline anthropometric characteristics of the study sample are reported using the same format.

#### 2.8.2. Primary Gait Analysis—Effect of Footwear Condition

To assess the overall effect of footwear condition on each gait parameter, the Friedman repeated-measures non-parametric test was applied (df = 3, N = 75). The Friedman test was chosen because the within-subject repeated-measures design does not permit the assumption of normality for the difference scores across all parameters, and it is the appropriate non-parametric analogue of the one-way repeated-measures analysis of variance. The significance threshold was set at *p* < 0.05 for the overall condition effect.

For parameters yielding a significant Friedman result, post hoc pairwise comparisons were performed using the Wilcoxon signed-rank test. This test is appropriate for paired non-parametric data, as it accounts for the within-subject structure by comparing each participant’s own measurements between pairs of conditions. With four footwear conditions, the number of unique pairwise combinations is:

k = 6 comparisons: barefoot vs. ballerina, barefoot vs. 8 cm heel, barefoot vs. 12 cm heel, ballerina vs. 8 cm heel, ballerina vs. 12 cm heel, and 8 cm heel vs. 12 cm heel.

To control the family-wise Type I error rate across these six comparisons per parameter, Bonferroni correction was applied, yielding an adjusted significance threshold of α_adj_ = 0.05/6 = 0.0083. Post hoc results are reported as Bonferroni-corrected *p*-values (p_adj_) and are considered significant when p_adj_ < 0.05. For each post hoc comparison, the matched-pairs rank-biserial correlation (r) was additionally computed as a measure of effect size (|r| ≈ 0.1, 0.3, and 0.5 denoting small, medium, and large effects, respectively); effect sizes for all pairwise comparisons are reported in Supplementary Table S2.

#### 2.8.3. Anthropometric Influence on Gait

The influence of five anthropometric variables—age, height, body weight, body mass index (BMI), and European shoe size—on gait parameters was assessed using three complementary approaches.

First, Spearman rank correlation coefficients were computed between each anthropometric variable and each gait parameter within each footwear condition, yielding a total of 600 correlation tests (30 parameters × 5 variables × 4 conditions). Spearman’s ρ was selected over Pearson’s r because it does not assume bivariate normality and is robust to the presence of outliers. Because this analysis was exploratory and involved a large number of tests, the Benjamini–Hochberg false discovery rate (FDR) procedure was applied within each analysis family to control the expected proportion of false positives; associations were considered robust when they survived FDR correction at q < 0.05. Uncorrected results (*p* < 0.05) are additionally reported to characterise the overall pattern of associations, which is regarded as hypothesis-generating rather than confirmatory.

Second, multiple linear regression (MLR) models were fitted for each gait parameter within each footwear condition (120 models total: 30 parameters × 4 conditions), using the five anthropometric variables (age, height, weight, BMI, and shoe size) as simultaneous predictors. Standardised regression coefficients (β) are reported to allow comparison of predictor magnitudes across models with different units of measurement. Model significance was assessed by the overall F-test (*p* < 0.05), with Benjamini–Hochberg FDR correction applied across the 120 models (q < 0.05); the proportion of variance explained is reported as R^2^. Multicollinearity among the five predictors was assessed using the variance inflation factor (VIF). Because body weight, height, and BMI are mathematically interdependent (BMI = weight/height^2^), their VIFs were high (weight = 42.7, BMI = 37.6, height = 10.5; all > 10), whereas age (1.1) and shoe size (1.8) were low. The individual standardised coefficients for weight, BMI, and height are therefore unstable and are interpreted with caution; primary inference relies on overall model fit (R^2^, F-test) and on the low-VIF predictors.

Third, Kruskal–Wallis (KW) tests were performed to compare gait parameters across tertile groups of age, height, and body weight within each footwear condition (360 tests: 30 parameters × 3 variables × 4 conditions). Participants were stratified into low, mid, and high tertiles for each continuous anthropometric variable. BMI and shoe size were not included in the tertile analysis: BMI is a composite of height and weight and would introduce collinearity with variables already stratified; shoe size is a discrete ordinal variable with only five unique values (EU 36–40), for which tertile stratification yields unequal and potentially insufficient group sizes. The influence of BMI and shoe size on gait was therefore characterised exclusively through Spearman rank correlation and multiple linear regression. The Kruskal–Wallis H statistic (df = 2) is reported for each comparison (*p* < 0.05), with Benjamini–Hochberg FDR correction applied across the 360 tests (q < 0.05), together with the direction of the effect across tertiles.

All statistical analyses were performed using Microsoft Excel (version 16.83, Microsoft Corporation, Redmond, WA, USA), GraphPad Prism (version 10.1.2, GraphPad Software LLC, San Diego, CA, USA), and RStudio (version 2025.05.0+496, Posit Software, PBC, Boston, MA, USA). The significance threshold was set at *p* < 0.05 throughout, unless otherwise specified.

### 2.9. Ethics and Consent

Participation in the study was entirely voluntary. All participants were informed about the purpose and procedures of the study prior to enrolment, and written informed consent was obtained from each participant before data collection commenced. Participants were free to withdraw from the study at any point without consequence.

The study was conducted in accordance with the Declaration of Helsinki and applicable data protection regulations. Prior to data collection, the study protocol was submitted to the Ethics Committee of the Clinical County Emergency Hospital “Pius Brinzeu” Timișoara, Romania, which issued the ethics approval (Decision No. 240, dated 20 April 2021), in accordance with national and European applicable guidelines. The study involved no physical interventions beyond a standardised walking test using a validated non-invasive inertial measurement system, and posed no foreseeable physical, psychological, or legal risks to participants.

All data were collected and stored in accordance with applicable data protection regulations (GDPR). Participant data were handled confidentially and anonymised by the clinician before analysis; no identifying information is reported in this manuscript, neither is it in the supporting raw data (provided as Supplementary Materials Table S1).

### 2.10. Use of Generative Artificial Intelligence (GenAI)

During the preparation of this manuscript, the authors used ChatGPT (OpenAI, GPT-5.5 version) for image generation and language refinement assistance. The authors have reviewed and edited the output and take full responsibility for the content of this publication.

## 3. Results

### 3.1. Participant Description

**Figure 1:**
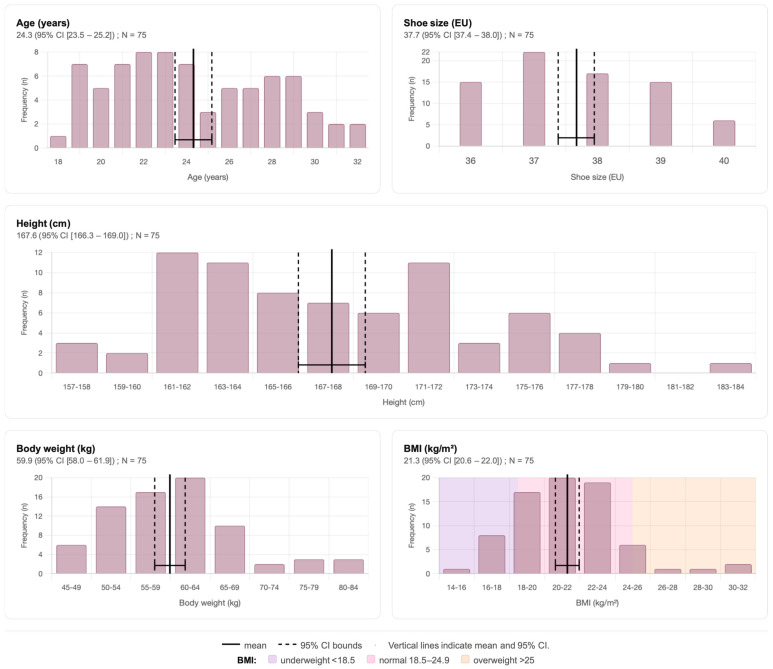
Distribution of baseline anthropometric characteristics among the 75 study participants. Frequency histograms are shown for age (years), height (cm), body weight (kg), BMI (kg/m^2^), and European shoe size. The dashed vertical line indicates the mean and the horizontal bracket indicates the 95% confidence interval.

A total of 75 healthy young adult female volunteers were recruited and included in the final analysis. Mean age was 24.3 (95% CI [23.5–25.2]) years, with individual values ranging from 18 to 32 years. Mean height was 167.6 (95% CI [166.3–169.0]) cm, mean body weight was 59.9 (95% CI [58.0–61.9]) kg, and mean BMI was 21.3 (95% CI [20.6–22.0]) kg/m^2^. According to the World Health Organization classification, 57 participants (76.0%) were of normal weight, 12 (16.0%) were underweight, and 6 (8.0%) were overweight (n = 4) or obese (n = 2); individual BMI values ranged from 15.8 to 30.5 kg/m^2^. The sample was therefore concentrated in the normal and lower BMI range. Mean European shoe size was 37.7 (95% CI [37.4–38.0]), with size 37 being the most frequent (n = 22). Detailed baseline characteristics are presented in Table 1, and the distribution of anthropometric variables across the sample is illustrated in Figure 1.

### 3.2. Primary Gait Analysis

**Figure 2:**
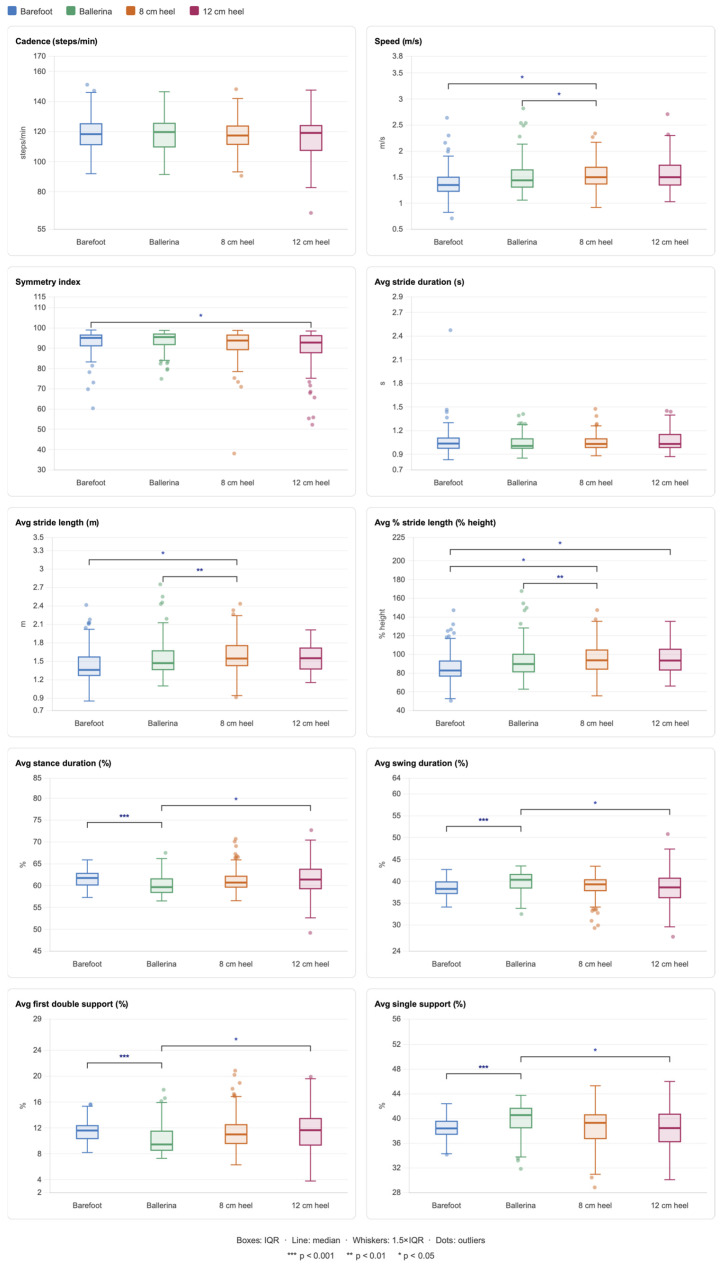
Box plots of primary gait parameters across footwear conditions (N = 75). Boxes represent the interquartile range (IQR); horizontal line = median; whiskers extend to 1.5 × IQR; dots = outliers. Horizontal brackets indicate significant post hoc pairs (Bonferroni-corrected). *** *p* < 0.001; ** *p* < 0.01; * *p* < 0.05.

Of the 30 gait parameters evaluated, 22 showed a statistically significant overall effect of footwear condition (Friedman repeated-measures test, *p* < 0.05). Descriptive statistics and Friedman test results for all primary parameters are presented in Table 2; significant Bonferroni-corrected post hoc pairs (p_adj_ < 0.05) are detailed in Table 3 and Figure 2. Values throughout are reported as mean (95% CI [lower–upper]).

#### 3.2.1. Walking Speed and Cadence

Walking speed differed significantly across footwear conditions (χ^2^ = 23.76, *p* < 0.001). Mean speed increased progressively from 1.41 m/s (95% CI [1.34–1.48]) barefoot to 1.52 m/s (95% CI [1.44–1.60]) in ballerina shoes, reaching 1.54 m/s (95% CI [1.48–1.60]) at 8 cm heel and remaining stable at 1.54 m/s (95% CI [1.46–1.61]) at 12 cm heel. Significant pairwise differences were identified between barefoot and 8 cm heel (p_adj_ = 0.011) and between ballerina and 8 cm heel (p_adj_ = 0.037; Table 3). Cadence did not differ significantly across conditions (*p* > 0.05), with means ranging narrowly from 117.4 to 118.6 steps/min, indicating that participants maintained a stable stepping rate and adapted primarily through spatial rather than temporal mechanisms.

#### 3.2.2. Overall Symmetry Index

The overall symmetry index showed a significant condition effect (χ^2^ = 8.34, *p* = 0.040), with values declining progressively across conditions: 93.5 (95% CI [92.3–94.6]) for ballerina, 92.9 (95% CI [91.3–94.4]) barefoot, 91.2 (95% CI [89.3–93.2]) at 8 cm heel, and 89.5 (95% CI [87.1–91.9]) at 12 cm heel. A significant pairwise difference was observed between barefoot and the 12 cm heel condition (p_adj_ = 0.025), indicating that extreme heel elevation introduces a measurable reduction in bilateral gait symmetry; because no established clinical reference thresholds exist for this symmetry index, the biomechanical significance of a change of this magnitude remains to be determined.

#### 3.2.3. Stride Duration

Average stride duration did not differ significantly across conditions (χ^2^ = 0.86, *p* = 0.835), with mean values consistently between 1.04 and 1.07 s across all footwear. Together with the absence of cadence differences, this confirms that the temporal structure of the gait cycle was preserved across footwear conditions and that adaptations occurred primarily in the spatial domain.

#### 3.2.4. Stride Length

Stride length was significantly affected by footwear condition for all measures (all *p* < 0.001; Table 2). Mean average stride length increased from 1.46 m (95% CI [1.39–1.52]) barefoot to 1.56 m (95% CI [1.48–1.63]) in ballerina shoes, peaking at 1.59 m (95% CI [1.52–1.65]) at 8 cm heel before declining slightly to 1.55 m (95% CI [1.50–1.61]) at 12 cm heel. Significant pairwise differences were identified between barefoot and 8 cm heel (p_adj_ = 0.039) and between ballerina and 8 cm heel (p_adj_ = 0.006).

When expressed as a percentage of participant height, the pattern was consistent (all *p* < 0.001), with average normalised stride length increasing from 87.0% barefoot to 95.1% at 8 cm heel—an increase of approximately eight percentage points. Significant differences were found between barefoot and both 8 cm heel (p_adj_ = 0.019) and 12 cm heel (p_adj_ = 0.012), and between ballerina and 8 cm heel (p_adj_ = 0.002), confirming that stride elongation was not attributable to stature differences between participants.

#### 3.2.5. Stance and Swing Phase

Average stance duration showed a significant overall effect (χ^2^ = 21.32, *p* < 0.001). The ballerina condition produced the shortest stance duration (60.1%, 95% CI [59.6–60.6%]) compared to barefoot—61.5% (95% CI [61.1–61.9%]), p_adj_ < 0.001; stance then increased from ballerina to 12 cm heel—61.7%(95% CI [60.8–62.5%], p_adj_ = 0.024. Barefoot and 8 cm heel did not differ significantly (p_adj_ > 0.05). Average swing duration mirrored these changes in a complementary fashion (p_adj_ < 0.001 for barefoot vs. ballerina; p_adj_ = 0.024 for ballerina vs. 12 cm heel). These findings indicate that flat ballerina shoes show shorter ground contact and a proportionally longer swing phase, a pattern that reverses at higher heel heights.

#### 3.2.6. Double and Single Support

Average first double support duration was significantly affected by footwear condition (χ^2^ = 17.06, *p* < 0.001). The ballerina condition produced the lowest values (10.2%, 95% CI [9.7–10.7%]), significantly less than both barefoot (11.6%, 95% CI [11.2–12.0%]; p_adj_ < 0.001) and 12 cm heel (11.7%, 95% CI [11.0–12.4%]; p_adj_ = 0.026). The 8 cm heel condition (11.5%) did not differ significantly from barefoot, indicating that the reduction in bilateral weight-bearing time was specific to the ballerina condition. Average single support was correspondingly higher in the ballerina condition (39.9%, 95% CI [39.4–40.4%]) compared to both barefoot (38.5%; p_adj_ < 0.001) and 12 cm heel (38.4%; p_adj_ = 0.017).

### 3.3. Secondary Gait Analysis

**Figure 3:**
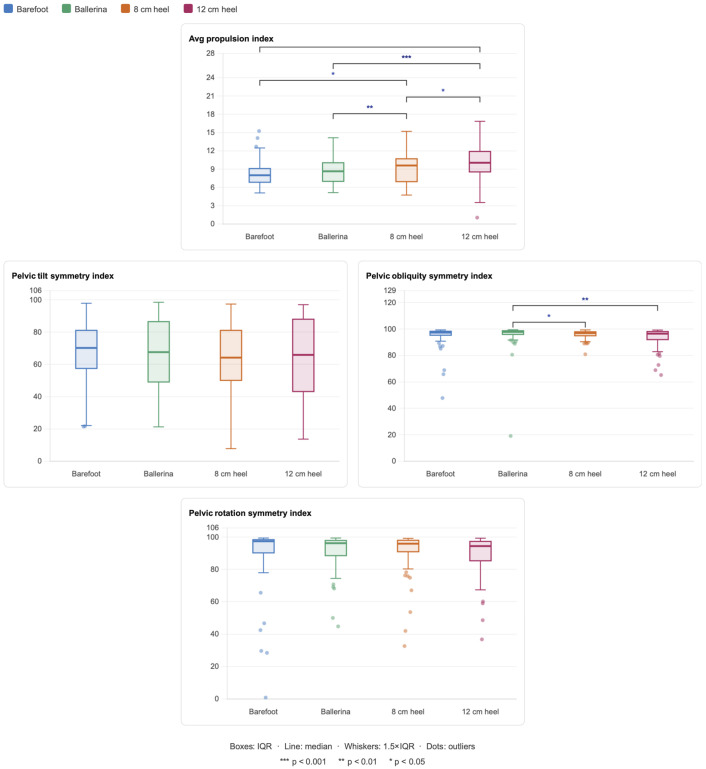
Box plots of secondary gait parameters across footwear conditions (N = 75). Boxes represent the IQR; horizontal line = median; whiskers = 1.5 × IQR; dots = outliers. Horizontal brackets indicate significant post hoc pairs (Bonferroni-corrected). Pelvic tilt and pelvic rotation symmetry indices showed a significant overall Friedman effect but no individual pair survived Bonferroni correction; no brackets are shown for these parameters. *** *p* < 0.001; ** *p* < 0.01; * *p* < 0.05.

Secondary parameters—propulsion index and pelvic kinematic symmetry indices—are presented in Table 4, with significant post hoc comparisons in Table 5 and Figure 3.

#### 3.3.1. Propulsion Index

The propulsion index showed a significant height-dependent increase with heel height across all measures (all *p* < 0.001; Table 4). Mean average propulsion index increased progressively from 8.20 (95% CI [7.76–8.65]) barefoot to 8.65 (95% CI [8.15–9.14]) in ballerina shoes, 9.23 (95% CI [8.69–9.78]) at 8 cm heel, and 10.11 (95% CI [9.46–10.76]) at 12 cm heel, representing a total increase of 23.3% from the barefoot to the 12 cm heel condition. Every comparison involving the 12 cm heel condition was significant (vs. barefoot: p_adj_ < 0.001; vs. ballerina: p_adj_ < 0.001; vs. 8 cm heel: p_adj_ = 0.011), as was the comparison between 8 cm heel and ballerina (p_adj_ = 0.007). Barefoot and ballerina conditions did not differ significantly (p_adj_ > 0.05). These findings are consistent with progressive ankle plantarflexion imposed by increasing heel height and a correspondingly greater estimated propulsive demand to maintain forward progression.

#### 3.3.2. Pelvic Kinematic Symmetry Indices

Pelvic tilt symmetry did not differ significantly across conditions (χ^2^ = 2.78, *p* = 0.426), with mean values between 63.1 and 66.9 across conditions, indicating that sagittal pelvic motion remained essentially unchanged regardless of footwear.

Pelvic obliquity symmetry showed a significant overall effect (χ^2^ = 18.11, *p* < 0.001). Values were stable across barefoot (95.2, 95% CI [93.4–97.0]), ballerina (95.9, 95% CI [93.7–98.1]), and 8 cm heel (95.9, 95% CI [95.2–96.6]), declining notably at 12 cm heel (93.7, 95% CI [92.1–95.3]). Significant pairwise differences were found between ballerina and 8 cm heel (p_adj_ = 0.025) and between ballerina and 12 cm heel (p_adj_ = 0.007), indicating that frontal-plane pelvic symmetry is disrupted primarily at extreme heel heights.

Pelvic rotation symmetry showed a significant overall Friedman effect (χ^2^ = 13.44, *p* = 0.004), with mean values ranging from 89.6 (12 cm heel) to 91.7 (ballerina). However, no individual post hoc pair survived Bonferroni correction, suggesting that the transverse-plane effect is distributed across conditions rather than localised to a specific footwear transition. Collectively, these findings indicate that high heel elevation disrupts pelvic symmetry predominantly in the frontal plane, with the sagittal plane remaining unaffected and the transverse plane showing a diffuse overall effect.

### 3.4. Influence of Anthropometric Characteristics on Gait Parameters

**Figure 4:**
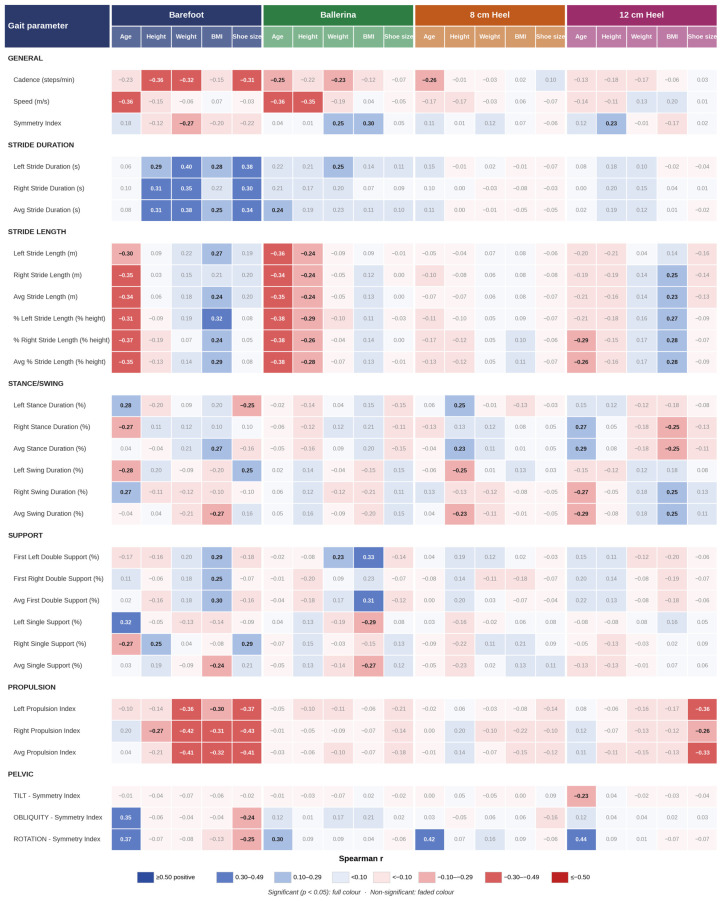
Spearman rank correlation heatmap between anthropometric variables and gait parameters across footwear conditions (N = 75). Each cell displays the Spearman r coefficient. Significant correlations (*p* < 0.05) are shown in full colour; non-significant values are shown in faded colour. Blue = positive correlation; red = negative correlation; intensity reflects |r|. Gait parameters are grouped by category.

**Figure 5:**
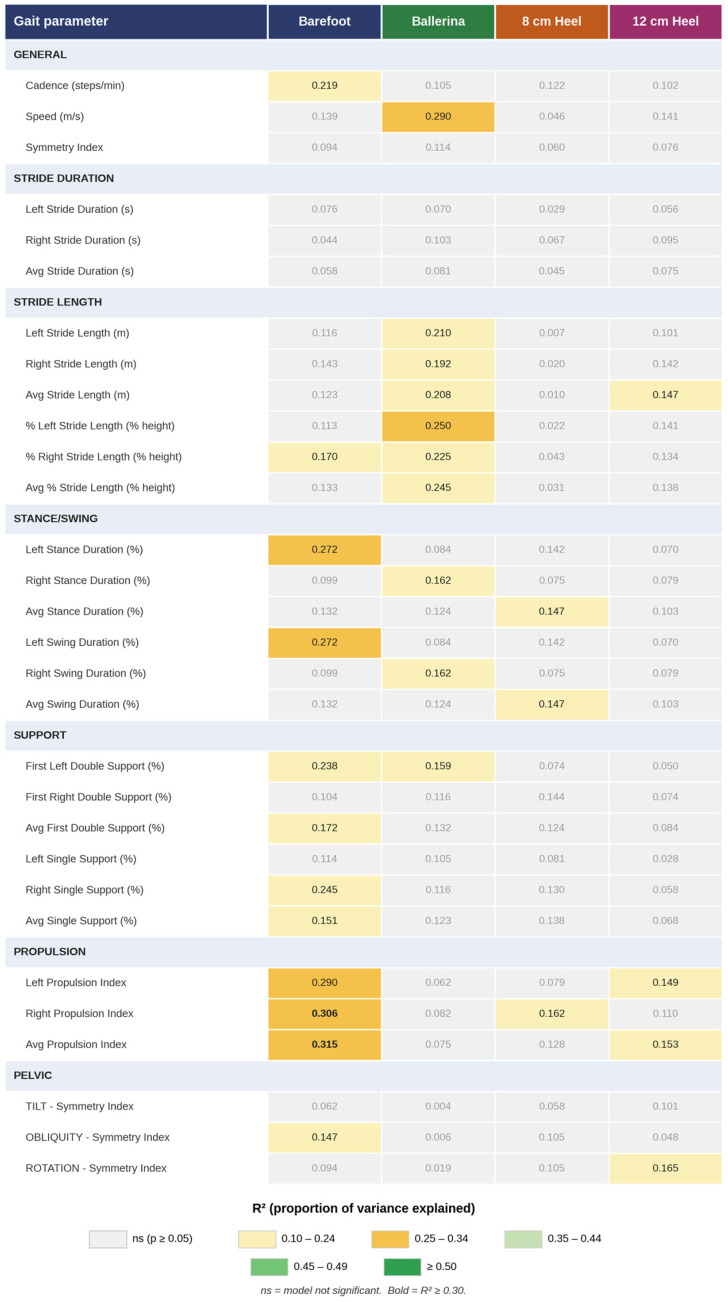
Multiple linear regression R^2^ heatmap showing the proportion of gait parameter variance explained by the anthropometric predictor set (age, height, body weight, BMI, shoe size) within each footwear condition (N = 75). Grey cells indicate non-significant models (*p* ≥ 0.05). Colour intensity reflects explanatory power from low (yellow, R^2^ 0.10–0.24) to very strong (dark green, R^2^ ≥ 0.50).

**Figure 6:**
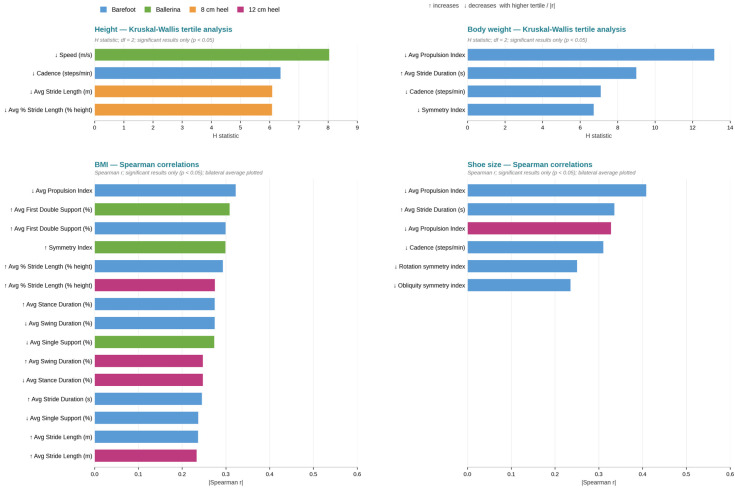
Significant Kruskal–Wallis tertile effects of anthropometric variables on gait parameters. Top row: H statistic for height and body weight tertile groups (df = 2; significant results only). Bottom row: Absolute Spearman r for BMI and shoe size (see Section 2.8.3 for rationale for method selection). Bar colours indicate footwear condition. ↑ increases, ↓ decreases with higher tertile or |r|. Bilateral average plotted where left and right measures are available.

The influence of five anthropometric variables—age, height, body weight, BMI, and European shoe size—on gait parameters was assessed across all four footwear conditions using three complementary analytical approaches: Spearman rank correlation (Figure 4), multiple linear regression (MLR) (Figure 5), and Kruskal–Wallis (KW) tests on tertiles of age, height, and body weight (Figure 6). Of 600 Spearman correlations computed, 110 (18.3%) were significant at *p* < 0.05—more than the ~30 expected under the null hypothesis—and 23 survived Benjamini–Hochberg FDR correction (q < 0.05). Of 120 regression models, 29 (24.2%) were significant and 14 survived FDR correction (vs. ~6 expected by chance). Of 360 Kruskal–Wallis tests, 100 (27.8%) were significant and 48 survived FDR correction (vs. ~18 expected by chance). Because the number of significant results exceeded chance expectation across all three approaches, the overall pattern is unlikely to be attributable to Type I error; the following subsections report the most clinically relevant associations, all of which survived FDR correction.

#### 3.4.1. Spearman Rank Correlations

Height. Height was correlated with gait timing primarily in the barefoot condition. It showed a positive correlation with stride duration barefoot (avg r = +0.306, *p* = 0.008) and a negative correlation with cadence barefoot (r = −0.356, *p* = 0.002)—the latter the only height association to survive FDR correction—consistent with the expected inverse relationship between stature and stepping rate. A negative correlation with walking speed was also present in the ballerina condition (r = −0.349, *p* = 0.002). The strong positive stride duration correlations were not reproduced in the shod conditions. A total of 18 of 120 height–gait pairs were significant (1 surviving FDR correction).

Body weight. Body weight demonstrated its strongest associations in the barefoot condition, where it was negatively correlated with propulsion index (avg r = −0.414; right r = −0.419; both *p* < 0.001) and positively correlated with stride duration (left r = +0.403; avg r = +0.379; both *p* < 0.001); these barefoot associations survived FDR correction and suggest that heavier participants generate proportionally lower estimated propulsion. The propulsion association was attenuated rather than amplified in the shod conditions, where it did not reach significance. Weight was significant in 12 of 120 weight–gait pairs (6 surviving FDR correction).

BMI. BMI showed 30 significant correlations across 120 pairs, but none survived FDR correction, indicating that its associations with gait were weak and not robust to multiplicity adjustment. The largest uncorrected associations were a positive correlation with first double support in the ballerina condition (r = +0.334, *p* = 0.003) and a negative correlation with propulsion index barefoot (r = −0.323, *p* = 0.005). BMI was therefore not a reliable independent predictor of gait in this sample.

Shoe size. Shoe size yielded 15 significant correlations (5 surviving FDR correction), predominantly with propulsion index and stride duration. The strongest associations were negative correlations with propulsion index—strongest barefoot (right r = −0.428; avg r = −0.409; both *p* < 0.001) and also present at the 12 cm heel (left r = −0.355, *p* = 0.002)—together with a positive correlation with stride duration barefoot (left r = +0.377, *p* < 0.001), reflecting that participants with larger feet tended to have longer gait cycles and lower relative propulsion. Because foot size correlates with stature, this pattern likely overlaps in part with the height effect.

Age. Age showed 35 significant correlations (11 surviving FDR correction). The most consistent pattern was a positive correlation with the pelvic rotation symmetry index, which survived FDR correction barefoot (r = +0.375, *p* < 0.001) and at the 8 cm (r = +0.423, *p* < 0.001) and 12 cm heel (r = +0.439, *p* < 0.001) conditions, with a weaker association in the ballerina condition (r = +0.299, *p* = 0.009), indicating that older participants in this sample displayed more symmetric pelvic rotation. Conversely, age was negatively correlated with stride length and normalised stride length in the ballerina condition (r = −0.38 to −0.37), suggesting that older participants took shorter steps relative to body height in flat footwear. Age was not significantly associated with propulsion index or most temporal parameters.

#### 3.4.2. Multiple Linear Regression

Of the 120 MLR models tested, 29 were statistically significant at *p* < 0.05, of which 14 survived FDR correction (q < 0.05); all models discussed below were among the FDR-surviving set. The highest explained variance was observed for the propulsion index in the barefoot condition: R^2^ = 0.315 for average propulsion (*p* < 0.001), with comparable values for right (R^2^ = 0.306) and left (R^2^ = 0.290) propulsion. Within the barefoot average propulsion model, body weight and BMI were the dominant predictors with opposing signs (β weight = +1.42, *p* = 0.033; β BMI = −1.64, *p* = 0.009), alongside height (β = −0.78, *p* = 0.018) and shoe size (β = −0.32, *p* = 0.022), but, because body weight, BMI, and height are strongly collinear (VIF > 10; Section 2.8.3), these individual coefficients are statistically unstable and are not interpreted here as independent mechanistic effects.

The remaining models with the highest explanatory power were walking speed in the ballerina condition (R^2^ = 0.290, *p* < 0.001), for which age was the only significant independent predictor (β = −0.366, *p* = 0.001), and normalised stride length in the ballerina condition (R^2^ = 0.245–0.250, *p* < 0.01). Overall, the anthropometric set explained only a modest proportion of gait variance (R^2^ ≤ 0.32 in all models), with the strongest effects in the barefoot and ballerina conditions rather than at higher heel heights.

Other conditions with high model R^2^ included the 12 cm heel condition for average swing duration (R^2^ = 0.393) and average stance duration (R^2^ = 0.393), average first double support (R^2^ = 0.383), and cadence at the 8 cm heel condition (R^2^ = 0.425). The pattern of increasing explanatory power at higher heel heights suggests that anthropometric variability becomes a stronger determinant of phase timing when the biomechanical demands of footwear are greater.

#### 3.4.3. Kruskal–Wallis Tertile Analysis

Of 360 KW tests (30 parameters × 3 variables × 4 conditions), 100 were significant (*p* < 0.05). Age tertiles accounted for the large majority of effects (81 of 100), followed by body weight (10) and height (9).

Age tertiles. Age tertile differences were most pronounced at the 12 cm heel condition. Propulsion index increased significantly with higher age tertile at 12 cm heel (avg: H = 21.02, *p* < 0.001) and 8 cm heel (avg: H = 17.55, *p* < 0.001). In contrast, stride length and normalised stride length in the ballerina condition decreased with higher age tertile (e.g., avg % stride length: H = 21.27, *p* < 0.001), indicating that younger participants achieved comparatively longer strides in flat footwear. These divergent patterns, namely greater propulsion but shorter normalised stride length with increasing age, should be interpreted cautiously given the narrow 18–32-year age span. Rather than chronological age per se, they may partly reflect unmeasured differences in cumulative heel-wearing experience, which tends to covary with age and was not recorded in this study.

Body weight tertiles. Weight tertile differences were significant for 10 parameters, predominantly in the barefoot condition. Propulsion index decreased consistently with higher weight tertile barefoot (avg: H = 13.16, *p* = 0.001), while stride duration increased (avg: H = 9.01, *p* = 0.011). The attenuation of weight-related effects in shod conditions suggests that heeled footwear may partially standardise propulsive mechanics across body weight groups.

Height tertiles. Height tertile differences were significant in nine comparisons, primarily at the 8 cm heel condition. A pattern of decreasing normalised stride length and speed with increasing height tertile was observed (e.g., avg % stride length at 8 cm heel: H = 6.09, *p* = 0.047; speed in ballerina: H = 8.05, *p* = 0.018). Although this may appear counter-intuitive given the positive Spearman correlations between height and stride duration, it is likely explained by normalisation effects: taller participants achieve similar absolute distances at slower height-normalised rates when constrained by footwear that limits natural ankle kinematics.

Across all three analytical approaches, and after FDR correction, the key finding converges: anthropometric characteristics—particularly age, body weight, and shoe size—show significant, footwear condition-dependent associations with gait mechanics. These effects are most pronounced for propulsion-related parameters and stride timing, and tend to intensify at higher heel heights, where the biomechanical demands of footwear amplify inter-individual differences attributable to body composition and morphology.

## 4. Discussion

This study investigated the effect of four footwear conditions—barefoot, ballerina flats, 8 cm heels, and 12 cm heels—on 30 spatiotemporal and pelvic gait parameters in 75 healthy young adult women using the validated G-WALK inertial measurement system [11]. The principal finding is that footwear type significantly altered the majority of gait parameters examined (22 of 30), with effects that were condition-specific and in several instances non-linear across the heel-height continuum. Additionally, a novel contribution of this work is the systematic characterisation of anthropometric influences on gait, demonstrating that age, body weight, and shoe size show modest but statistically reliable associations with several gait metrics, most consistently in the unsupported (barefoot) condition.

### 4.1. Walking Speed and Cadence

Walking speed was numerically higher in all three shod conditions than barefoot, with the increase reaching post hoc significance at the 8 cm heel (p_adj_ = 0.011). Because the effect was present across all footwear, including the flat ballerina condition, rather than scaling with heel height, it most plausibly reflects a general footwear effect (e.g., plantar protection, sole cushioning, altered plantar sensory feedback, or greater walking confidence) rather than heel elevation per se [12,13]. This increase occurred without any significant change in cadence, which remained stable between 117–119 steps/min across all conditions, indicating that the acceleration was driven primarily by stride elongation. This dissociation between speed and cadence is consistent with systematic review evidence demonstrating that heel elevation predominantly affects spatial rather than temporal gait parameters [14,15]. Recent gait studies similarly reported preserved cadence despite substantial modifications in stride mechanics during high-heeled walking [15].

The absence of a cadence effect contrasts with earlier reports suggesting that heel elevation reduces stepping rate and walking velocity [15,16,17], but aligns with findings that experienced heel wearers preserve automated stepping frequency and adapt primarily through spatial gait modifications [17,18]. The inclusion criterion requiring participants to use their own habitual footwear may have enriched the sample with experienced heel wearers, although habitual heel use was not directly quantified. The comparable walking speed observed at 8 cm and 12 cm heels suggests participants compensated at the higher elevation through increased propulsion to preserve locomotor efficiency.

The intermediate speed observed in the ballerina condition may reflect altered plantar sensory feedback associated with thin flexible footwear and reduced sole cushioning [12,13]. Previous studies comparing barefoot and minimalist footwear have shown that thin-soled shoes can preserve certain barefoot-like neuromuscular characteristics while partially attenuating plantar sensory stimulation [13]. Moreover, plantar sensory modulation has been shown to influence postural control and gait neuromechanics directly [12].

### 4.2. Stride Length and Gait Symmetry

Stride length demonstrated an inverted-U relationship with heel height, peaking at 8 cm (mean average 1.59 m; 8.9% longer than barefoot) and declining slightly at 12 cm (1.55 m). This pattern is biomechanically plausible: moderate heel elevation shifts the centre of pressure anteriorly and facilitates a more extended step trajectory, whereas excessive elevation restricts ankle dorsiflexion and constrains stride extension [14,19]. Previous plantar pressure and centre-of-pressure analyses have similarly demonstrated progressive anterior pressure redistribution with increasing heel height [19]. Additionally, elevated heels have been associated with increased frontal-plane knee loading and altered lower-limb joint moments, which may mechanically limit further stride extension at extreme elevations [20].

Normalised stride length (percentage of body height) confirmed this pattern, increasing from 87.0% barefoot to 95.1% at 8 cm, a difference of approximately 8%, confirming the stride elongation reflects a true biomechanical effect rather than anthropometric variability. The symmetry index declined progressively with increasing heel height, with a significant reduction observed between barefoot and 12 cm heels (p_adj_ = 0.025). This finding is consistent with review evidence showing that high-heeled walking introduces bilateral asymmetries in gait mechanics and plantar loading [14,21]. Sustained asymmetrical loading has been proposed as a potential contributor to musculoskeletal stress accumulation and overuse injury development [16,21].

### 4.3. Temporal Gait Structure: Stance, Swing, and Support Phases

A clinically notable finding was the distinctive temporal gait profile associated with ballerina shoes, characterised by the shortest stance duration (60.1%), longest swing phase (39.9%), lowest double support time (10.2%), and highest single support time (39.9%). This constellation reflects reduced bilateral ground contact time despite the flat, low-heel design of the footwear.

One plausible explanation is that the thin and flexible sole of ballerina shoes alters plantar sensory input and facilitates a more active push-off strategy without the substantial postural stabilisation demands imposed by heel elevation. It should be emphasised that this interpretation is an inference drawn solely from spatiotemporal parameters; because no electromyographic or force-plate data were collected, the proposed link between thin-soled plantar sensory stimulation and an altered temporal profile remains a hypothesis consistent with prior sensory-stimulation work [12,13], rather than a directly demonstrated mechanism. High-heeled shoes shift the body’s centre of mass anteriorly and increase postural control requirements during stance [14,15,18]. Previous studies have demonstrated that increasing heel height impairs postural stability, alters centre-of-gravity control, and reduces ankle strategy efficiency during standing and walking [18].

The significant reduction in double support time in the ballerina condition (10.2% vs. 11.6% barefoot; p_adj_ < 0.001) is of particular clinical interest. Double support is recognised as a key period of dynamic gait stabilisation during which weight transfer between limbs occurs [14,18]. A shorter double support phase implies faster weight transfer and a reduced stability margin; in the ballerina context this may reflect greater postural confidence and a more fluid pattern. Conversely, the return to longer double support at 12 cm heel (11.7%) is consistent with the nervous system increasing bilateral ground contact time as a compensatory stabilisation strategy under demanding conditions [14,18].

### 4.4. Propulsion Index

The propulsion index demonstrated the clearest height-dependent relationship observed in this study, increasing progressively from 8.20 barefoot to 10.11 at 12 cm heel (+23.3%). Every sequential pairwise comparison was statistically significant except barefoot versus ballerina, suggesting a strong monotonic relationship between heel height and propulsive demand.

Biomechanically, elevated heels place the ankle in a persistently plantarflexed position throughout stance, shortening the gastrocnemius–soleus complex and reducing its mechanical efficiency [22,23]. To maintain forward progression at stable walking speeds, a greater propulsive effort, captured in the current study as an elevated accelerometery-derived propulsion index, is required over a reduced ankle excursion range. Kinetic studies of high-heeled gait have demonstrated redistribution of joint power away from the ankle plantar flexors and toward proximal musculature, particularly the hip flexors during early swing [22,24].

Surface electromyographic investigations have further demonstrated altered gastrocnemius and soleus activation patterns during heeled walking [22,23], while plantar pressure studies have confirmed progressive anterior loading and increased forefoot pressure with increasing heel height [3,19]. The present findings align closely with these observations and suggest that increased propulsion requirements represent a key adaptive mechanism during heeled gait.

The clinical implications of chronically elevated propulsion demand are substantial. Long-term use of high heels has been associated with shortening of gastrocnemius fascicles, increased Achilles tendon stiffness, reduced ankle dorsiflexion range of motion, and persistent neuromechanical adaptations even during barefoot walking [23,25]. The 23.3% increase in propulsion index between barefoot and 12 cm heel supports the view that regular high heel use is consistent with an increased mechanical demand on the posterior lower-limb chain (inferred, not directly measured), with potential implications for musculoskeletal health over sustained exposure [26,27,28,29].

### 4.5. Pelvic Kinematic Symmetry

Among the three pelvic planes assessed, only obliquity symmetry demonstrated a clearly localised disruption, declining significantly at 12 cm heel relative to both ballerina and 8 cm conditions. Pelvic tilt symmetry remained stable across all conditions, whereas pelvic rotation symmetry demonstrated only a diffuse overall effect without significant post hoc pairwise differences. These results suggest a hierarchical sensitivity of pelvic planes to heel height, with the frontal plane most vulnerable to disruption at extreme elevations.

The reduction in pelvic obliquity symmetry at 12 cm heel is consistent with reports linking heeled walking to increased lateral trunk sway and altered frontal-plane pelvic motion [14,14,18]. Pelvic obliquity is strongly coupled to contralateral hip abductor activity during single-leg stance; perturbations in balance demands or lower-limb kinematics under high heel conditions may therefore manifest as asymmetric frontal-plane pelvic excursion [18,20]. The preservation of tilt symmetry may reflect compensatory postural adaptations designed to preserve sagittal alignment despite altered lower-limb biomechanics. Literature examining lumbar lordosis changes under high heels remains controversial, with some studies reporting compensatory pelvic and spinal adjustments while others demonstrate minimal sagittal adaptation [14,15].

### 4.6. Anthropometric Influences on Gait

The systematic quantification of anthropometric influences represents a novel contribution of this study. The finding that 23 of 600 Spearman correlations (3.8%) and 14 of 120 multiple linear regression models (11.7%) survived FDR correction, far exceeding chance expectation, indicates that individual morphological characteristics are associated with differences in gait responses to footwear.

Previous modelling studies have shown that lower-limb gait kinematics can be predicted from variables including walking speed, age, sex, and BMI [30]. Similarly, lower-limb length has been associated with gait-cycle timing and locomotor energetics [31].

The association was strongest in the barefoot condition (body weight × propulsion index: r = −0.41), where footwear provides no mechanical pre-loading assistance. Work on the relationship between BMI, postural control, and gait dynamics supports this interpretation, finding that both BMI and body mass correlate with support phase timing and gait mechanics [24].

The opposing directions of weight and BMI regression coefficients (standardised β weight = +1.42, β BMI = −1.64 for the average propulsion index in the barefoot condition) should be interpreted with caution: body weight, BMI, and height are strongly collinear in this sample (VIF = 42.7, 37.6, and 10.5, respectively; see Section 2.8.3), which renders the individual coefficients statistically unstable. Their opposing signs are therefore not interpreted as independent effects of lean mass versus adiposity, and no clinical inference is drawn from them here [32].

Height and shoe size showed their clearest positive associations with stride duration in the barefoot condition, consistent with the biomechanical relationship between lower-limb length and gait-cycle timing [31]. Explained variance was modest across all models (R^2^ ≤ 0.32), with the highest values observed for the barefoot propulsion index rather than for stride timing at moderate heel heights. The dominance of age effects in the Kruskal–Wallis analysis (81 of 100 significant results) suggests that even within the narrow 18–32-year range of this sample, age tertile differences in gait emerged; however, given the limited age span, these may index cumulative footwear experience as much as age itself and warrant confirmation across a wider age range.

### 4.7. Strengths and Limitations

This study has several methodological strengths. The repeated-measures within-subject design minimised inter-individual confounding and increased statistical sensitivity. The use of the validated G-WALK inertial system enabled simultaneous assessment of spatiotemporal and pelvic kinematic parameters within an ecologically realistic walking environment [11]. Additionally, the sample size of 75 participants is comparatively large for footwear biomechanics studies [14].

Several limitations should be acknowledged.

- The sample was restricted to healthy young adult women and was concentrated in the normal-to-low BMI range, with only 6 of 75 participants (8.0%) classified as overweight or obese and none who were men. Consequently, the negative associations observed between body weight (and BMI) and propulsion index should be regarded as preliminary: they characterise a predominantly lean female population and may not extend to higher-BMI individuals or to men, in whom differences in mass distribution, body composition, and lower-limb mechanics could alter—or even reverse—these relationships.
- The within-session design does not capture chronic neuromuscular adaptations associated with habitual heel use [23,25].
- Participants used their own footwear, introducing variability in sole properties beyond heel height. Because heel height was therefore confounded with sole stiffness, shoe geometry, heel base width, shoe mass, cushioning, and material properties, the observed effects reflect the combined characteristics of each footwear type rather than heel elevation in isolation. This absence of footwear standardisation is a primary limitation of the study and constrains its internal validity.
- The four footwear conditions were administered in a fixed (non-randomised) order rather than counterbalanced; consequently, order, learning, motor-adaptation, or fatigue effects cannot be excluded as contributors to the observed differences.
- Although the inclusion criteria required habitual, comfortable use of heeled footwear, the degree of habitual heel exposure (frequency, cumulative years, typical heel height) was not quantified. Prior heel-wearing experience is a plausible determinant of gait responses, particularly in the higher-heel conditions, and its absence represents an unmeasured potential confound. Statements regarding adaptation in ‘experienced’ wearers (Section 4.1) should be read in this light.
- The inertial system does not provide joint-level kinematic or kinetic data; biomechanical mechanisms proposed here are interpretations informed by the existing literature rather than directly measured phenomena [14,22,24].
- The anthropometric analysis was exploratory and involved a large number of tests. Benjamini–Hochberg FDR correction was applied within each analysis family, and only a subset of associations survived it; the surviving associations are reported as robust, but residual Type I error risk cannot be excluded and these findings remain hypothesis-generating pending confirmation in independent samples.
- With 75 participants, the statistical power of the post hoc comparisons at the Bonferroni-adjusted threshold (αadj = 0.0083) is limited for small effects—approximately 0.65–0.70 for small-to-moderate effects (Cohen’s d ≈ 0.35)—so some non-significant comparisons may reflect limited power rather than a true absence of effect. To aid interpretation, effect sizes (matched-pairs rank-biserial correlations) are reported for all post hoc comparisons (Supplementary Table S2): all significant comparisons corresponded to medium-to-large effects (|r| = 0.35–0.69), whereas non-significant comparisons showed predominantly small effects (median |r| = 0.15).
- The propulsion index is an accelerometery-derived proxy for propulsive demand rather than a force-plate measurement; absolute kinetic interpretations should therefore be made with caution.
- Shoe size as a proxy for foot length does not capture foot width or arch morphology.

### 4.8. Future Directions

Future studies should incorporate joint-level kinematic and kinetic analysis using three-dimensional motion capture with force platforms to directly measure the ankle, knee, and hip mechanics inferred here. Longitudinal designs comparing habitual and non-habitual heel wearers would clarify whether observed gait patterns reflect acute mechanical constraints or chronic neuromuscular adaptation [23,25]. The anthropometric findings, particularly the opposing roles of body mass and BMI-indexed composition in propulsion, warrant confirmation in larger samples with direct body composition measurement. Extending this paradigm to older women and clinical populations—including those with chronic ankle instability, knee osteoarthritis, or low back pain—would test generalisability and identify subgroups for whom the gait consequences of heeled footwear are most clinically significant [14].

## 5. Conclusions

This within-subject study examined the effect of four footwear conditions—barefoot, ballerina flats, 8 cm heels, and 12 cm heels—on a broad set of spatiotemporal and pelvic gait parameters in healthy young women, and assessed the associations of individual anthropometric characteristics. Footwear altered the majority of parameters examined, with effects that were condition-specific rather than uniform.

Walking accelerated in all shod conditions through stride elongation rather than a change in cadence; stride length followed an inverted-U pattern, peaking at the moderate heel and declining at the highest; ballerina flats produced a distinctly dynamic temporal profile; propulsion demand rose height-dependently; and pelvic disruption was confined to the frontal plane at the highest heel.

Importantly, anthropometric characteristics—particularly age, body weight, and shoe size—showed modest but statistically reliable associations with gait, most consistently in the unsupported (barefoot) condition rather than at the higher heel heights. Given the predominantly lean, female, young sample, these associations are best regarded as hypothesis-generating and require confirmation in more anthropometrically and demographically diverse cohorts, where biomechanical demands are greatest. Collectively, these findings show that heel height has systematic, partly non-linear, and condition-specific effects on gait that extend beyond speed and stride length to the full temporal structure of the gait cycle, propulsive mechanics, and pelvic kinematics—of relevance to future biomechanical research, clinical application requires confirmation in standardised-footwear studies and clinical populations.

## Figures and Tables

**Table 1:** Baseline characteristics of the study participants (N = 75). Data are presented as mean (95% CI [lower–upper]), calculated using Student’s t-distribution (df = 74). BMI: body mass index.

Parameter	Unit	N	Mean (95% CI [Lower–Upper])	SD	Median	Min	Max
Age (years)	years	75	24.3 (95% CI [23.5–25.2])	3.7	24.0	18.0	32.0
Height (cm)	cm	75	167.6 (95% CI [166.3–169.0])	5.8	167.0	157.0	183.0
Weight (kg)	kg	75	59.9 (95% CI [58.0–61.9])	8.4	60.0	45.0	82.0
BMI (kg/m^2^)	kg/m^2^	75	21.29 (95% CI [20.61–21.96])	2.93	21.10	15.80	30.50
European Shoe Size	-	75	37.7 (95% CI [37.4–38.0])	1.2	38.0	36.0	40.0

Note: Data are presented as mean and 95% confidence interval (CI) calculated using Student’s t-distribution (df  =  74). BMI: body mass index.

**Table 2:** Descriptive statistics and Friedman test results for primary gait parameters (N = 75). Values are mean (95% CI). Avg = bilateral average; ht = participant height. *** *p* < 0.001; ** *p* < 0.01; * *p* < 0.05; ns = not significant.

Parameter	Barefoot Mean (95% CI)	Ballerina Mean (95% CI)	8 cm Heel Mean (95% CI)	12 cm Heel Mean (95% CI)	Friedman χ^2^	*p*-Value	Sig.
**General Spatiotemporal**							
Cadence (steps/min)	118.6 (115.9–121.4)	118.5 (115.9–121.2)	117.7 (115.2–120.3)	117.4 (114.4–120.3)	1.398	0.7060	ns
Speed (m/s)	1.41 (1.34–1.48)	1.52 (1.44–1.60)	1.54 (1.48–1.60)	1.54 (1.46–1.61)	23.763	<0.0001	***
Symmetry Index	92.9 (91.3–94.4)	93.5 (92.3–94.6)	91.2 (89.3–93.2)	89.5 (87.1–91.9)	8.341	0.0395	*
Stride Duration							
Avg Stride Duration (s)	1.07 (1.02–1.12)	1.04 (1.02–1.07)	1.06 (1.03–1.08)	1.06 (1.04–1.09)	0.859	0.8353	ns
Stride Length							
Left Stride Length (m)	1.47 (1.40–1.54)	1.55 (1.47–1.63)	1.59 (1.52–1.65)	1.55 (1.49–1.61)	24.724	<0.0001	***
Right Stride Length (m)	1.45 (1.38–1.51)	1.56 (1.49–1.64)	1.59 (1.52–1.65)	1.56 (1.50–1.62)	26.093	<0.0001	***
Avg Stride Length (m)	1.46 (1.39–1.52)	1.56 (1.48–1.63)	1.59 (1.52–1.65)	1.55 (1.50–1.61)	26.414	<0.0001	***
Avg % Stride Length (% ht)	87.0 (83.0–91.0)	92.9 (88.4–97.5)	95.1 (91.3–98.8)	95.0 (91.2–98.8)	28.744	<0.0001	***
Stance and Swing Phase							
Left Stance Duration (%)	61.8 (61.0–62.6)	60.3 (59.7–60.9)	61.8 (60.9–62.8)	62.0 (60.9–63.2)	9.325	0.0253	*
Avg Stance Duration (%)	61.5 (61.1–61.9)	60.1 (59.5–60.6)	61.4 (60.7–62.0)	61.7 (60.9–62.5)	21.320	0.0001	***
Left Swing Duration (%)	38.2 (37.4–39.0)	39.7 (39.1–40.3)	38.2 (37.2–39.1)	38.0 (36.8–39.1)	9.325	0.0253	*
Avg Swing Duration (%)	38.5 (38.1–38.9)	39.9 (39.4–40.4)	38.6 (38.0–39.3)	38.3 (37.5–39.2)	21.320	0.0001	***
Double and Single Support							
First Left Double Support (%)	11.3 (10.9–11.7)	9.95 (9.3–10.6)	11.7 (10.7–12.8)	11.9 (11.1–12.8)	24.358	<0.0001	***
Avg First Double Support (%)	11.6 (11.2–12.0)	10.2 (9.7–10.7)	11.5 (10.8–12.2)	11.7 (10.9–12.5)	17.064	0.0007	***
Right Single Support (%)	38.1 (37.4–38.9)	39.6 (39.0–40.3)	38.1 (37.1–39.1)	38.0 (36.9–39.0)	10.653	0.0138	*
Avg Single Support (%)	38.5 (38.1–38.9)	39.9 (39.4–40.4)	38.6 (37.8–39.3)	38.4 (37.6–39.1)	14.775	0.0020	**

Note: *** *p* < 0.001; ** *p* < 0.01; * *p* < 0.05; ns = not significant. Post hoc Bonferroni-corrected pairwise comparisons are presented in Table 3.

**Table 3:** Significant post hoc pairwise comparisons for primary gait parameters (Wilcoxon signed-rank test, Bonferroni-corrected; α_adj_ = 0.0083; k = 6). Only significant pairs (p_adj_ < 0.05) are shown. *** *p* < 0.001; ** *p* < 0.01; * *p* < 0.05.

Parameter	Comparison	p_adj_	Sig.
Speed (m/s)	Barefoot vs. 8 cm Heel	0.011	*
	Ballerina vs. 8 cm Heel	0.037	*
Symmetry Index	Barefoot vs. 12 cm Heel	0.025	*
Avg Stride Length (m)	Barefoot vs. 8 cm Heel	0.039	*
	Ballerina vs. 8 cm Heel	0.006	**
Right Stride Length (m)	Barefoot vs. Ballerina	0.029	*
	Barefoot vs. 8 cm Heel	0.008	**
	Barefoot vs. 12 cm Heel	0.009	**
	Ballerina vs. 8 cm Heel	0.022	*
Avg % Stride Length	Barefoot vs. 8 cm Heel	0.019	*
	Barefoot vs. 12 cm Heel	0.012	*
	Ballerina vs. 8 cm Heel	0.002	**
Avg Stance Duration	Barefoot vs. Ballerina	<0.001	***
	Ballerina vs. 12 cm Heel	0.024	*
Avg Swing Duration	Barefoot vs. Ballerina	<0.001	***
	Ballerina vs. 12 cm Heel	0.024	*
Avg Double Support	Barefoot vs. Ballerina	<0.001	***
	Ballerina vs. 12 cm Heel	0.026	*
Avg Single Support	Barefoot vs. Ballerina	<0.001	***
	Ballerina vs. 12 cm Heel	0.017	*
Avg Propulsion Index	Barefoot vs. 8 cm Heel	0.011	*
	Barefoot vs. 12 cm Heel	<0.001	***
	Ballerina vs. 8 cm Heel	0.007	**
	Ballerina vs. 12 cm Heel	<0.001	***
	8 cm Heel vs. 12 cm Heel	0.011	*
Obliquity Symmetry	Ballerina vs. 8 cm Heel	0.025	*
	Ballerina vs. 12 cm Heel	0.007	**

**Table 4:** Descriptive statistics and Friedman test results for secondary gait parameters: propulsion index and pelvic kinematic symmetry indices (N = 75). Values are mean (95% CI). *** *p* < 0.001; ** *p* < 0.01; ns = not significant.

Parameter	Barefoot Mean (95% CI)	Ballerina Mean (95% CI)	8 cm Heel Mean (95% CI)	12 cm Heel Mean (95% CI)	Friedman χ^2^	*p*-Value	Sig.
Propulsion Index							
Left Propulsion Index	8.23 (7.78–8.67)	8.73 (8.26–9.21)	9.17 (8.61–9.73)	10.25 (9.50–11.00)	27.67	<0.001	***
Right Propulsion Index	8.18 (7.65–8.71)	8.56 (8.00–9.13)	9.30 (8.68–9.92)	9.97 (9.26–10.67)	29.92	<0.001	***
Avg Propulsion Index	8.20 (7.76–8.65)	8.65 (8.15–9.14)	9.23 (8.69–9.78)	10.11 (9.46–10.76)	29.94	<0.001	***
Pelvic Kinematic Symmetry Indices							
Tilt Symmetry Index	66.89 (62.64–71.14)	66.90 (62.04–71.75)	63.08 (58.09–68.08)	64.49 (59.20–69.78)	2.78	0.426	ns
Obliquity Symmetry Index	95.17 (93.37–96.98)	95.88 (93.71–98.06)	95.92 (95.24–96.60)	93.66 (92.05–95.26)	18.11	<0.001	***
Rotation Symmetry Index	90.12 (85.99–94.24)	91.69 (89.22–94.16)	91.43 (88.65–94.22)	89.61 (86.88–92.35)	13.44	0.004	**

Note: *** *p* < 0.001; ** *p* < 0.01; ns = not significant. Post hoc Bonferroni-corrected pairwise comparisons are presented in Table 5.

**Table 5:** Significant post hoc pairwise comparisons for secondary gait parameters (Wilcoxon signed-rank test, Bonferroni-corrected; α_adj_ = 0.0083). Only significant pairs (p_adj_ < 0.05) are shown. Pelvic tilt symmetry index: no significant pairs. Pelvic rotation symmetry index: significant overall Friedman effect (χ^2^ = 13.44, *p* = 0.004) but no pair survived Bonferroni correction. *** *p* < 0.001; ** *p* < 0.01; * *p* < 0.05.

Parameter	Comparison	*p* (adj.)	Sig.
Left Propulsion Index	Barefoot vs. 12 cm Heel	<0.0001	***
	Ballerina vs. 12 cm Heel	<0.0001	***
	8 cm Heel vs. 12 cm Heel	0.0134	*
Right Propulsion Index	Barefoot vs. 8 cm Heel	0.0208	*
	Barefoot vs. 12 cm Heel	<0.0001	***
	Ballerina vs. 8 cm Heel	0.0258	*
	Ballerina vs. 12 cm Heel	0.0004	***
Avg Propulsion Index	Barefoot vs. 8 cm Heel	0.0113	*
	Barefoot vs. 12 cm Heel	<0.0001	***
	Ballerina vs. 8 cm Heel	0.0065	**
	Ballerina vs. 12 cm Heel	<0.0001	***
	8 cm Heel vs. 12 cm Heel	0.0111	*
Obliquity Symmetry Index	Ballerina vs. 8 cm Heel	0.0245	*
	Ballerina vs. 12 cm Heel	0.0072	**

## Data Availability

All data generated is included in the Supplementary Materials section as Table S1: Raw data generated by the study “Footwear Heel Height and Gait Biomechanics in Healthy Young Women: A Within-Subject Analysis of Spatiotemporal Parameters, Propulsion, Pelvic Kinematics, and the Modulating Role of Anthropometric Characteristics”.

## Supplementary Materials

The following supporting information can be downloaded at: https://www.mdpi.com/article/10.3390/life16060977/s1, Table S1: Raw data. Complete participant-level dataset generated by the study “Footwear Heel Height and Gait Biomechanics in Healthy Young Women: A Within-Subject Analysis of Spatiotemporal Parameters, Propulsion, Pelvic Kinematics, and the Modulating Role of Anthropometric Characteristics”. Each of the N = 75 healthy young women was assessed under four footwear conditions (Barefoot, Ballerina, 8 cm Heel, 12 cm Heel), yielding 300 observations (75 × 4). Columns are grouped by domain: identification, anthropometric demographics, general gait, and spatiotemporal/propulsion/pelvic-kinematic gait parameters. “Left/Right” denote the corresponding limb and “Avg” the bilateral mean. Rows are ordered by footwear condition (blocks of 75) and, within each block, by participant ID; horizontal rules separate the condition blocks. Table S2: Effect sizes for post-hoc pairwise comparisons (Wilcoxon signed-rank). Matched-pairs rank-biserial correlation (r) for each pairwise footwear comparison; |r|~0.1/0.3/0.5 denote small/medium/large effects. p-adj = Bonferroni-corrected *p*-value (x6), significant at p-adj < 0.05. N = 75; df = 74. Significant rows are shaded.
