# Supplementary material for: Footwear Heel Height and Gait Biomechanics in Healthy Young Women: A Within-Subject Analysis of Spatiotemporal Parameters, Propulsion, and Pelvic Kinematics
Source: Life (Basel). 2026 Jun 10;16(6):977. doi: 10.3390/life16060977 (PMC13300841; doi:10.3390/life16060977)
Supplement: Supplementary file 1 [file life-16-00977-s001.zip › Table_S1_Raw_data.pdf]

## Table S1. Raw data

Complete participant-level dataset generated by the study “*Footwear Heel Height and Gait Biomechanics in Healthy Young Women: A Within-Subject Analysis of Spatiotemporal Parameters, Propulsion, Pelvic Kinematics, and the Modulating Role of Anthropometric Characteristics*”. Each of the N = 75 healthy young women was assessed under four footwear conditions (Barefoot, Ballerina, 8 cm Heel, 12 cm Heel), yielding 300 observations (75 × 4). Columns are grouped by domain: identification, anthropometric demographics, general gait, and spatiotemporal / propulsion / pelvic-kinematic gait parameters. “Left/Right” denote the corresponding limb and “Avg” the bilateral mean. Rows are ordered by footwear condition (blocks of 75) and, within each block, by participant ID; horizontal rules separate the condition blocks.

| IDENTIFICATION | | DEMOGRAPHICS | | | | | GENERAL GAIT | | | Stride Duration (s) | | | Stride Length (m) | | | % Stride Length (% height) | | | Stance Duration (%) | | | Swing Duration (%) | | | Double Support (%) | | | Single Support (%) | | | Propulsion Index | | | Pelvis Symmetry (Symmetry Index) | | |
|---|---|---|---|---|---|---|---|---|---|---|---|---|---|---|---|---|---|---|---|---|---|---|---|---|---|---|---|---|---|---|---|---|---|---|---|---|
| ID | Condition | Age (yr) | Height (cm) | Weight (kg) | BMI (kg/m$^2$) | Shoe Size (EU) | Cadence (steps/min) | Speed (m/s) | Symmetry Index | Left | Right | Avg | Left | Right | Avg | Left | Right | Avg | Left | Right | Avg | Left | Right | Avg | First Left | First Right | Avg First | Left | Right | Avg | Left | Right | Avg | Tilt | Obliquity | Rotation |
| 1 | Barefoot | 18 | 162 | 57 | 21.7 | 39 | 138.8 | 2.16 | 91.2 | 0.92 | 0.9 | 0.91 | 2.15 | 1.77 | 1.96 | 130.9 | 107.9 | 119.4 | 62.7 | 61 | 61.85 | 37.3 | 39 | 38.15 | 9.8 | 9.6 | 9.7 | 42 | 42.8 | 42.4 | 6.1 | 5 | 5.55 | 71.4 | 93.2 | 89.8 |
| 2 | Barefoot | 19 | 161 | 52 | 20.1 | 38 | 122.3 | 1.55 | 92 | 1.07 | 1.09 | 1.08 | 1.63 | 1.64 | 1.635 | 101.7 | 102.3 | 102 | 53.2 | 67.1 | 60.15 | 46.8 | 32.9 | 39.85 | 11.8 | 9.5 | 10.65 | 31.6 | 45.4 | 38.5 | 9.5 | 6.8 | 8.15 | 38 | 97.2 | 94.2 |
| 3 | Barefoot | 19 | 165 | 53 | 19.5 | 39 | 102.6 | 1.77 | 96.6 | 1.51 | 1.42 | 1.465 | 1.99 | 2.37 | 2.18 | 120.5 | 143.9 | 132.2 | 62.7 | 51.9 | 57.3 | 37.3 | 48.1 | 42.7 | 7.5 | 8.9 | 8.2 | 46.2 | 35.3 | 40.75 | 6.5 | 7.3 | 6.9 | 73.4 | 94.5 | 96.7 |
| 4 | Barefoot | 19 | 168 | 48 | 17 | 37 | 131.3 | 2.04 | 97.6 | 0.92 | 0.93 | 0.925 | 1.8 | 1.97 | 1.885 | 109.2 | 119.2 | 114.2 | 59.8 | 58.6 | 59.2 | 40.2 | 41.4 | 40.8 | 8.3 | 9.7 | 9 | 42.4 | 40.6 | 41.5 | 10 | 8.1 | 9.05 | 77.4 | 97.4 | 97.5 |
| 5 | Barefoot | 19 | 169 | 61 | 21.4 | 38 | 116.9 | 1.23 | 97.7 | 1.03 | 1.02 | 1.025 | 1.27 | 1.26 | 1.265 | 74.5 | 73.8 | 74.15 | 63.4 | 63.5 | 63.45 | 36.6 | 36.5 | 36.55 | 12.3 | 13.7 | 13 | 36.3 | 37.5 | 36.9 | 10.2 | 9.2 | 9.7 | 63.1 | 98.3 | 98.1 |
| 6 | Barefoot | 19 | 172 | 59 | 19.9 | 39 | 107.1 | 0.71 | 89.5 | 1.2 | 1.22 | 1.21 | 0.85 | 0.86 | 0.855 | 50.2 | 50.6 | 50.4 | 57.4 | 63.2 | 60.3 | 42.6 | 36.8 | 39.7 | 9.8 | 9.8 | 9.8 | 37.6 | 42 | 39.8 | 8 | 5.8 | 6.9 | 59 | 47.8 | 28.4 |
| 7 | Barefoot | 19 | 172 | 69 | 23.3 | 40 | 151.2 | 2.3 | 60.4 | 0.93 | 0.73 | 0.83 | 2.02 | 1.85 | 1.935 | 116.7 | 106.9 | 111.8 | 49.9 | 79.5 | 64.7 | 50.1 | 20.5 | 35.3 | 13.4 | 16.4 | 14.9 | 27 | 47.9 | 37.45 | 13.6 | 8.5 | 11.05 | 44.4 | 65.8 | 81.3 |
| 8 | Barefoot | 19 | 173 | 67 | 22.4 | 38 | 105.8 | 1.35 | 88 | 1.22 | 1.23 | 1.225 | 1.64 | 1.59 | 1.615 | 93.7 | 90.7 | 92.2 | 58.4 | 63.3 | 60.85 | 41.6 | 36.7 | 39.15 | 11.4 | 10.6 | 11 | 36.5 | 41.3 | 38.9 | 7.6 | 6.8 | 7.2 | 54.5 | 95.8 | 80 |
| 9 | Barefoot | 20 | 159 | 77 | 30.5 | 37 | 120.2 | 1.18 | 96.2 | 1.04 | 1 | 1.02 | 1.21 | 1.21 | 1.21 | 74.5 | 74.4 | 74.45 | 61.7 | 58.7 | 60.2 | 38.3 | 41.3 | 39.8 | 11.9 | 8.8 | 10.35 | 41.2 | 39.6 | 40.4 | 4.8 | 6.1 | 5.45 | 67.8 | 98.3 | 99.3 |
| 10 | Barefoot | 20 | 163 | 61 | 23 | 39 | 92 | 1.51 | 87.9 | 1.48 | 1.39 | 1.435 | 2.05 | 2.16 | 2.105 | 123.5 | 129.9 | 126.7 | 53.5 | 65.8 | 59.65 | 46.5 | 34.2 | 40.35 | 11.1 | 11 | 11.05 | 34.4 | 42.4 | 38.4 | 6.8 | 6.3 | 6.55 | 81.1 | 85.1 | 65.5 |
| 11 | Barefoot | 20 | 164 | 76 | 28.3 | 39 | 114.8 | 1.44 | 92.4 | 1.04 | 1.05 | 1.045 | 1.5 | 1.51 | 1.505 | 92.4 | 93.2 | 92.8 | 66.8 | 57.9 | 62.35 | 33.2 | 42.1 | 37.65 | 14.3 | 15.1 | 14.7 | 37 | 32.8 | 34.9 | 7.1 | 7 | 7.05 | 65.9 | 98 | 98.8 |
| 12 | Barefoot | 20 | 167 | 58 | 20.8 | 37 | 122.3 | 1.51 | 89 | 1.1 | 1.16 | 1.13 | 1.55 | 1.55 | 1.55 | 94.8 | 94.5 | 94.65 | 65.2 | 60.4 | 62.8 | 34.8 | 39.6 | 37.2 | 13.4 | 12.5 | 12.95 | 38.2 | 34.2 | 36.2 | 6.7 | 8.7 | 7.7 | 77.6 | 90.9 | 46.6 |
| 13 | Barefoot | 20 | 169 | 65 | 22.8 | 37 | 102 | 1.55 | 96.7 | 1.17 | 1.19 | 1.18 | 1.9 | 1.76 | 1.83 | 114.7 | 105.9 | 110.3 | 63 | 61.2 | 62.1 | 37 | 38.8 | 37.9 | 11.8 | 11.6 | 11.7 | 39.2 | 36.5 | 37.85 | 6.1 | 5.5 | 5.8 | 70.7 | 98.5 | 94.2 |
| 14 | Barefoot | 21 | 158 | 57 | 22.8 | 36 | 133.4 | 1.57 | 97.1 | 0.9 | 0.89 | 0.895 | 1.38 | 1.41 | 1.395 | 86.5 | 88.4 | 87.45 | 63.1 | 59.9 | 61.5 | 36.9 | 40.1 | 38.5 | 12.2 | 12.4 | 12.3 | 38.1 | 36 | 37.05 | 12.2 | 12.5 | 12.35 | 94.8 | 98.2 | 99.3 |
| 15 | Barefoot | 21 | 163 | 57 | 21.5 | 38 | 138.2 | 1.65 | 95.6 | 0.98 | 0.96 | 0.97 | 1.55 | 1.53 | 1.54 | 94.4 | 93.3 | 93.85 | 61.4 | 63.8 | 62.6 | 38.6 | 36.2 | 37.4 | 11.7 | 11.9 | 11.8 | 37.9 | 39.4 | 38.65 | 8.1 | 7.6 | 7.85 | 86.1 | 93.9 | 92.1 |
| 16 | Barefoot | 21 | 165 | 60 | 22 | 37 | 133.3 | 2.64 | 92.6 | 0.94 | 0.91 | 0.925 | 2.66 | 2.17 | 2.415 | 162.2 | 132.2 | 147.2 | 62.8 | 62.1 | 62.45 | 37.2 | 37.9 | 37.55 | 9.2 | 13.4 | 11.3 | 39.9 | 38.8 | 39.35 | 6.6 | 6.3 | 6.45 | 81.5 | 95.3 | 90.2 |
| 17 | Barefoot | 21 | 165 | 63 | 23.1 | 38 | 131 | 1.35 | 69.8 | 1.01 | 1.01 | 1.01 | 1.36 | 1.38 | 1.37 | 83.1 | 84.1 | 83.6 | 63 | 68.8 | 65.9 | 37 | 31.2 | 34.1 | 15.4 | 15.8 | 15.6 | 32.3 | 37.2 | 34.75 | 6.3 | 4.7 | 5.5 | 31.8 | 68.9 | 29.6 |
| 18 | Barefoot | 21 | 172 | 73 | 24.7 | 40 | 122.6 | 1.5 | 91.9 | 1.02 | 0.93 | 0.975 | 1.46 | 1.47 | 1.465 | 84.3 | 84.8 | 84.55 | 59 | 62.2 | 60.6 | 41 | 37.8 | 39.4 | 11.8 | 12.4 | 12.1 | 36.6 | 38.4 | 37.5 | 9 | 7.3 | 8.15 | 54.2 | 97.2 | 94.4 |
| 19 | Barefoot | 21 | 176 | 62 | 20 | 37 | 121.2 | 1.19 | 90.6 | 1.01 | 1.02 | 1.015 | 1.2 | 1.19 | 1.195 | 68.5 | 68.1 | 68.3 | 61.8 | 59.3 | 60.55 | 38.2 | 40.7 | 39.45 | 11.9 | 7.8 | 9.85 | 41.6 | 38.7 | 40.15 | 7.4 | 6.1 | 6.75 | 84.8 | 96.7 | 94.8 |
| 20 | Barefoot | 21 | 177 | 65 | 20.7 | 39 | 103.2 | 1.43 | 93.8 | 1.17 | 1.21 | 1.19 | 1.68 | 1.69 | 1.685 | 96 | 96.7 | 96.35 | 61.3 | 58.3 | 59.8 | 38.7 | 41.7 | 40.2 | 9.7 | 9.9 | 9.8 | 41.3 | 38.3 | 39.8 | 8.8 | 8.9 | 8.85 | 73.7 | 98.1 | 90.6 |
| 21 | Barefoot | 22 | 157 | 51 | 20.7 | 36 | 147.2 | 1.84 | 78.2 | 0.9 | 0.93 | 0.915 | 1.56 | 1.58 | 1.57 | 97.7 | 98.8 | 98.25 | 66.1 | 58.3 | 62.2 | 33.9 | 41.7 | 37.8 | 12.7 | 12.6 | 12.65 | 39.3 | 35 | 37.15 | 8.9 | 6.4 | 7.65 | 41.9 | 92.3 | 93.4 |
| 22 | Barefoot | 22 | 162 | 52 | 19.8 | 38 | 126.8 | 1.4 | 96.1 | 1.06 | 0.94 | 1 | 1.29 | 1.52 | 1.405 | 78.3 | 91.9 | 85.1 | 53.2 | 65.2 | 59.2 | 46.8 | 34.8 | 40.8 | 8.4 | 11.5 | 9.95 | 35.3 | 43.9 | 39.6 | 5.8 | 6.8 | 6.3 | 70 | 89.3 | 95.2 |
| 23 | Barefoot | 22 | 162 | 53 | 20.2 | 39 | 117.3 | 1.47 | 98 | 1.03 | 1 | 1.015 | 1.42 | 1.54 | 1.48 | 86.7 | 93.8 | 90.25 | 59.4 | 60.5 | 59.95 | 40.6 | 39.5 | 40.05 | 10.7 | 11.5 | 11.1 | 38.3 | 38.1 | 38.2 | 7.3 | 8.6 | 7.95 | 88.2 | 98.6 | 88.4 |
| 24 | Barefoot | 22 | 162 | 56 | 21.3 | 37 | 123.8 | 1.32 | 97.9 | 0.99 | 0.97 | 0.98 | 1.3 | 1.28 | 1.29 | 81 | 80.2 | 80.6 | 63.5 | 60.5 | 62 | 36.5 | 39.5 | 38 | 11.5 | 13.1 | 12.3 | 39.2 | 37.1 | 38.15 | 9.7 | 10.2 | 9.95 | 72.7 | 98.9 | 93 |
| 25 | Barefoot | 22 | 168 | 63 | 22.3 | 38 | 115.8 | 1.99 | 88 | 1.11 | 1.1 | 1.105 | 2.15 | 2.1 | 2.125 | 126.5 | 123.6 | 125.05 | 56.5 | 62.6 | 59.55 | 43.5 | 37.4 | 40.45 | 13.2 | 7.4 | 10.3 | 35.7 | 42.4 | 39.05 | 7.2 | 5.7 | 6.45 | 61.2 | 93.4 | 80.5 |
| 26 | Barefoot | 22 | 168 | 63 | 22.3 | 39 | 118.1 | 1.46 | 96.6 | 1.08 | 1.03 | 1.055 | 1.5 | 1.56 | 1.53 | 88.4 | 92 | 90.2 | 62.8 | 65.3 | 64.05 | 37.2 | 34.7 | 35.95 | 13 | 15 | 14 | 35.7 | 38.6 | 37.15 | 9.7 | 8 | 8.85 | 94.3 | 98.2 | 97.6 |
| 27 | Barefoot | 22 | 170 | 64 | 22.1 | 37 | 119.7 | 1.29 | 83.3 | 1.12 | 1.05 | 1.085 | 1.44 | 1.35 | 1.395 | 84.7 | 79.3 | 82 | 56.2 | 65.8 | 61 | 43.8 | 34.2 | 39 | 10.1 | 12.7 | 11.4 | 33.6 | 42.5 | 38.05 | 8.5 | 6.9 | 7.7 | 64.9 | 86.9 | 42.4 |
| 28 | Barefoot | 22 | 173 | 59 | 19.7 | 37 | 112.2 | 1.18 | 98.7 | 1.07 | 1.07 | 1.07 | 1.26 | 1.27 | 1.265 | 74 | 74.9 | 74.45 | 62.2 | 62.2 | 62.2 | 37.8 | 37.8 | 37.8 | 11.4 | 12 | 11.7 | 37.5 | 38.8 | 38.15 | 10 | 8.6 | 9.3 | 48.9 | 98.9 | 97.4 |
| 29 | Barefoot | 23 | 163 | 81 | 30.5 | 38 | 122.4 | 1.22 | 89.3 | 1.05 | 1.04 | 1.045 | 1.26 | 1.26 | 1.26 | 77.7 | 77.6 | 77.65 | 68.3 | 62.1 | 65.2 | 31.7 | 37.9 | 34.8 | 17.9 | 13.4 | 15.65 | 38.2 | 30.1 | 34.15 | 6.6 | 8.4 | 7.5 | 57.5 | 95.8 | 97.7 |
| 30 | Barefoot | 23 | 164 | 48 | 17.8 | 38 | 118 | 1.22 | 99 | 1.02 | 1.02 | 1.02 | 1.24 | 1.26 | 1.25 | 75.5 | 77 | 76.25 | 61.6 | 62.3 | 61.95 | 38.4 | 37.7 | 38.05 | 12 | 12.8 | 12.4 | 38.1 | 37.4 | 37.75 | 10 | 9.7 | 9.85 | 89.3 | 97.2 | 99.4 |
| 31 | Barefoot | 23 | 166 | 60 | 21.8 | 38 | 99.2 | 1.33 | 95.9 | 1.24 | 1.21 | 1.225 | 1.69 | 1.51 | 1.6 | 101.8 | 90.9 | 96.35 | 63.4 | 61.5 | 62.45 | 36.6 | 38.5 | 37.55 | 13.2 | 11.5 | 12.35 | 38.6 | 37 | 37.8 | 5 | 7.2 | 6.1 | 71.6 | 99.1 | 94.2 |
| 32 | Barefoot | 23 | 167 | 50 | 17.9 | 37 | 122.2 | 1.34 | 94.7 | 0.99 | 0.98 | 0.985 | 1.32 | 1.33 | 1.325 | 77.4 | 78.2 | 77.8 | 63.9 | 61.1 | 62.5 | 36.1 | 38.9 | 37.5 | 12.9 | 12.3 | 12.6 | 38.4 | 35.8 | 37.1 | 9.1 | 8.5 | 8.8 | 28.2 | 98.1 | 99 |
| 33 | Barefoot | 23 | 172 | 57 | 19.3 | 39 | 115 | 1.29 | 93.2 | 1.04 | 1.04 | 1.04 | 1.34 | 1.34 | 1.34 | 77.3 | 77.4 | 77.35 | 61.6 | 61.1 | 61.35 | 38.4 | 38.9 | 38.65 | 9.2 | 14.2 | 11.7 | 38.6 | 37.6 | 38.1 | 8.2 | 9 | 8.6 | 74.2 | 98.4 | 99 |
| 34 | Barefoot | 23 | 175 | 53 | 17.3 | 38 | 112.1 | 1.3 | 95.1 | 1.07 | 1.04 | 1.055 | 1.39 | 1.37 | 1.38 | 80.1 | 79.3 | 79.7 | 59.7 | 62.6 | 61.15 | 40.3 | 37.4 | 38.85 | 10.5 | 11.8 | 11.15 | 37.3 | 39.3 | 38.3 | 8.7 | 7.9 | 8.3 | 75.7 | 95.2 | 98.2 |
| 35 | Barefoot | 23 | 175 | 63 | 20.6 | 37 | 110.8 | 1.48 | 95.8 | 1.08 | 1.09 | 1.085 | 1.64 | 1.55 | 1.595 | 93.9 | 88.8 | 91.35 | 59.3 | 62.7 | 61 | 40.7 | 37.3 | 39 | 12 | 11.1 | 11.55 | 36.1 | 40.6 | 38.35 | 10.7 | 9.3 | 10 | 40.4 | 98 | 98.6 |
| 36 | Barefoot | 23 | 175 | 68 | 22.2 | 39 | 130.1 | 1.69 | 89.6 | 0.98 | 0.93 | 0.955 | 1.62 | 1.6 | 1.61 | 93.5 | 92.4 | 92.95 | 60.4 | 63.2 | 61.8 | 39.6 | 36.8 | 38.2 | 13.3 | 10.2 | 11.75 | 37.4 | 41.7 | 39.55 | 8.3 | 8 | 8.15 | 79.8 | 95.6 | 93 |
| 37 | Barefoot | 24 | 159 | 56 | 22.2 | 36 | 132.7 | 1.48 | 95.7 | 0.92 | 0.91 | 0.915 | 1.34 | 1.37 | 1.355 | 83.7 | 85.7 | 84.7 | 63.5 | 60.2 | 61.85 | 36.5 | 39.8 | 38.15 | 12.5 | 10.6 | 11.55 | 39.5 | 37.5 | 38.5 | 10.2 | 11.7 | 10.95 | 82.3 | 98.3 | 90 |
| 38 | Barefoot | 24 | 163 | 61 | 23 | 37 | 111.3 | 1.21 | 97.7 | 2.37 | 2.58 | 2.475 | 1.38 | 1.4 | 1.39 | 86.4 | 87.8 | 87.1 | 62 | 55.8 | 58.9 | 38 | 44.2 | 41.1 | 8.4 | 10 | 9.2 | 44.3 | 36.4 | 40.35 | 10.4 | 9 | 9.7 | 89.7 | 98.4 | 82 |
| 39 | Barefoot | 24 | 166 | 51 | 18.5 | 36 | 122 | 1.38 | 98.3 | 0.98 | 0.99 | 0.985 | 1.35 | 1.37 | 1.36 | 82.2 | 83.4 | 82.8 | 61.9 | 64.5 | 63.2 | 38.1 | 35.5 | 36.8 | 12 | 12.4 | 12.2 | 37.4 | 37.8 | 37.6 | 11.2 | 9.8 | 10.5 | 95.6 | 96.6 | 98 |
| 40 | Barefoot | 24 | 167 | 46 | 16.5 | 36 | 131.4 | 1.48 | 73.1 | 0.91 | 0.91 | 0.91 | 1.33 | 1.35 | 1.34 | 81.3 | 82.5 | 81.9 | 62.8 | 56.6 | 59.7 | 37.2 | 43.4 | 40.3 | 12.1 | 8.6 | 10.35 | 42.7 | 36.4 | 39.55 | 12.9 | 15.3 | 14.1 | 29.2 | 92.8 | 0.9 |
| 41 | Barefoot | 24 | 171 | 55 | 18.8 | 38 | 125.2 | 1.39 | 92 | 0.96 | 0.97 | 0.965 | 1.34 | 1.36 | 1.35 | 78.7 | 80.1 | 79.4 | 60.9 | 58.7 | 59.8 | 39.1 | 41.3 | 40.2 | 11.4 | 9.8 | 10.6 | 40.1 | 38.7 | 39.4 | 8.9 | 7.8 | 8.35 | 21.5 | 97 | 86.5 |
| 42 | Barefoot | 24 | 172 | 61 | 20.6 | 38 | 123.8 | 1.13 | 91.2 | 1 | 0.95 | 0.975 | 1.11 | 1.09 | 1.1 | 63.5 | 62.2 | 62.85 | 59.9 | 66.2 | 63.05 | 40.1 | 33.8 | 36.95 | 10.5 | 12.3 | 11.4 | 35.3 | 42.4 | 38.85 | 8.7 | 5 | 6.85 | 72.3 | 97.1 | 97.5 |
| 43 | Barefoot | 24 | 177 | 66 | 21.1 | 39 | 124 | 1.12 | 95 | 0.99 | 1 | 0.995 | 1.11 | 1.1 | 1.105 | 63.7 | 63 | 63.35 | 61.9 | 64.4 | 63.15 | 38.1 | 35.6 | 36.85 | 11.5 | 13.2 | 12.35 | 37.9 | 39.1 | 38.5 | 7.6 | 5.8 | 6.7 | 70.2 | 97.4 | 98.4 |
| 44 | Barefoot | 25 | 162 | 54 | 20.6 | 37 | 114.6 | 1.17 | 98.8 | 1.05 | 1.05 | 1.05 | 1.21 | 1.23 | 1.22 | 73.8 | 74.9 | 74.35 | 62 | 66.3 | 64.15 | 38 | 33.7 | 35.85 | 14.9 | 12.2 | 13.55 | 34.9 | 38 | 36.45 | 9.8 | 8.7 | 9.25 | 97.9 | 97.2 | 99.3 |
| 45 | Barefoot | 25 | 169 | 59 | 20.7 | 38 | 124.4 | 1.29 | 94.5 | 0.97 | 0.98 | 0.975 | 1.25 | 1.25 | 1.25 | 73.2 | 72.9 | 73.05 | 63.3 | 57.7 | 60.5 | 36.7 | 42.3 | 39.5 | 9.9 | 10.8 | 10.35 | 42.5 | 36.7 | 39.6 | 6.1 | 8.8 | 7.45 | 77.1 | 98 | 88.2 |
| 46 | Barefoot | 25 | 172 | 54 | 18.3 | 36 | 116 | 1.18 | 96.5 | 1.04 | 1.04 | 1.04 | 1.21 | 1.22 | 1.215 | 71.4 | 71.8 | 71.6 | 61.8 | 62.6 | 62.2 | 38.2 | 37.4 | 37.8 | 12.6 | 11.6 | 12.1 | 37.7 | 38.2 | 37.95 | 8.9 | 7.5 | 8.2 | 59.7 | 96.9 | 99.4 |
| 47 | Barefoot | 26 | 162 | 47 | 17.9 | 36 | 131.4 | 1.55 | 81.4 | 0.91 | 0.9 | 0.905 | 1.41 | 1.39 | 1.4 | 86 | 84.8 | 85.4 | 61.9 | 56.7 | 59.3 | 38.1 | 43.3 | 40.7 | 10.6 | 8.6 | 9.6 | 42.9 | 37.5 | 40.2 | 12.2 | 18.3 | 15.25 | 58.1 | 96.2 | 98.1 |

| IDENTIFICATION | | DEMOGRAPHICS | | | | | GENERAL GAIT | | | Stride Duration (s) | | | Stride Length (m) | | | % Stride Length (% height) | | | Stance Duration (%) | | | Swing Duration (%) | | | Double Support (%) | | | Single Support (%) | | | Propulsion Index | | | Pelvis Symmetry (Symmetry Index) | | |
|---|---|---|---|---|---|---|---|---|---|---|---|---|---|---|---|---|---|---|---|---|---|---|---|---|---|---|---|---|---|---|---|---|---|---|---|---|
| ID | Condition | Age (yr) | Height (cm) | Weight (kg) | BMI (kg/m$^2$) | Shoe Size (EU) | Cadence (steps/min) | Speed (m/s) | Symmetry Index | Left | Right | Avg | Left | Right | Avg | Left | Right | Avg | Left | Right | Avg | Left | Right | Avg | First Left | First Right | Avg First | Left | Right | Avg | Left | Right | Avg | Tilt | Obliquity | Rotation |
| 48 | Barefoot | 26 | 164 | 50 | 18.6 | 37 | 118.3 | 1.24 | 96 | 1.01 | 1.01 | 1.01 | 1.25 | 1.26 | 1.255 | 75.6 | 76.1 | 75.85 | 64.6 | 62 | 63.3 | 35.4 | 38 | 36.7 | 10.7 | 15.6 | 13.15 | 37.9 | 35.1 | 36.5 | 8.9 | 10.5 | 9.7 | 62.2 | 99.2 | 97.8 |
| 49 | Barefoot | 26 | 169 | 58 | 20.3 | 37 | 121 | 1.34 | 95.5 | 1 | 1.01 | 1.005 | 1.31 | 1.36 | 1.335 | 75.6 | 78.9 | 77.25 | 62.1 | 64.5 | 63.3 | 37.9 | 35.5 | 36.7 | 13.1 | 12.7 | 12.9 | 35.9 | 38.8 | 37.35 | 10.8 | 8.7 | 9.75 | 85.9 | 98.3 | 97.6 |
| 50 | Barefoot | 26 | 172 | 55 | 18.6 | 37 | 124.2 | 1.31 | 97.5 | 0.97 | 0.97 | 0.97 | 1.27 | 1.27 | 1.27 | 74.3 | 74.1 | 74.2 | 61.4 | 59.6 | 60.5 | 38.6 | 40.4 | 39.5 | 11 | 10 | 10.5 | 40.4 | 38.5 | 39.45 | 7.1 | 7.8 | 7.45 | 85 | 99 | 95.9 |
| 51 | Barefoot | 26 | 173 | 77 | 25.7 | 40 | 95.6 | 1.29 | 94.1 | 1.33 | 1.4 | 1.365 | 1.73 | 1.55 | 1.64 | 98.9 | 88.3 | 93.6 | 58.3 | 61.3 | 59.8 | 41.7 | 38.7 | 40.2 | 10.5 | 9.8 | 10.15 | 38.8 | 41.5 | 40.15 | 7.1 | 5.9 | 6.5 | 50.6 | 94.9 | 79.5 |
| 52 | Barefoot | 27 | 158 | 61 | 24.4 | 37 | 118.7 | 1.25 | 94.9 | 1.02 | 1.01 | 1.015 | 1.28 | 1.27 | 1.275 | 79.3 | 78.7 | 79 | 66 | 55.9 | 60.95 | 34 | 44.1 | 39.05 | 9.4 | 14.8 | 12.1 | 41.9 | 33.9 | 37.9 | 8.1 | 8.6 | 8.35 | 67.8 | 99.1 | 99 |
| 53 | Barefoot | 27 | 162 | 64 | 24.4 | 37 | 108.4 | 1.1 | 94.2 | 1.11 | 1.16 | 1.135 | 1.21 | 1.3 | 1.255 | 74.1 | 79.6 | 76.85 | 62 | 58.2 | 60.1 | 38 | 41.8 | 39.9 | 11.4 | 11.7 | 11.55 | 40.9 | 36.5 | 38.7 | 7.4 | 8.4 | 7.9 | 71.5 | 95.2 | 97.3 |
| 54 | Barefoot | 27 | 163 | 51 | 19.2 | 36 | 120.4 | 1.34 | 94.9 | 1 | 1 | 1 | 1.33 | 1.33 | 1.33 | 80.9 | 80.8 | 80.85 | 61.2 | 57.4 | 59.3 | 38.8 | 42.6 | 40.7 | 8.4 | 10.3 | 9.35 | 42.4 | 38.8 | 40.6 | 8.9 | 10.4 | 9.65 | 59.7 | 99 | 98.9 |
| 55 | Barefoot | 27 | 164 | 66 | 24.5 | 36 | 128.2 | 1.42 | 95.5 | 0.94 | 0.94 | 0.94 | 1.35 | 1.33 | 1.34 | 84.1 | 82.3 | 83.2 | 67.5 | 61.9 | 64.7 | 32.5 | 38.1 | 35.3 | 13 | 16 | 14.5 | 38.6 | 32.7 | 35.65 | 8.8 | 8.4 | 8.6 | 54.9 | 99.3 | 98 |
| 56 | Barefoot | 27 | 172 | 57 | 19.3 | 39 | 130.3 | 1.41 | 92.1 | 0.94 | 0.93 | 0.935 | 1.3 | 1.33 | 1.315 | 76.3 | 77.8 | 77.05 | 62.7 | 57.3 | 60 | 37.3 | 42.7 | 40 | 10.8 | 9.7 | 10.25 | 42.2 | 36.5 | 39.35 | 7.7 | 10.3 | 9 | 82.1 | 97.9 | 81.2 |
| 57 | Barefoot | 28 | 162 | 60 | 22.9 | 36 | 144.4 | 1.46 | 92.9 | 0.99 | 0.85 | 0.92 | 1.31 | 1.25 | 1.28 | 80.5 | 76.7 | 78.6 | 69.1 | 58.9 | 64 | 30.9 | 41.1 | 36 | 11.5 | 17 | 14.25 | 44.2 | 29.5 | 36.85 | 7.9 | 9.4 | 8.65 | 76.8 | 87.2 | 99.1 |
| 58 | Barefoot | 28 | 163 | 52 | 15.8 | 37 | 131.5 | 1.44 | 89.5 | 0.92 | 0.92 | 0.92 | 1.31 | 1.33 | 1.32 | 80.2 | 81 | 80.6 | 61.5 | 59.5 | 60.5 | 38.5 | 40.5 | 39.5 | 12.3 | 8.9 | 10.6 | 40.6 | 38.3 | 39.45 | 12 | 13.4 | 12.7 | 53.7 | 96.5 | 98.1 |
| 59 | Barefoot | 28 | 168 | 55 | 19.5 | 36 | 120.8 | 1.41 | 96 | 1.01 | 0.99 | 1 | 1.4 | 1.41 | 1.405 | 84.8 | 85.2 | 85 | 63.2 | 62.4 | 62.8 | 36.8 | 37.6 | 37.2 | 11.2 | 12.6 | 11.9 | 38.9 | 37.4 | 38.15 | 10.1 | 11.8 | 10.95 | 83 | 99.2 | 98.4 |
| 60 | Barefoot | 28 | 172 | 65 | 22 | 38 | 105.2 | 1.57 | 95.5 | 1.18 | 1.14 | 1.16 | 1.9 | 1.74 | 1.82 | 109.6 | 100.3 | 104.95 | 64.7 | 60.9 | 62.8 | 35.3 | 39.1 | 37.2 | 11.4 | 12 | 11.7 | 41.3 | 36 | 38.65 | 7 | 8.5 | 7.75 | 80.5 | 97.3 | 98.4 |
| 61 | Barefoot | 28 | 176 | 66 | 21.3 | 37 | 105 | 1.85 | 95.8 | 1.13 | 1.15 | 1.14 | 2.32 | 1.93 | 2.125 | 134.4 | 111.4 | 122.9 | 63.7 | 61.3 | 62.5 | 36.3 | 38.7 | 37.5 | 13 | 13.8 | 13.4 | 36.4 | 35.8 | 36.1 | 7.3 | 8.4 | 7.85 | 87.2 | 98.6 | 99.2 |
| 62 | Barefoot | 28 | 177 | 81 | 25.9 | 39 | 98.7 | 1.18 | 92.7 | 1.35 | 1.25 | 1.3 | 1.63 | 1.41 | 1.52 | 93 | 80.7 | 86.85 | 61.6 | 58.7 | 60.15 | 38.4 | 41.3 | 39.85 | 8.5 | 9.3 | 8.9 | 43.8 | 40.7 | 42.25 | 5.2 | 5 | 5.1 | 21.7 | 94.8 | 90.2 |
| 63 | Barefoot | 29 | 162 | 45 | 17.1 | 36 | 114 | 1.2 | 96.5 | 1.06 | 1.05 | 1.055 | 1.27 | 1.27 | 1.27 | 77.7 | 77.7 | 77.7 | 60.6 | 62.9 | 61.75 | 39.4 | 37.1 | 38.25 | 10.8 | 13.7 | 12.25 | 36.8 | 38 | 37.4 | 8.1 | 8.1 | 8.1 | 42 | 97.7 | 97.4 |
| 64 | Barefoot | 29 | 162 | 46 | 17.5 | 36 | 114.4 | 1.26 | 98.2 | 1.05 | 1.04 | 1.045 | 1.32 | 1.31 | 1.315 | 80.8 | 80.5 | 80.65 | 60 | 58.1 | 59.05 | 40 | 41.9 | 40.95 | 8.6 | 8.9 | 8.75 | 41.4 | 40.5 | 40.95 | 7.6 | 9 | 8.3 | 64.8 | 98.7 | 94.7 |
| 65 | Barefoot | 29 | 165 | 59 | 21.7 | 36 | 113.6 | 1.31 | 96.2 | 1.05 | 1.06 | 1.055 | 1.35 | 1.4 | 1.375 | 81.6 | 84.5 | 83.05 | 64.1 | 62.9 | 63.5 | 35.9 | 37.1 | 36.5 | 11.5 | 14.8 | 13.15 | 36.7 | 36.2 | 36.45 | 6.7 | 5.8 | 6.25 | 79.8 | 97.1 | 98.5 |
| 66 | Barefoot | 29 | 165 | 65 | 23.9 | 38 | 103.9 | 0.93 | 95.7 | 1.17 | 1.16 | 1.165 | 1.09 | 1.08 | 1.085 | 67 | 66 | 66.5 | 63.2 | 60.6 | 61.9 | 36.8 | 39.4 | 38.1 | 11.5 | 11.7 | 11.6 | 40.2 | 37.2 | 38.7 | 8.5 | 8.1 | 8.3 | 52.7 | 98.2 | 97.7 |
| 67 | Barefoot | 29 | 180 | 59 | 18.2 | 40 | 110.3 | 1.07 | 95.2 | 1.1 | 1.09 | 1.095 | 1.17 | 1.17 | 1.17 | 64.9 | 64.8 | 64.85 | 64.2 | 56.9 | 60.55 | 35.8 | 43.1 | 39.45 | 8.9 | 13.8 | 11.35 | 41.6 | 36.1 | 38.85 | 5 | 7.2 | 6.1 | 62.3 | 97.9 | 98.9 |
| 68 | Barefoot | 29 | 183 | 64 | 19.1 | 40 | 115.5 | 1.27 | 89.4 | 1.04 | 1.03 | 1.035 | 1.29 | 1.34 | 1.315 | 71.9 | 74.5 | 73.2 | 63.2 | 54.6 | 58.9 | 36.8 | 45.4 | 41.1 | 6.7 | 9.7 | 8.2 | 44.9 | 38.1 | 41.5 | 6.3 | 5.8 | 6.05 | 89.5 | 97.2 | 97.4 |
| 69 | Barefoot | 30 | 161 | 62 | 23.9 | 36 | 127.8 | 1.41 | 92.5 | 0.95 | 0.94 | 0.945 | 1.33 | 1.33 | 1.33 | 82.9 | 82.7 | 82.8 | 67.8 | 58.6 | 63.2 | 32.2 | 41.4 | 36.8 | 13 | 15.9 | 14.45 | 39.1 | 31.4 | 35.25 | 8.9 | 9.3 | 9.1 | 68.3 | 99 | 97.4 |
| 70 | Barefoot | 30 | 164 | 63 | 23.4 | 38 | 104.5 | 1.08 | 90.1 | 1.16 | 1.15 | 1.155 | 1.19 | 1.31 | 1.25 | 71.5 | 79 | 75.25 | 64.5 | 61.7 | 63.1 | 35.5 | 38.3 | 36.9 | 13.6 | 11.1 | 12.35 | 38.3 | 36.9 | 37.6 | 5.2 | 5.6 | 5.4 | 60.5 | 97.3 | 98.2 |
| 71 | Barefoot | 30 | 175 | 82 | 26.8 | 40 | 99 | 1.25 | 95.5 | 1.21 | 1.21 | 1.21 | 1.66 | 1.38 | 1.52 | 94.9 | 78.8 | 86.85 | 60.7 | 59.6 | 60.15 | 39.3 | 40.4 | 39.85 | 10.1 | 10.9 | 10.5 | 39.6 | 39.2 | 39.4 | 6.8 | 8.1 | 7.45 | 27.5 | 97.3 | 90.7 |
| 72 | Barefoot | 31 | 169 | 61 | 21.4 | 37 | 115.6 | 1.35 | 95.3 | 1.04 | 1.16 | 1.1 | 1.47 | 1.47 | 1.47 | 88.8 | 88.8 | 88.8 | 61.3 | 64.2 | 62.75 | 38.7 | 35.8 | 37.25 | 9.8 | 12 | 10.9 | 38.3 | 42.2 | 40.25 | 5.6 | 6 | 5.8 | 59.9 | 98.8 | 98.8 |
| 73 | Barefoot | 31 | 171 | 70 | 23.9 | 39 | 101.5 | 1.7 | 97.9 | 1.2 | 1.2 | 1.2 | 2.27 | 1.82 | 2.045 | 131.2 | 105.1 | 118.15 | 63.8 | 61.2 | 62.5 | 36.2 | 38.8 | 37.5 | 11.3 | 13 | 12.15 | 39.2 | 37.6 | 38.4 | 7.8 | 8.2 | 8 | 87.8 | 98.6 | 99.3 |
| 74 | Barefoot | 32 | 165 | 50 | 18.4 | 37 | 114.1 | 1.22 | 97.6 | 1.05 | 1.04 | 1.045 | 1.27 | 1.28 | 1.275 | 77.7 | 78.4 | 78.05 | 58.9 | 59.5 | 59.2 | 41.1 | 40.5 | 40.8 | 9.5 | 9.1 | 9.3 | 40.2 | 40.8 | 40.5 | 7.2 | 8.6 | 7.9 | 58.1 | 97.7 | 95.7 |
| 75 | Barefoot | 32 | 177 | 60 | 19.2 | 39 | 114.5 | 1.26 | 96.4 | 1.05 | 1.05 | 1.05 | 1.32 | 1.32 | 1.32 | 73.2 | 73.1 | 73.15 | 64.6 | 58.8 | 61.7 | 35.4 | 41.2 | 38.3 | 9.8 | 13.8 | 11.8 | 40.8 | 35.7 | 38.25 | 6.9 | 6.9 | 6.9 | 77.3 | 98.1 | 98.7 |
| 1 | Ballerina | 18 | 162 | 57 | 21.7 | 39 | 123.3 | 1.58 | 96.2 | 0.98 | 0.97 | 0.975 | 1.55 | 1.53 | 1.54 | 94.4 | 93.4 | 93.9 | 60 | 57.8 | 58.9 | 40 | 42.2 | 41.1 | 9.5 | 9.1 | 9.3 | 41.1 | 40.2 | 40.65 | 7.1 | 6.7 | 6.9 | 90.6 | 94.3 | 86.9 |
| 2 | Ballerina | 19 | 161 | 52 | 20.1 | 38 | 122 | 2.82 | 92.8 | 0.98 | 0.98 | 0.98 | 2.8 | 2.7 | 2.75 | 170.8 | 164.6 | 167.7 | 61.6 | 57.7 | 59.65 | 38.4 | 42.3 | 40.35 | 7.8 | 11.7 | 9.75 | 41.8 | 38.3 | 40.05 | 7.5 | 6.7 | 7.1 | 64 | 95.8 | 96.2 |
| 3 | Ballerina | 19 | 165 | 53 | 19.5 | 39 | 124.6 | 2.54 | 92.1 | 0.97 | 0.96 | 0.965 | 2.71 | 2.2 | 2.455 | 165.5 | 134 | 149.75 | 60.8 | 58.8 | 59.8 | 39.2 | 41.2 | 40.2 | 8.2 | 11.4 | 9.8 | 41.2 | 39.4 | 40.3 | 7.4 | 6.4 | 6.9 | 86.5 | 93.8 | 91.4 |
| 4 | Ballerina | 19 | 168 | 48 | 17 | 37 | 123.4 | 2.49 | 96.2 | 0.97 | 0.98 | 0.975 | 2.19 | 2.67 | 2.43 | 132.5 | 161.7 | 147.1 | 57.2 | 59 | 58.1 | 42.8 | 41 | 41.9 | 7.9 | 8.9 | 8.4 | 41.1 | 42.3 | 41.7 | 9.3 | 9.2 | 9.25 | 85 | 97.9 | 96 |
| 5 | Ballerina | 19 | 169 | 61 | 21.4 | 38 | 122.7 | 2.54 | 88.4 | 0.98 | 1.01 | 0.995 | 2.3 | 2.8 | 2.55 | 139.1 | 170 | 154.55 | 58.8 | 60.9 | 59.85 | 41.2 | 39.1 | 40.15 | 8.2 | 13.5 | 10.85 | 40.1 | 39.4 | 39.75 | 9.4 | 9.8 | 9.6 | 48.7 | 97.5 | 91.3 |
| 6 | Ballerina | 19 | 172 | 59 | 19.9 | 39 | 138.3 | 2.28 | 93.7 | 0.94 | 1.02 | 0.98 | 2.29 | 2.09 | 2.19 | 138.9 | 126.6 | 132.75 | 54.5 | 66 | 60.25 | 45.5 | 34 | 39.75 | 7.4 | 9.1 | 8.25 | 36.7 | 50.3 | 43.5 | 7 | 6 | 6.5 | 45.4 | 89.9 | 85.4 |
| 7 | Ballerina | 19 | 172 | 69 | 23.3 | 40 | 120.1 | 1.31 | 97.7 | 1.01 | 1 | 1.005 | 1.33 | 1.3 | 1.315 | 83.2 | 81.2 | 82.2 | 58.9 | 57.7 | 58.3 | 41.1 | 42.3 | 41.7 | 8.6 | 8 | 8.3 | 42.4 | 41.3 | 41.85 | 10.6 | 9.3 | 9.95 | 29.9 | 98.6 | 96.6 |
| 8 | Ballerina | 19 | 173 | 67 | 22.4 | 38 | 118.2 | 1.44 | 90.7 | 1.03 | 1.02 | 1.025 | 1.48 | 1.46 | 1.47 | 92.2 | 91.1 | 91.65 | 59.2 | 55.3 | 57.25 | 40.8 | 44.7 | 42.75 | 9.5 | 9.1 | 9.3 | 41.5 | 37.8 | 39.65 | 6.9 | 8.5 | 7.7 | 45.2 | 95.8 | 96 |
| 9 | Ballerina | 20 | 159 | 77 | 30.5 | 37 | 125.5 | 1.5 | 97.5 | 0.96 | 1 | 0.98 | 1.36 | 1.6 | 1.48 | 85 | 99.8 | 92.4 | 59.2 | 60.5 | 59.85 | 40.8 | 39.5 | 40.15 | 9.4 | 8.3 | 8.85 | 41.1 | 43.1 | 42.1 | 9.9 | 7.6 | 8.75 | 34.3 | 98.5 | 97.3 |
| 10 | Ballerina | 20 | 163 | 61 | 23 | 39 | 123.8 | 1.36 | 93 | 1.01 | 1.01 | 1.01 | 1.35 | 1.38 | 1.365 | 83.6 | 85 | 84.3 | 57 | 57.9 | 57.45 | 43 | 42.1 | 42.55 | 10.5 | 5 | 7.75 | 41.5 | 46 | 43.75 | 5.3 | 5.5 | 5.4 | 91.4 | 98.8 | 98.7 |
| 11 | Ballerina | 20 | 164 | 76 | 28.3 | 39 | 119.7 | 1.38 | 96.2 | 1 | 1.01 | 1.005 | 1.39 | 1.39 | 1.39 | 85.8 | 85.8 | 85.8 | 57.7 | 57.2 | 57.45 | 42.3 | 42.8 | 42.55 | 8.9 | 9.9 | 9.4 | 40.3 | 41.4 | 40.85 | 6 | 6.7 | 6.35 | 76.4 | 98.6 | 98.7 |
| 12 | Ballerina | 20 | 167 | 58 | 20.8 | 37 | 117.8 | 1.28 | 91.8 | 1.03 | 1.04 | 1.035 | 1.32 | 1.32 | 1.32 | 81.6 | 81.2 | 81.4 | 59.3 | 61.2 | 60.25 | 40.7 | 38.8 | 39.75 | 8.3 | 16.1 | 12.2 | 38.6 | 38.9 | 38.75 | 6.8 | 4.9 | 5.85 | 61.3 | 95.9 | 96.8 |
| 13 | Ballerina | 20 | 169 | 65 | 22.8 | 37 | 122.1 | 1.71 | 95 | 0.99 | 0.99 | 0.99 | 1.69 | 1.68 | 1.685 | 103.2 | 102.3 | 102.75 | 61.9 | 59.9 | 60.9 | 38.1 | 40.1 | 39.1 | 11.2 | 11.8 | 11.5 | 40.1 | 37.7 | 38.9 | 11.1 | 12.4 | 11.75 | 81.5 | 97.1 | 97.1 |
| 14 | Ballerina | 21 | 158 | 57 | 22.8 | 36 | 125.1 | 1.77 | 98.5 | 0.97 | 0.96 | 0.965 | 1.73 | 1.68 | 1.705 | 105.7 | 102.3 | 104 | 61 | 60.4 | 60.7 | 39 | 39.6 | 39.3 | 10.7 | 11.1 | 10.9 | 39.3 | 39.2 | 39.25 | 11.1 | 12.1 | 11.6 | 89.6 | 98.2 | 97.4 |
| 15 | Ballerina | 21 | 163 | 57 | 21.5 | 38 | 135.3 | 1.75 | 97.2 | 1 | 0.99 | 0.995 | 1.62 | 1.67 | 1.645 | 98.6 | 101.7 | 100.15 | 60.4 | 70.2 | 65.3 | 39.6 | 29.8 | 34.7 | 13.4 | 18.3 | 15.85 | 32.2 | 38.6 | 35.4 | 9.6 | 7.7 | 8.65 | 90.1 | 95.6 | 75.8 |
| 16 | Ballerina | 21 | 165 | 60 | 22 | 37 | 99.2 | 1.31 | 97 | 1.33 | 1.25 | 1.29 | 1.54 | 1.74 | 1.64 | 93 | 105 | 99 | 59.3 | 62.9 | 61.1 | 40.7 | 37.1 | 38.9 | 11.2 | 8.7 | 9.95 | 39.6 | 43.5 | 41.55 | 4.7 | 5.6 | 5.15 | 80.6 | 97.8 | 94.9 |
| 17 | Ballerina | 21 | 165 | 63 | 23.1 | 38 | 101.2 | 1.5 | 98.1 | 1.2 | 1.18 | 1.19 | 1.87 | 1.65 | 1.76 | 112.4 | 99.6 | 106 | 63.4 | 59 | 61.2 | 36.6 | 41 | 38.8 | 8.8 | 13.5 | 11.15 | 39.6 | 37.4 | 38.5 | 5.5 | 6 | 5.75 | 53.3 | 98.4 | 83.8 |
| 18 | Ballerina | 21 | 172 | 73 | 24.7 | 40 | 100 | 1.35 | 95.7 | 1.24 | 1.33 | 1.285 | 1.71 | 1.81 | 1.76 | 102.8 | 108.7 | 105.75 | 59.7 | 63 | 61.35 | 40.3 | 37 | 38.65 | 10 | 11.7 | 10.85 | 39.1 | 42.9 | 41 | 6.7 | 6 | 6.35 | 84.1 | 98.4 | 87.9 |
| 19 | Ballerina | 21 | 176 | 62 | 20 | 37 | 117.7 | 1.38 | 90.7 | 1.03 | 1.01 | 1.02 | 1.43 | 1.39 | 1.41 | 84.3 | 81.7 | 83 | 62.5 | 64.4 | 63.45 | 37.5 | 35.6 | 36.55 | 10 | 14.8 | 12.4 | 34.9 | 39.3 | 37.1 | 10.6 | 8.9 | 9.75 | 62.4 | 93.8 | 96 |
| 20 | Ballerina | 21 | 177 | 65 | 20.7 | 39 | 91.5 | 1.17 | 74.9 | 1.41 | 1.41 | 1.41 | 1.47 | 1.63 | 1.55 | 86.3 | 95.9 | 91.1 | 64.7 | 52.6 | 58.65 | 35.3 | 47.4 | 41.35 | 6.9 | 10.2 | 8.55 | 47.4 | 35.2 | 41.3 | 8 | 8.8 | 8.4 | 46.3 | 91.5 | 89.9 |
| 21 | Ballerina | 22 | 157 | 51 | 20.7 | 36 | 95.5 | 1.33 | 79.8 | 1.4 | 1.38 | 1.39 | 1.83 | 1.86 | 1.845 | 107.9 | 109.2 | 108.55 | 61.5 | 62 | 61.75 | 38.5 | 38 | 38.25 | 10.1 | 12.6 | 11.35 | 38.5 | 38.4 | 38.45 | 7.9 | 6.3 | 7.1 | 55.7 | 80.6 | 69.1 |
| 22 | Ballerina | 22 | 162 | 52 | 19.8 | 38 | 123.6 | 1.69 | 95.8 | 0.97 | 0.99 | 0.98 | 1.64 | 1.67 | 1.655 | 96.8 | 98.3 | 97.55 | 58.4 | 59.4 | 58.9 | 41.6 | 40.6 | 41.1 | 9.3 | 10.1 | 9.7 | 40.4 | 40.7 | 40.55 | 11.1 | 9.9 | 10.5 | 58 | 99.1 | 78.2 |

| IDENTIFICATION | | DEMOGRAPHICS | | | | | GENERAL GAIT | | | Stride Duration (s) | | | Stride Length (m) | | | % Stride Length (% height) | | | Stance Duration (%) | | | Swing Duration (%) | | | Double Support (%) | | | Single Support (%) | | | Propulsion Index | | | Pelvis Symmetry (Symmetry Index) | | |
|---|---|---|---|---|---|---|---|---|---|---|---|---|---|---|---|---|---|---|---|---|---|---|---|---|---|---|---|---|---|---|---|---|---|---|---|---|
| ID | Condition | Age (yr) | Height (cm) | Weight (kg) | BMI (kg/m$^2$) | Shoe Size (EU) | Cadence (steps/min) | Speed (m/s) | Symmetry Index | Left | Right | Avg | Left | Right | Avg | Left | Right | Avg | Left | Right | Avg | Left | Right | Avg | First Left | First Right | Avg First | Left | Right | Avg | Left | Right | Avg | Tilt | Obliquity | Rotation |
| 23 | Ballerina | 22 | 162 | 53 | 20.2 | 39 | 112.9 | 1.79 | 93.7 | 1.09 | 1.1 | 1.095 | 1.87 | 1.94 | 1.905 | 110.2 | 114.2 | 112.2 | 58.8 | 56 | 57.4 | 41.2 | 44 | 42.6 | 6.9 | 8.7 | 7.8 | 43.1 | 40.5 | 41.8 | 8.1 | 8.5 | 8.3 | 59.6 | 95.9 | 83.7 |
| 24 | Ballerina | 22 | 162 | 56 | 21.3 | 37 | 126.2 | 1.6 | 92.4 | 0.97 | 1.03 | 1 | 1.62 | 1.54 | 1.58 | 95.4 | 90.6 | 93 | 69 | 63.3 | 66.15 | 31 | 36.7 | 33.85 | 18.6 | 14.6 | 16.6 | 36.1 | 30.3 | 33.2 | 5.2 | 7.1 | 6.15 | 78.5 | 88.8 | 77.3 |
| 25 | Ballerina | 22 | 168 | 63 | 22.3 | 38 | 127 | 1.75 | 96.8 | 0.97 | 0.95 | 0.96 | 1.67 | 1.67 | 1.67 | 96.7 | 96.7 | 96.7 | 61.6 | 61.9 | 61.75 | 38.4 | 38.1 | 38.25 | 11.4 | 11.6 | 11.5 | 38.8 | 39.2 | 39 | 11.1 | 10 | 10.55 | 86.8 | 99 | 94.7 |
| 26 | Ballerina | 22 | 168 | 63 | 22.3 | 39 | 127 | 1.8 | 96.9 | 0.96 | 0.95 | 0.955 | 1.72 | 1.7 | 1.71 | 99.6 | 98 | 98.8 | 63 | 61.9 | 62.45 | 37 | 38.1 | 37.55 | 12.5 | 12.2 | 12.35 | 38.5 | 37.6 | 38.05 | 10.1 | 8.8 | 9.45 | 94.2 | 99 | 88.5 |
| 27 | Ballerina | 22 | 170 | 64 | 22.1 | 37 | 126.4 | 2.05 | 95.3 | 0.96 | 0.95 | 0.955 | 1.92 | 1.98 | 1.95 | 111.2 | 114.7 | 112.95 | 62.8 | 61.1 | 61.95 | 37.2 | 38.9 | 38.05 | 12.1 | 12.6 | 12.35 | 38.2 | 37.4 | 37.8 | 11 | 9.1 | 10.05 | 79.8 | 98.6 | 97.1 |
| 28 | Ballerina | 22 | 173 | 59 | 19.7 | 37 | 102.8 | 1.41 | 89 | 1.15 | 1.18 | 1.165 | 1.66 | 1.61 | 1.635 | 95.1 | 92.1 | 93.6 | 57.4 | 62.8 | 60.1 | 42.6 | 37.2 | 39.9 | 10.5 | 10.8 | 10.65 | 35.7 | 41.9 | 38.8 | 8.5 | 9.5 | 9 | 50.5 | 96.9 | 93.9 |
| 29 | Ballerina | 23 | 163 | 81 | 30.5 | 38 | 107.6 | 1.49 | 96.7 | 1.12 | 1.12 | 1.12 | 1.69 | 1.64 | 1.665 | 96.5 | 93.9 | 95.2 | 59.8 | 63.7 | 61.75 | 40.2 | 36.3 | 38.25 | 13.8 | 9.4 | 11.6 | 36.6 | 40.4 | 38.5 | 9 | 8.8 | 8.9 | 62.4 | 98.8 | 95.7 |
| 30 | Ballerina | 23 | 164 | 48 | 17.8 | 38 | 118.3 | 1.54 | 90.7 | 1.09 | 1.03 | 1.06 | 1.62 | 1.62 | 1.62 | 92.7 | 92.6 | 92.65 | 63.8 | 54.9 | 59.35 | 36.2 | 45.1 | 40.65 | 6.9 | 9.6 | 8.25 | 47.8 | 38.5 | 43.15 | 6.8 | 8.5 | 7.65 | 54.9 | 96.7 | 85.7 |
| 31 | Ballerina | 23 | 166 | 60 | 21.8 | 38 | 130.2 | 1.64 | 97.3 | 0.91 | 0.92 | 0.915 | 1.48 | 1.53 | 1.505 | 92.2 | 95.4 | 93.8 | 58.1 | 59.3 | 58.7 | 41.9 | 40.7 | 41.3 | 9.6 | 9.1 | 9.35 | 39.7 | 40.7 | 40.2 | 11.5 | 10.1 | 10.8 | 21.3 | 99.2 | 96.9 |
| 32 | Ballerina | 23 | 167 | 50 | 17.9 | 37 | 136.8 | 1.72 | 94.6 | 0.88 | 0.89 | 0.885 | 1.53 | 1.51 | 1.52 | 95.6 | 94.5 | 95.05 | 60.9 | 59.3 | 60.1 | 39.1 | 40.7 | 39.9 | 10.7 | 8.9 | 9.8 | 41.1 | 39.5 | 40.3 | 11.5 | 11.5 | 11.5 | 44.2 | 98.8 | 95.9 |
| 33 | Ballerina | 23 | 172 | 57 | 19.3 | 39 | 139 | 1.92 | 82.6 | 0.85 | 0.85 | 0.85 | 1.62 | 1.63 | 1.625 | 101.1 | 101.9 | 101.5 | 63.7 | 58.6 | 61.15 | 36.3 | 41.4 | 38.85 | 13.7 | 9.8 | 11.75 | 40.6 | 34.5 | 37.55 | 11.8 | 12.3 | 12.05 | 34.4 | 96.8 | 49.9 |
| 34 | Ballerina | 23 | 175 | 53 | 17.3 | 38 | 114.7 | 1.08 | 95.7 | 1.05 | 1.04 | 1.045 | 1.11 | 1.15 | 1.13 | 63.3 | 65.5 | 64.4 | 59.4 | 57.7 | 58.55 | 40.6 | 42.3 | 41.45 | 10.1 | 7.7 | 8.9 | 41.9 | 39.6 | 40.75 | 7.9 | 7.5 | 7.7 | 67.6 | 97.2 | 97.7 |
| 35 | Ballerina | 23 | 175 | 63 | 20.6 | 37 | 117.8 | 1.06 | 94 | 1.04 | 1.05 | 1.045 | 1.09 | 1.11 | 1.1 | 62.4 | 63.3 | 62.85 | 60.3 | 57.8 | 59.05 | 39.7 | 42.2 | 40.95 | 9.6 | 7.2 | 8.4 | 43.3 | 40.6 | 41.95 | 7.9 | 6.5 | 7.2 | 78 | 93.5 | 95.7 |
| 36 | Ballerina | 23 | 175 | 68 | 22.2 | 39 | 116.1 | 1.11 | 95 | 1.05 | 1.05 | 1.05 | 1.18 | 1.15 | 1.165 | 67.4 | 65.6 | 66.5 | 59.6 | 57.3 | 58.45 | 40.4 | 42.7 | 41.55 | 9.8 | 6.7 | 8.25 | 42.9 | 39.9 | 41.4 | 8.3 | 7.6 | 7.95 | 88.5 | 97.7 | 98.6 |
| 37 | Ballerina | 24 | 159 | 56 | 22.2 | 36 | 119.6 | 1.41 | 98.2 | 1.01 | 1 | 1.005 | 1.4 | 1.41 | 1.405 | 85.7 | 86.2 | 85.95 | 61.9 | 58.3 | 60.1 | 38.1 | 41.7 | 39.9 | 7.9 | 10.5 | 9.2 | 42.1 | 39.2 | 40.65 | 9.3 | 8.7 | 9 | 97.3 | 98.7 | 70.7 |
| 38 | Ballerina | 24 | 163 | 61 | 23 | 37 | 120.7 | 1.41 | 97.9 | 0.99 | 0.99 | 0.99 | 1.4 | 1.39 | 1.395 | 85.4 | 84.9 | 85.15 | 58.2 | 57.9 | 58.05 | 41.8 | 42.1 | 41.95 | 8.4 | 8.1 | 8.25 | 42.1 | 41.3 | 41.7 | 11.2 | 9.8 | 10.5 | 98.5 | 97.5 | 96.8 |
| 39 | Ballerina | 24 | 166 | 51 | 18.5 | 36 | 125.2 | 1.5 | 98.6 | 0.96 | 0.97 | 0.965 | 1.45 | 1.44 | 1.445 | 88.3 | 87.8 | 88.05 | 58 | 57.9 | 57.95 | 42 | 42.1 | 42.05 | 8.3 | 7.6 | 7.95 | 42.4 | 42.1 | 42.25 | 10.7 | 9.8 | 10.25 | 94.5 | 98.3 | 98.8 |
| 40 | Ballerina | 24 | 167 | 46 | 16.5 | 36 | 123.3 | 1.4 | 94.6 | 0.98 | 0.97 | 0.975 | 1.36 | 1.37 | 1.365 | 80.2 | 80.3 | 80.25 | 58.6 | 58.6 | 58.6 | 41.4 | 41.4 | 41.4 | 9.4 | 7.2 | 8.3 | 41.7 | 41.4 | 41.55 | 8.7 | 6.6 | 7.65 | 43.1 | 98.1 | 99.3 |
| 41 | Ballerina | 24 | 171 | 55 | 18.8 | 38 | 119.8 | 1.3 | 92.6 | 1.01 | 1.01 | 1.01 | 1.31 | 1.31 | 1.31 | 76.9 | 77.2 | 77.05 | 59.2 | 56.3 | 57.75 | 40.8 | 43.7 | 42.25 | 7.7 | 7.9 | 7.8 | 43.8 | 40.7 | 42.25 | 9.7 | 8.7 | 9.2 | 32.3 | 97.7 | 85.4 |
| 42 | Ballerina | 24 | 172 | 61 | 20.6 | 38 | 123.6 | 1.31 | 96.5 | 0.99 | 0.97 | 0.98 | 1.3 | 1.27 | 1.285 | 76.7 | 74.5 | 75.6 | 58.6 | 59.1 | 58.85 | 41.4 | 40.9 | 41.15 | 9.1 | 8 | 8.55 | 41.6 | 41.9 | 41.75 | 9.6 | 7.4 | 8.5 | 66.5 | 98.7 | 96.6 |
| 43 | Ballerina | 24 | 177 | 66 | 21.1 | 39 | 118.5 | 1.34 | 94 | 1.01 | 1 | 1.005 | 1.35 | 1.34 | 1.345 | 78.3 | 77.5 | 77.9 | 61.4 | 61.2 | 61.3 | 38.6 | 38.8 | 38.7 | 9.4 | 12.2 | 10.8 | 39.1 | 38.9 | 39 | 9 | 9.2 | 9.1 | 95.9 | 98.8 | 98.9 |
| 44 | Ballerina | 25 | 162 | 54 | 20.6 | 37 | 115.2 | 1.3 | 94.7 | 1.04 | 1.03 | 1.035 | 1.35 | 1.34 | 1.345 | 78.1 | 77.6 | 77.85 | 62.8 | 60.7 | 61.75 | 37.2 | 39.3 | 38.25 | 8.9 | 14.2 | 11.55 | 39.7 | 37.1 | 38.4 | 11 | 10.5 | 10.75 | 90 | 98.4 | 97.6 |
| 45 | Ballerina | 25 | 169 | 59 | 20.7 | 38 | 115.8 | 1.26 | 96.4 | 1.03 | 1.04 | 1.035 | 1.32 | 1.29 | 1.305 | 76.5 | 74.5 | 75.5 | 63.4 | 60.9 | 62.15 | 36.6 | 39.1 | 37.85 | 8.9 | 14.2 | 11.55 | 40.1 | 36.4 | 38.25 | 9.4 | 9.8 | 9.6 | 92.2 | 99.3 | 98.9 |
| 46 | Ballerina | 25 | 172 | 54 | 18.3 | 36 | 131.2 | 1.57 | 85.6 | 0.92 | 0.92 | 0.92 | 1.43 | 1.45 | 1.44 | 87.5 | 88.3 | 87.9 | 60.5 | 55.8 | 58.15 | 39.5 | 44.2 | 41.85 | 8.5 | 7.6 | 8.05 | 44.1 | 39.8 | 41.95 | 11.7 | 15.5 | 13.6 | 66.9 | 98 | 97.6 |
| 47 | Ballerina | 26 | 162 | 47 | 17.9 | 36 | 128.2 | 1.53 | 79.4 | 0.94 | 0.93 | 0.935 | 1.44 | 1.41 | 1.425 | 87.8 | 86.2 | 87 | 61.2 | 55.7 | 58.45 | 38.8 | 44.3 | 41.55 | 9.6 | 8.6 | 9.1 | 43.6 | 37.3 | 40.45 | 11.6 | 16.7 | 14.15 | 38.6 | 97.4 | 96.7 |
| 48 | Ballerina | 26 | 164 | 50 | 18.6 | 37 | 126.3 | 1.54 | 83.4 | 0.95 | 0.95 | 0.95 | 1.45 | 1.46 | 1.455 | 88.3 | 89 | 88.65 | 60.9 | 56.1 | 58.5 | 39.1 | 43.9 | 41.5 | 9 | 7.6 | 8.3 | 44.2 | 39 | 41.6 | 11.9 | 15.4 | 13.65 | 74.8 | 95.5 | 97.9 |
| 49 | Ballerina | 26 | 169 | 58 | 20.3 | 37 | 119.6 | 1.3 | 98.8 | 1 | 1.01 | 1.005 | 1.31 | 1.3 | 1.305 | 76.3 | 76 | 76.15 | 59.1 | 58.2 | 58.65 | 40.9 | 41.8 | 41.35 | 9 | 9.2 | 9.1 | 40.7 | 40.6 | 40.65 | 7.8 | 9.5 | 8.65 | 81.9 | 97.8 | 68.1 |
| 50 | Ballerina | 26 | 172 | 55 | 18.6 | 37 | 121.5 | 1.38 | 98 | 0.99 | 0.98 | 0.985 | 1.35 | 1.38 | 1.365 | 78.8 | 80.9 | 79.85 | 59.7 | 57.6 | 58.65 | 40.3 | 42.4 | 41.35 | 8.3 | 8.8 | 8.55 | 42.1 | 40.7 | 41.4 | 8.5 | 9.2 | 8.85 | 93.1 | 99.2 | 97.3 |
| 51 | Ballerina | 26 | 173 | 77 | 25.7 | 40 | 126.5 | 1.53 | 98.3 | 0.97 | 0.94 | 0.955 | 1.46 | 1.48 | 1.47 | 85.1 | 86.7 | 85.9 | 59.7 | 58.3 | 59 | 40.3 | 41.7 | 41 | 9.7 | 9.2 | 9.45 | 41 | 41 | 41 | 8.3 | 9.7 | 9 | 81 | 99.2 | 99 |
| 52 | Ballerina | 27 | 158 | 61 | 24.4 | 37 | 146.3 | 1.63 | 82.4 | 0.88 | 0.89 | 0.885 | 1.38 | 1.39 | 1.385 | 83.8 | 84 | 83.9 | 66.7 | 68.3 | 67.5 | 33.3 | 31.7 | 32.5 | 21.2 | 14.6 | 17.9 | 31 | 32.7 | 31.85 | 13.5 | 12.7 | 13.1 | 41.2 | 97 | 95.7 |
| 53 | Ballerina | 27 | 162 | 64 | 24.4 | 37 | 121.5 | 1.44 | 97 | 1 | 0.99 | 0.995 | 1.43 | 1.44 | 1.435 | 86.4 | 87 | 86.7 | 65.3 | 65.7 | 65.5 | 34.7 | 34.3 | 34.5 | 15 | 14.5 | 14.75 | 36 | 35 | 35.5 | 11.3 | 10.2 | 10.75 | 30.1 | 98.8 | 98.3 |
| 54 | Ballerina | 27 | 163 | 51 | 19.2 | 36 | 146.5 | 1.67 | 90.1 | 0.88 | 0.9 | 0.89 | 1.42 | 1.43 | 1.425 | 85.9 | 86.8 | 86.35 | 62.8 | 68.1 | 65.45 | 37.2 | 31.9 | 34.55 | 18.3 | 14 | 16.15 | 30.2 | 36.9 | 33.55 | 13.3 | 11.7 | 12.5 | 93.3 | 90.5 | 92.3 |
| 55 | Ballerina | 27 | 164 | 66 | 24.5 | 36 | 122.1 | 1.45 | 97.2 | 1 | 0.98 | 0.99 | 1.43 | 1.43 | 1.43 | 88.8 | 89.1 | 88.95 | 57.9 | 57.6 | 57.75 | 42.1 | 42.4 | 42.25 | 9.6 | 7.5 | 8.55 | 40.9 | 42.7 | 41.8 | 9.6 | 8.6 | 9.1 | 40.3 | 99.4 | 99.4 |
| 56 | Ballerina | 27 | 172 | 57 | 19.3 | 39 | 129.5 | 1.61 | 97.8 | 0.93 | 0.93 | 0.93 | 1.48 | 1.5 | 1.49 | 92.2 | 93.2 | 92.7 | 57.7 | 57.6 | 57.65 | 42.3 | 42.4 | 42.35 | 9.6 | 7.9 | 8.75 | 40.2 | 42.4 | 41.3 | 10.6 | 9.7 | 10.15 | 66.7 | 99.4 | 98.5 |
| 57 | Ballerina | 28 | 162 | 60 | 22.9 | 36 | 130.4 | 1.52 | 89.6 | 0.96 | 0.94 | 0.95 | 1.44 | 1.45 | 1.445 | 89.4 | 90 | 89.7 | 66.9 | 61 | 63.95 | 33.1 | 39 | 36.05 | 16 | 13.5 | 14.75 | 37.9 | 34.4 | 36.15 | 9.1 | 8.5 | 8.8 | 42.3 | 97.8 | 95.6 |
| 58 | Ballerina | 28 | 163 | 52 | 15.8 | 37 | 132.5 | 1.07 | 87 | 0.96 | 1.18 | 1.07 | 1.05 | 1.21 | 1.13 | 64.3 | 74.4 | 69.35 | 55.1 | 68 | 61.55 | 44.9 | 32 | 38.45 | 6.3 | 14.4 | 10.35 | 38.9 | 47.7 | 43.3 | 8.3 | 5.7 | 7 | 68.3 | 95.8 | 97.6 |
| 59 | Ballerina | 28 | 168 | 55 | 19.5 | 36 | 106.3 | 1.1 | 97.6 | 1.1 | 1.15 | 1.125 | 1.2 | 1.29 | 1.245 | 73.4 | 79 | 76.2 | 57.5 | 58.4 | 57.95 | 42.5 | 41.6 | 42.05 | 9.6 | 8.5 | 9.05 | 40 | 41.1 | 40.55 | 7.8 | 6.3 | 7.05 | 81.8 | 98.8 | 98.2 |
| 60 | Ballerina | 28 | 172 | 65 | 22 | 38 | 108.7 | 1.1 | 96 | 1.02 | 1.13 | 1.075 | 1.16 | 1.14 | 1.15 | 71.2 | 69.9 | 70.55 | 55.7 | 61.7 | 58.7 | 44.3 | 38.3 | 41.3 | 8.4 | 10.3 | 9.35 | 36.3 | 40.3 | 38.3 | 8 | 6.4 | 7.2 | 49.6 | 19.1 | 44.7 |
| 61 | Ballerina | 28 | 176 | 66 | 21.3 | 37 | 96.6 | 1.31 | 96 | 1.28 | 1.31 | 1.295 | 1.58 | 1.74 | 1.66 | 90.2 | 99.4 | 94.8 | 64 | 59.5 | 61.75 | 36 | 40.5 | 38.25 | 10.5 | 12 | 11.25 | 41.3 | 36.4 | 38.85 | 5.3 | 5.9 | 5.6 | 75.1 | 96.2 | 79.3 |
| 62 | Ballerina | 28 | 177 | 81 | 25.9 | 39 | 107 | 1.48 | 86.6 | 1.33 | 1.13 | 1.23 | 1.84 | 1.69 | 1.765 | 105.2 | 96.6 | 100.9 | 62.1 | 65 | 63.55 | 37.9 | 35 | 36.45 | 9.3 | 12.2 | 10.75 | 39.9 | 40.3 | 40.1 | 8.5 | 7.3 | 7.9 | 53.7 | 95.5 | 81.9 |
| 63 | Ballerina | 29 | 162 | 45 | 17.1 | 36 | 96.7 | 1.34 | 83.7 | 1.24 | 1.26 | 1.25 | 1.67 | 1.65 | 1.66 | 95.2 | 94.4 | 94.8 | 57.2 | 62.6 | 59.9 | 42.8 | 37.4 | 40.1 | 8.6 | 12.4 | 10.5 | 37.5 | 41.3 | 39.4 | 6.7 | 6 | 6.35 | 23.8 | 95 | 95.6 |
| 64 | Ballerina | 29 | 162 | 46 | 17.5 | 36 | 102.9 | 1.7 | 87.4 | 1.18 | 1.16 | 1.17 | 2.04 | 1.94 | 1.99 | 117.9 | 112.2 | 115.05 | 61.6 | 57.7 | 59.65 | 38.4 | 42.3 | 40.35 | 9.1 | 8.9 | 9 | 42 | 39.8 | 40.9 | 6.1 | 7.1 | 6.6 | 79.6 | 97.8 | 97 |
| 65 | Ballerina | 29 | 165 | 59 | 21.7 | 36 | 100.4 | 1.49 | 97.9 | 1.19 | 1.2 | 1.195 | 1.86 | 1.68 | 1.77 | 107.4 | 97 | 102.2 | 62.3 | 61.1 | 61.7 | 37.7 | 38.9 | 38.3 | 11.3 | 12.4 | 11.85 | 38.8 | 37.2 | 38 | 7.4 | 8 | 7.7 | 87.3 | 99 | 97.6 |
| 66 | Ballerina | 29 | 165 | 65 | 23.9 | 38 | 103.4 | 1.7 | 93 | 1.17 | 1.12 | 1.145 | 2.01 | 1.82 | 1.915 | 116.2 | 105.5 | 110.85 | 59.9 | 56.6 | 58.25 | 40.1 | 43.4 | 41.75 | 7.9 | 11.5 | 9.7 | 41.2 | 36.6 | 38.9 | 6.2 | 6.9 | 6.55 | 85.3 | 97.9 | 97.8 |
| 67 | Ballerina | 29 | 180 | 59 | 18.2 | 40 | 115.5 | 1.32 | 97 | 1.04 | 1.05 | 1.045 | 1.37 | 1.39 | 1.38 | 84.3 | 85.1 | 84.7 | 58.7 | 58.5 | 58.6 | 41.3 | 41.5 | 41.4 | 7.4 | 9.9 | 8.65 | 41.4 | 41.1 | 41.25 | 9.4 | 10 | 9.7 | 49.1 | 98.6 | 93.1 |
| 68 | Ballerina | 29 | 183 | 64 | 19.1 | 40 | 115.5 | 1.28 | 96.7 | 1.04 | 1.03 | 1.035 | 1.32 | 1.33 | 1.325 | 80.9 | 81.5 | 81.2 | 58.9 | 58.7 | 58.8 | 41.1 | 41.3 | 41.2 | 9.6 | 8.2 | 8.9 | 40.9 | 40.8 | 40.85 | 8.1 | 11 | 9.55 | 41.2 | 97.7 | 91.6 |
| 69 | Ballerina | 30 | 161 | 62 | 23.9 | 36 | 118.8 | 1.41 | 96.5 | 1 | 1.01 | 1.005 | 1.43 | 1.42 | 1.425 | 87.8 | 86.9 | 87.35 | 59.1 | 56.5 | 57.8 | 40.9 | 43.5 | 42.2 | 7.6 | 8.9 | 8.25 | 43.2 | 40.1 | 41.65 | 9.5 | 11.1 | 10.3 | 51.7 | 97.4 | 77.6 |
| 70 | Ballerina | 30 | 164 | 63 | 23.4 | 38 | 109.3 | 1.36 | 95.8 | 1.1 | 1.1 | 1.1 | 1.42 | 1.57 | 1.495 | 85.7 | 94.9 | 90.3 | 59.1 | 62.3 | 60.7 | 40.9 | 37.7 | 39.3 | 11.5 | 12 | 11.75 | 37.8 | 38.8 | 38.3 | 7.3 | 6.7 | 7 | 59.5 | 97.9 | 98.9 |
| 71 | Ballerina | 30 | 175 | 82 | 26.8 | 40 | 107.9 | 1.35 | 94.9 | 1.08 | 1.13 | 1.105 | 1.43 | 1.5 | 1.465 | 86 | 90.3 | 88.15 | 58.9 | 60.9 | 59.9 | 41.1 | 39.1 | 40.1 | 11.6 | 12.2 | 11.9 | 35.8 | 37.9 | 36.85 | 7.6 | 6.9 | 7.25 | 61 | 97.7 | 97.9 |
| 72 | Ballerina | 31 | 169 | 61 | 21.4 | 37 | 114 | 1.6 | 95.7 | 1.1 | 1.06 | 1.08 | 1.6 | 1.83 | 1.715 | 96.2 | 110.3 | 103.25 | 62.4 | 62.1 | 62.25 | 37.6 | 37.9 | 37.75 | 13.8 | 12.1 | 12.95 | 37.3 | 38.6 | 37.95 | 6.6 | 6 | 6.3 | 67.7 | 99 | 98.4 |

| IDENTIFICATION | | DEMOGRAPHICS | | | | | GENERAL GAIT | | | Stride Duration (s) | | | Stride Length (m) | | | % Stride Length (% height) | | | Stance Duration (%) | | | Swing Duration (%) | | | Double Support (%) | | | Single Support (%) | | | Propulsion Index | | | Pelvis Symmetry (Symmetry Index) | | |
|---|---|---|---|---|---|---|---|---|---|---|---|---|---|---|---|---|---|---|---|---|---|---|---|---|---|---|---|---|---|---|---|---|---|---|---|---|---|---|---|
| ID | Condition | Age (yr) | Height (cm) | Weight (kg) | BMI (kg/m$^2$) | Shoe Size (EU) | Cadence (steps/min) | Speed (m/s) | Symmetry Index | Left | Right | Avg | Left | Right | Avg | Left | Right | Avg | Left | Right | Avg | Left | Right | Avg | First Left | First Right | Avg First | Left | Right | Avg | Left | Right | Avg | Tilt | Obliquity | Rotation |
| 73 | Ballerina | 31 | 171 | 70 | 23.9 | 39 | 109.8 | 1.1 | 95.5 | 1.09 | 1.1 | 1.095 | 1.2 | 1.2 | 1.2 | 66.9 | 66.9 | 66.9 | 59.4 | 55.9 | 57.65 | 40.6 | 44.1 | 42.35 | 6.7 | 9.5 | 8.1 | 44.2 | 39.6 | 41.9 | 6.6 | 5.9 | 6.25 | 87.7 | 95.5 | 98.9 |
| 74 | Ballerina | 32 | 165 | 50 | 18.4 | 37 | 110.8 | 1.13 | 95.8 | 1.09 | 1.08 | 1.085 | 1.23 | 1.23 | 1.23 | 68.5 | 68.2 | 68.35 | 59.2 | 57.2 | 58.2 | 40.8 | 42.8 | 41.8 | 6.7 | 9.5 | 8.1 | 42.9 | 41 | 41.95 | 5.6 | 5.6 | 5.6 | 89.5 | 96.9 | 99.4 |
| 75 | Ballerina | 32 | 177 | 60 | 19.2 | 39 | 109.8 | 1.12 | 95.3 | 1.09 | 1.1 | 1.095 | 1.24 | 1.23 | 1.235 | 69.1 | 68.1 | 68.6 | 57.2 | 55.8 | 56.5 | 42.8 | 44.2 | 43.5 | 6.7 | 7.9 | 7.3 | 44.6 | 41.3 | 42.95 | 6 | 4.7 | 5.35 | 83.8 | 97.1 | 99.2 |
| 1 | 8 cm Heel | 18 | 162 | 57 | 21.7 | 39 | 127.9 | 1.37 | 91.6 | 0.99 | 0.97 | 0.98 | 1.34 | 1.35 | 1.345 | 81.7 | 82.2 | 81.95 | 59.5 | 60 | 59.75 | 40.5 | 40 | 40.25 | 9.1 | 9.3 | 9.2 | 43.3 | 41.5 | 42.4 | 6.7 | 6.8 | 6.75 | 69.6 | 95.4 | 94.8 |
| 2 | 8 cm Heel | 19 | 161 | 52 | 20.1 | 38 | 121.5 | 1.45 | 84 | 1 | 1.06 | 1.03 | 1.41 | 1.58 | 1.495 | 86.1 | 96.2 | 91.15 | 61.3 | 56.9 | 59.1 | 38.7 | 43.1 | 40.9 | 8.5 | 12.8 | 10.65 | 45.7 | 36 | 40.85 | 6.1 | 9.1 | 7.6 | 61.4 | 89 | 91.6 |
| 3 | 8 cm Heel | 19 | 165 | 53 | 19.5 | 39 | 110.3 | 1.61 | 89.3 | 1.31 | 1.15 | 1.23 | 1.88 | 1.65 | 1.765 | 114.6 | 100.7 | 107.65 | 58.7 | 55.2 | 56.95 | 41.3 | 44.8 | 43.05 | 7.2 | 10.3 | 8.75 | 42.5 | 37.6 | 40.05 | 6.9 | 6.4 | 6.65 | 88 | 89.6 | 78.2 |
| 4 | 8 cm Heel | 19 | 168 | 48 | 17 | 37 | 113.6 | 2.34 | 89.8 | 1.12 | 1.14 | 1.13 | 2.24 | 2.63 | 2.435 | 135.5 | 159.3 | 147.4 | 58.6 | 60.6 | 59.6 | 41.4 | 39.4 | 40.4 | 6.7 | 12.9 | 9.8 | 39.3 | 41.2 | 40.25 | 8 | 8.8 | 8.4 | 69.9 | 95.4 | 88.9 |
| 5 | 8 cm Heel | 19 | 169 | 61 | 21.4 | 38 | 123.2 | 2.02 | 97 | 0.98 | 0.99 | 0.985 | 1.89 | 2.08 | 1.985 | 114.6 | 126 | 120.3 | 58.7 | 59.3 | 59 | 41.3 | 40.7 | 41 | 8.2 | 9.8 | 9 | 41.3 | 41.4 | 41.35 | 9.7 | 10.6 | 10.15 | 55.7 | 98.5 | 98.1 |
| 6 | 8 cm Heel | 19 | 172 | 59 | 19.9 | 39 | 125.7 | 2.27 | 96.5 | 0.97 | 1.14 | 1.055 | 2.12 | 2.41 | 2.265 | 128.6 | 146.3 | 137.45 | 58.4 | 61.2 | 59.8 | 41.6 | 38.8 | 40.2 | 6.3 | 7 | 6.65 | 43 | 47.6 | 45.3 | 10 | 8.2 | 9.1 | 60.2 | 98 | 98.3 |
| 7 | 8 cm Heel | 19 | 172 | 69 | 23.3 | 40 | 137.4 | 1.92 | 95.3 | 0.95 | 0.94 | 0.945 | 1.75 | 1.75 | 1.75 | 109.6 | 109.5 | 109.55 | 54.3 | 65.8 | 60.05 | 45.7 | 34.2 | 39.95 | 9.9 | 9.1 | 9.5 | 34.7 | 46.4 | 40.55 | 7.8 | 7.2 | 7.5 | 52.8 | 98.1 | 87.1 |
| 8 | 8 cm Heel | 19 | 173 | 67 | 22.4 | 38 | 140.5 | 1.5 | 90.5 | 0.99 | 0.94 | 0.965 | 1.43 | 1.4 | 1.415 | 89.4 | 87.8 | 88.6 | 62.9 | 65.7 | 64.3 | 37.1 | 34.3 | 35.7 | 10.7 | 16 | 13.35 | 37.2 | 37.5 | 37.35 | 12.5 | 9.7 | 11.1 | 25.4 | 97.1 | 86.1 |
| 9 | 8 cm Heel | 20 | 159 | 77 | 30.5 | 37 | 116.5 | 1.61 | 95.2 | 1.11 | 1.04 | 1.075 | 1.69 | 1.71 | 1.7 | 105.7 | 106.9 | 106.3 | 62.6 | 57.7 | 60.15 | 37.4 | 42.3 | 39.85 | 9.2 | 10.5 | 9.85 | 43.7 | 38.1 | 40.9 | 10 | 8.4 | 9.2 | 34.9 | 97 | 94.9 |
| 10 | 8 cm Heel | 20 | 163 | 61 | 23 | 39 | 118.1 | 1.35 | 75.3 | 1.01 | 1.04 | 1.025 | 1.36 | 1.42 | 1.39 | 83.8 | 87.8 | 85.8 | 57.3 | 64.3 | 60.8 | 42.7 | 35.7 | 39.2 | 9 | 16.1 | 12.55 | 31.8 | 41.5 | 36.65 | 9.5 | 5.5 | 7.5 | 55.1 | 89 | 90.9 |
| 11 | 8 cm Heel | 20 | 164 | 76 | 28.3 | 39 | 116.8 | 1.4 | 95.9 | 1.02 | 1.02 | 1.02 | 1.43 | 1.45 | 1.44 | 88.1 | 89.2 | 88.65 | 56.7 | 57.9 | 57.3 | 43.3 | 42.1 | 42.7 | 7.5 | 7.3 | 7.4 | 41.7 | 43.2 | 42.45 | 5.6 | 4.7 | 5.15 | 70.4 | 99.3 | 98.7 |
| 12 | 8 cm Heel | 20 | 167 | 58 | 20.8 | 37 | 107.5 | 1.34 | 88.8 | 1.1 | 1.09 | 1.095 | 1.44 | 1.49 | 1.465 | 89 | 92.1 | 90.55 | 55.8 | 60.8 | 58.3 | 44.2 | 39.2 | 41.7 | 7.5 | 13.2 | 10.35 | 37.2 | 40.1 | 38.65 | 5.9 | 4.8 | 5.35 | 50.1 | 94.3 | 94.9 |
| 13 | 8 cm Heel | 20 | 169 | 65 | 22.8 | 37 | 123.7 | 1.8 | 98.8 | 0.97 | 0.97 | 0.97 | 1.78 | 1.73 | 1.755 | 108.3 | 105.5 | 106.9 | 60.3 | 60.1 | 60.2 | 39.7 | 39.9 | 39.8 | 10.9 | 10.3 | 10.6 | 39.1 | 39.9 | 39.5 | 12.1 | 13.3 | 12.7 | 88 | 98.1 | 96.9 |
| 14 | 8 cm Heel | 21 | 158 | 57 | 22.8 | 36 | 134.1 | 0.93 | 96.5 | 0.98 | 0.98 | 0.98 | 0.92 | 0.91 | 0.915 | 56 | 55.5 | 55.75 | 60.2 | 60.1 | 60.15 | 39.8 | 39.9 | 39.85 | 11.4 | 9.5 | 10.45 | 39.7 | 39.1 | 39.4 | 8.5 | 9.4 | 8.95 | 97.3 | 93.2 | 76.2 |
| 15 | 8 cm Heel | 21 | 163 | 57 | 21.5 | 38 | 124.5 | 1.77 | 97.5 | 0.98 | 0.97 | 0.975 | 1.74 | 1.7 | 1.72 | 105.9 | 103.5 | 104.7 | 59.9 | 61.4 | 60.65 | 40.1 | 38.6 | 39.35 | 11.7 | 10.4 | 11.05 | 37.9 | 40.7 | 39.3 | 13.8 | 12.5 | 13.15 | 92 | 97.3 | 94.5 |
| 16 | 8 cm Heel | 21 | 165 | 60 | 22 | 37 | 102.9 | 1.89 | 87.5 | 1.18 | 1.15 | 1.165 | 2.02 | 2.2 | 2.11 | 121.5 | 132.7 | 127.1 | 63.2 | 60.6 | 61.9 | 36.8 | 39.4 | 38.1 | 15.1 | 9.7 | 12.4 | 37.2 | 36.5 | 36.85 | 6.7 | 6.9 | 6.8 | 65 | 96 | 88.8 |
| 17 | 8 cm Heel | 21 | 165 | 63 | 23.1 | 38 | 104.8 | 1.37 | 84.3 | 1.21 | 1.27 | 1.24 | 1.65 | 1.65 | 1.65 | 99.2 | 99.5 | 99.35 | 65.7 | 56.1 | 60.9 | 34.3 | 43.9 | 39.1 | 11.2 | 6.8 | 9 | 45.3 | 38.5 | 41.9 | 4.8 | 5.5 | 5.15 | 64.1 | 94 | 83 |
| 18 | 8 cm Heel | 21 | 172 | 73 | 24.7 | 40 | 108.1 | 1.58 | 83.9 | 1.17 | 1.12 | 1.145 | 1.86 | 1.77 | 1.815 | 112.3 | 106.4 | 109.35 | 64.3 | 58.7 | 61.5 | 35.7 | 41.3 | 38.5 | 14.5 | 8.4 | 11.45 | 41.5 | 37 | 39.25 | 4.1 | 5.4 | 4.75 | 55.3 | 95.8 | 90.9 |
| 19 | 8 cm Heel | 21 | 176 | 62 | 20 | 37 | 112.7 | 1.44 | 91.4 | 1.13 | 1.15 | 1.14 | 1.62 | 1.66 | 1.64 | 95.3 | 97.6 | 96.45 | 67.5 | 56.2 | 61.85 | 32.5 | 43.8 | 38.15 | 10.4 | 14.5 | 12.45 | 42.6 | 31 | 36.8 | 7.5 | 8.8 | 8.15 | 28.6 | 96 | 41.9 |
| 20 | 8 cm Heel | 21 | 177 | 65 | 20.7 | 39 | 113.9 | 1.35 | 94.1 | 1.08 | 1.06 | 1.07 | 1.46 | 1.43 | 1.445 | 85.9 | 84.2 | 85.05 | 60.2 | 63 | 61.6 | 39.8 | 37 | 38.4 | 12 | 11 | 11.5 | 37 | 40.8 | 38.9 | 10.7 | 9.4 | 10.05 | 74.4 | 97.8 | 97.9 |
| 21 | 8 cm Heel | 22 | 157 | 51 | 20.7 | 36 | 95.2 | 1.06 | 89.9 | 1.35 | 1.22 | 1.285 | 1.3 | 1.3 | 1.3 | 76.4 | 76.3 | 76.35 | 59.4 | 59.9 | 59.65 | 40.6 | 40.1 | 40.35 | 10.5 | 13.8 | 12.15 | 36.8 | 35 | 35.9 | 8 | 7.2 | 7.6 | 54 | 96.1 | 75.8 |
| 22 | 8 cm Heel | 22 | 162 | 52 | 19.8 | 38 | 118.7 | 0.92 | 84.3 | 1.18 | 1.15 | 1.165 | 1.08 | 1.05 | 1.065 | 63.6 | 61.7 | 62.65 | 64.2 | 56.9 | 60.55 | 35.8 | 43.1 | 39.45 | 10.7 | 9.6 | 10.15 | 44.4 | 36.3 | 40.35 | 4.5 | 7.1 | 5.8 | 20.8 | 92.9 | 32.6 |
| 23 | 8 cm Heel | 22 | 162 | 53 | 20.2 | 39 | 133.1 | 1.54 | 94.4 | 0.99 | 0.94 | 0.965 | 1.48 | 1.47 | 1.475 | 87.2 | 86.4 | 86.8 | 61 | 63.3 | 62.15 | 39 | 36.7 | 37.85 | 8 | 11.9 | 9.95 | 41.6 | 41.6 | 41.6 | 6.2 | 8.8 | 7.5 | 87.4 | 97.9 | 94 |
| 24 | 8 cm Heel | 22 | 162 | 56 | 21.3 | 37 | 114.2 | 1.89 | 91.5 | 1.12 | 1.1 | 1.11 | 1.11 | 1.18 | 1.145 | 93.9 | 92.2 | 93.05 | 57.6 | 64.9 | 61.25 | 42.4 | 35.1 | 38.75 | 10.4 | 11.7 | 11.05 | 35.7 | 43.1 | 39.4 | 7.2 | 6.7 | 6.95 | 67.3 | 94.5 | 53.5 |
| 25 | 8 cm Heel | 22 | 168 | 63 | 22.3 | 38 | 121.7 | 1.95 | 96.9 | 1 | 0.99 | 0.995 | 1.91 | 2 | 1.955 | 110.6 | 115.3 | 112.95 | 61 | 61.6 | 61.3 | 39 | 38.4 | 38.7 | 10 | 11.1 | 10.55 | 40 | 40.4 | 40.2 | 12.6 | 11.8 | 12.2 | 79.2 | 97.9 | 95.2 |
| 26 | 8 cm Heel | 22 | 168 | 63 | 22.3 | 39 | 118.5 | 1.86 | 98 | 1.02 | 1.03 | 1.025 | 1.92 | 1.9 | 1.91 | 111.2 | 109.8 | 110.5 | 60.1 | 60.1 | 60.1 | 39.9 | 39.9 | 39.9 | 10 | 9.7 | 9.85 | 40.6 | 40.6 | 40.6 | 12.5 | 11.1 | 11.8 | 85.8 | 98.1 | 95.9 |
| 27 | 8 cm Heel | 22 | 170 | 64 | 22.1 | 37 | 118.1 | 1.75 | 98.2 | 0.98 | 0.99 | 0.985 | 1.65 | 1.86 | 1.755 | 95.1 | 107.5 | 101.3 | 61.9 | 61.6 | 61.75 | 38.1 | 38.4 | 38.25 | 10.9 | 11.1 | 11 | 35.5 | 39.4 | 37.45 | 9.1 | 11.1 | 10.1 | 68.1 | 97 | 92.4 |
| 28 | 8 cm Heel | 22 | 173 | 59 | 19.7 | 37 | 107.9 | 1.54 | 92.6 | 1.13 | 1.12 | 1.125 | 1.74 | 1.7 | 1.72 | 99.6 | 97.2 | 98.4 | 58.6 | 61.6 | 60.1 | 41.4 | 38.4 | 39.9 | 10.1 | 12 | 11.05 | 36.6 | 41.5 | 39.05 | 9.6 | 10.3 | 9.95 | 61.6 | 96.1 | 96.7 |
| 29 | 8 cm Heel | 23 | 163 | 81 | 30.5 | 38 | 111.2 | 1.65 | 97.2 | 1.11 | 1.08 | 1.095 | 1.82 | 1.79 | 1.805 | 104.3 | 102.2 | 103.25 | 59.9 | 62.5 | 61.2 | 40.1 | 37.5 | 38.8 | 11.7 | 9.7 | 10.7 | 38.7 | 41.2 | 39.95 | 11.7 | 8.8 | 10.25 | 34.3 | 98.8 | 97.5 |
| 30 | 8 cm Heel | 23 | 164 | 48 | 17.8 | 38 | 106.9 | 1.69 | 94.8 | 1.13 | 1.13 | 1.13 | 1.92 | 1.9 | 1.91 | 109.7 | 108.4 | 109.05 | 60.4 | 60.1 | 60.25 | 39.6 | 39.9 | 39.75 | 8 | 11.2 | 9.6 | 41 | 39.5 | 40.25 | 9.3 | 9.3 | 9.3 | 44 | 96.9 | 84.8 |
| 31 | 8 cm Heel | 23 | 166 | 60 | 21.8 | 38 | 132.5 | 1.74 | 94.1 | 0.89 | 0.91 | 0.9 | 1.56 | 1.57 | 1.565 | 97.3 | 98.1 | 97.7 | 67.7 | 65 | 66.35 | 32.3 | 35 | 33.65 | 18 | 16.5 | 17.25 | 32.7 | 31.9 | 32.3 | 11.6 | 11.9 | 11.75 | 43.5 | 96.4 | 86.3 |
| 32 | 8 cm Heel | 23 | 167 | 50 | 17.9 | 37 | 137 | 1.81 | 90.9 | 0.88 | 0.88 | 0.88 | 1.6 | 1.58 | 1.59 | 100 | 98.7 | 99.35 | 68.4 | 62.8 | 65.6 | 31.6 | 37.2 | 34.4 | 17.1 | 14.2 | 15.65 | 36.2 | 32.7 | 34.45 | 10.5 | 13 | 11.75 | 64.2 | 98 | 88.4 |
| 33 | 8 cm Heel | 23 | 172 | 57 | 19.3 | 39 | 137.8 | 1.79 | 78.5 | 0.89 | 0.89 | 0.89 | 1.61 | 1.57 | 1.59 | 100.7 | 98 | 99.35 | 67.1 | 61.7 | 64.4 | 32.9 | 38.3 | 35.6 | 17.9 | 11.3 | 14.6 | 37.9 | 33 | 35.45 | 11.8 | 14.1 | 12.95 | 70.7 | 94.5 | 67 |
| 34 | 8 cm Heel | 23 | 175 | 53 | 17.3 | 38 | 117.7 | 1.19 | 96.5 | 1.03 | 1.02 | 1.025 | 1.22 | 1.22 | 1.22 | 69.8 | 69.7 | 69.75 | 71.9 | 69.5 | 70.7 | 28.1 | 30.5 | 29.3 | 21.1 | 20.6 | 20.85 | 30.3 | 27.4 | 28.85 | 12.6 | 11.1 | 11.85 | 86.4 | 95.7 | 97.8 |
| 35 | 8 cm Heel | 23 | 175 | 63 | 20.6 | 37 | 119.6 | 1.22 | 84.2 | 1.02 | 1.03 | 1.025 | 1.24 | 1.23 | 1.235 | 70.9 | 70.5 | 70.7 | 70.3 | 64.2 | 67.25 | 29.7 | 35.8 | 32.75 | 19.7 | 13.9 | 16.8 | 36.3 | 29.7 | 33 | 8.1 | 11.9 | 10 | 63.3 | 91.2 | 93.5 |
| 36 | 8 cm Heel | 23 | 175 | 68 | 22.2 | 39 | 122.1 | 1.18 | 97.1 | 1.04 | 1.04 | 1.04 | 1.21 | 1.24 | 1.225 | 69.1 | 71.1 | 70.1 | 70.3 | 67.8 | 69.05 | 29.7 | 32.2 | 30.95 | 18.9 | 19 | 18.95 | 33.6 | 31 | 32.3 | 10.5 | 10.5 | 10.5 | 76.1 | 97.4 | 98.9 |
| 37 | 8 cm Heel | 24 | 159 | 56 | 22.2 | 36 | 117 | 1.49 | 97.1 | 1.03 | 1.03 | 1.03 | 1.53 | 1.53 | 1.53 | 93.5 | 93.2 | 93.35 | 62.9 | 64.6 | 63.75 | 37.1 | 35.4 | 36.25 | 13.7 | 14.6 | 14.15 | 35.3 | 36.4 | 35.85 | 11.2 | 10.4 | 10.8 | 96 | 97.8 | 96.4 |
| 38 | 8 cm Heel | 24 | 163 | 61 | 23 | 37 | 115.8 | 1.49 | 98 | 1.03 | 1.04 | 1.035 | 1.55 | 1.54 | 1.545 | 94.4 | 93.7 | 94.05 | 61.2 | 60.6 | 60.9 | 38.8 | 39.4 | 39.1 | 9.5 | 12.7 | 11.1 | 39.5 | 37.8 | 38.65 | 10.4 | 10.4 | 10.4 | 97.4 | 97.8 | 96.1 |
| 39 | 8 cm Heel | 24 | 166 | 51 | 18.5 | 36 | 114.9 | 1.42 | 95.7 | 1.05 | 1.05 | 1.05 | 1.49 | 1.49 | 1.49 | 90.6 | 90.7 | 90.65 | 62.1 | 62.3 | 62.2 | 37.9 | 37.7 | 37.8 | 9.6 | 13.4 | 11.5 | 38.9 | 38 | 38.45 | 11.4 | 9.6 | 10.5 | 94.5 | 98.4 | 99 |
| 40 | 8 cm Heel | 24 | 167 | 46 | 16.5 | 36 | 122.9 | 1.43 | 97.8 | 1 | 1 | 1 | 1.43 | 1.44 | 1.435 | 83.9 | 84.6 | 84.25 | 70.6 | 69.6 | 70.1 | 29.4 | 30.4 | 29.9 | 19.9 | 20.5 | 20.2 | 31.2 | 29.7 | 30.45 | 10.7 | 10.6 | 10.65 | 56.5 | 97.6 | 96.1 |
| 41 | 8 cm Heel | 24 | 171 | 55 | 18.8 | 38 | 122.5 | 1.4 | 73.4 | 1.03 | 1.01 | 1.02 | 1.43 | 1.43 | 1.43 | 83.9 | 84.3 | 84.1 | 69.9 | 63.2 | 66.55 | 30.1 | 36.8 | 33.45 | 21.7 | 12.4 | 17.05 | 36 | 29.4 | 32.7 | 8.3 | 12.6 | 10.45 | 75.8 | 91.1 | 91.2 |
| 42 | 8 cm Heel | 24 | 172 | 61 | 20.6 | 38 | 125.8 | 1.53 | 71 | 0.99 | 0.98 | 0.985 | 1.51 | 1.5 | 1.505 | 88.5 | 88.5 | 88.5 | 71 | 62.6 | 66.8 | 29 | 37.4 | 33.2 | 20.6 | 13.2 | 16.9 | 37.2 | 29.3 | 33.25 | 8.7 | 12.6 | 10.65 | 71.3 | 89.7 | 94.8 |
| 43 | 8 cm Heel | 24 | 177 | 66 | 21.1 | 39 | 117.1 | 1.43 | 89.9 | 1.02 | 1.03 | 1.025 | 1.47 | 1.44 | 1.455 | 84.8 | 83.3 | 84.05 | 57.6 | 60.8 | 59.2 | 42.4 | 39.2 | 40.8 | 8.6 | 10 | 9.3 | 38.9 | 41.9 | 40.4 | 11.9 | 8.2 | 10.05 | 67.3 | 97.5 | 98.9 |
| 44 | 8 cm Heel | 25 | 162 | 54 | 20.6 | 37 | 117.8 | 1.43 | 95.8 | 1.02 | 1.01 | 1.015 | 1.46 | 1.45 | 1.455 | 84.2 | 84.1 | 84.15 | 58.4 | 59.9 | 59.15 | 41.6 | 40.1 | 40.85 | 9 | 9.6 | 9.3 | 39.9 | 41.4 | 40.65 | 11.5 | 9.4 | 10.45 | 95.3 | 98.2 | 98.9 |
| 45 | 8 cm Heel | 25 | 169 | 59 | 20.7 | 38 | 116.9 | 1.44 | 92.4 | 1.02 | 1.02 | 1.02 | 1.47 | 1.47 | 1.47 | 84.8 | 84.8 | 84.8 | 59.2 | 61.1 | 60.15 | 40.8 | 38.9 | 39.85 | 9 | 11.4 | 10.2 | 38.9 | 40.5 | 39.7 | 11.6 | 7.8 | 9.7 | 81.1 | 98.3 | 98.7 |
| 46 | 8 cm Heel | 25 | 172 | 54 | 18.3 | 36 | 127 | 1.56 | 92.5 | 0.95 | 0.94 | 0.945 | 1.47 | 1.49 | 1.48 | 89.4 | 90.6 | 90 | 60.4 | 57.6 | 59 | 39.6 | 42.4 | 41 | 8.7 | 9.5 | 9.1 | 42.2 | 39.2 | 40.7 | 13 | 13.6 | 13.3 | 59.8 | 97.6 | 97.1 |
| 47 | 8 cm Heel | 26 | 162 | 47 | 17.9 | 36 | 130.1 | 1.58 | 92.7 | 0.93 | 0.93 | 0.93 | 1.46 | 1.46 | 1.46 | 88.8 | 89 | 88.9 | 61.6 | 57.8 | 59.7 | 38.4 | 42.2 | 40.3 | 9.4 | 10.3 | 9.85 | 42.2 | 38.1 | 40.15 | 12.7 | 15.2 | 13.95 | 49.7 | 98.6 | 97.9 |

| IDENTIFICATION | | DEMOGRAPHICS | | | | | GENERAL GAIT | | | Stride Duration (s) | | | Stride Length (m) | | | % Stride Length (% height) | | | Stance Duration (%) | | | Swing Duration (%) | | | Double Support (%) | | | Single Support (%) | | | Propulsion Index | | | Pelvis Symmetry (Symmetry Index) | | |
|---|---|---|---|---|---|---|---|---|---|---|---|---|---|---|---|---|---|---|---|---|---|---|---|---|---|---|---|---|---|---|---|---|---|---|---|---|---|---|---|
| ID | Condition | Age (yr) | Height (cm) | Weight (kg) | BMI (kg/m$^2$) | Shoe Size (EU) | Cadence (steps/min) | Speed (m/s) | Symmetry Index | Left | Right | Avg | Left | Right | Avg | Left | Right | Avg | Left | Right | Avg | Left | Right | Avg | First Left | First Right | Avg First | Left | Right | Avg | Left | Right | Avg | Tilt | Obliquity | Rotation |
| 48 | 8 cm Heel | 26 | 164 | 50 | 18.6 | 37 | 128.5 | 1.6 | 93 | 0.94 | 0.94 | 0.94 | 1.49 | 1.51 | 1.5 | 91.1 | 91.8 | 91.45 | 61.9 | 57.9 | 59.9 | 38.1 | 42.1 | 40.1 | 9.6 | 9.9 | 9.75 | 42.4 | 38.7 | 40.55 | 13.9 | 14.3 | 14.1 | 43.3 | 98.5 | 98.2 |
| 49 | 8 cm Heel | 26 | 169 | 58 | 20.3 | 37 | 125 | 1.42 | 85.7 | 1 | 0.97 | 0.985 | 1.39 | 1.41 | 1.4 | 81.2 | 82.6 | 81.9 | 64.4 | 62.9 | 63.65 | 35.6 | 37.1 | 36.35 | 18.8 | 9.3 | 14.05 | 36.3 | 35.4 | 35.85 | 8 | 10.4 | 9.2 | 85.4 | 95.2 | 95.5 |
| 50 | 8 cm Heel | 26 | 172 | 55 | 18.6 | 37 | 120.3 | 1.36 | 86.5 | 1 | 1.02 | 1.01 | 1.38 | 1.37 | 1.375 | 80.8 | 80.2 | 80.5 | 66.6 | 62.9 | 64.75 | 33.4 | 37.1 | 35.25 | 19.3 | 11.9 | 15.6 | 35.2 | 32.9 | 34.05 | 8.8 | 11 | 9.9 | 83.3 | 94.1 | 93.5 |
| 51 | 8 cm Heel | 26 | 173 | 77 | 25.7 | 40 | 121.7 | 1.37 | 87.6 | 1.02 | 1 | 1.01 | 1.37 | 1.39 | 1.38 | 80.4 | 81 | 80.7 | 65.2 | 61.3 | 63.25 | 34.8 | 38.7 | 36.75 | 17 | 10.5 | 13.75 | 37.9 | 35.6 | 36.75 | 7.2 | 9.7 | 8.45 | 86 | 94.9 | 96.1 |
| 52 | 8 cm Heel | 27 | 158 | 61 | 24.4 | 37 | 122.2 | 1.57 | 92 | 0.98 | 0.98 | 0.98 | 1.55 | 1.55 | 1.55 | 93.8 | 93.6 | 93.7 | 61.4 | 63.8 | 62.6 | 38.6 | 36.2 | 37.4 | 10.2 | 12.6 | 11.4 | 38.6 | 38.6 | 38.6 | 11.8 | 9.9 | 10.85 | 17.5 | 96 | 97.8 |
| 53 | 8 cm Heel | 27 | 162 | 64 | 24.4 | 37 | 119.9 | 1.51 | 96.3 | 1.03 | 1.01 | 1.02 | 1.54 | 1.53 | 1.535 | 93.4 | 92.8 | 93.1 | 60 | 62.2 | 61.1 | 40 | 37.8 | 38.9 | 11.5 | 10.5 | 11 | 38.3 | 40.9 | 39.6 | 10.2 | 9.1 | 9.65 | 53.5 | 95.8 | 99.2 |
| 54 | 8 cm Heel | 27 | 163 | 51 | 19.2 | 36 | 116.2 | 1.51 | 94.9 | 1.03 | 1.03 | 1.03 | 1.55 | 1.56 | 1.555 | 94.1 | 94.7 | 94.4 | 59.4 | 60.3 | 59.85 | 40.6 | 39.7 | 40.15 | 10 | 10.3 | 10.15 | 39 | 40.6 | 39.8 | 9.9 | 8.3 | 9.1 | 30.8 | 97.8 | 99.1 |
| 55 | 8 cm Heel | 27 | 164 | 66 | 24.5 | 36 | 129.3 | 1.69 | 87.1 | 0.92 | 0.93 | 0.925 | 1.59 | 1.53 | 1.56 | 98.7 | 95 | 96.85 | 62.8 | 61 | 61.9 | 37.2 | 39 | 38.1 | 17.5 | 10.2 | 13.85 | 37.3 | 33.7 | 35.5 | 12 | 11.3 | 11.65 | 19 | 96.8 | 96.1 |
| 56 | 8 cm Heel | 27 | 172 | 57 | 19.3 | 39 | 148.2 | 1.8 | 38.1 | 0.89 | 0.89 | 0.89 | 1.6 | 1.59 | 1.595 | 99.2 | 98.8 | 99 | 73 | 59.8 | 66.4 | 27 | 40.2 | 33.6 | 27.8 | 8.3 | 18.05 | 36.9 | 27.1 | 32 | 12.7 | 17.7 | 15.2 | 29.5 | 80.9 | 74.8 |
| 57 | 8 cm Heel | 28 | 162 | 60 | 22.9 | 36 | 130.6 | 1.66 | 84.7 | 0.95 | 0.93 | 0.94 | 1.55 | 1.54 | 1.545 | 96.5 | 95.9 | 96.2 | 62 | 64.8 | 63.4 | 38 | 35.2 | 36.6 | 20.2 | 7.9 | 14.05 | 34 | 38.8 | 36.4 | 11.2 | 8.5 | 9.85 | 83.9 | 96.4 | 98 |
| 58 | 8 cm Heel | 28 | 163 | 52 | 15.8 | 37 | 106.9 | 1.58 | 95.4 | 1.02 | 1.13 | 1.075 | 1.64 | 1.68 | 1.66 | 100.8 | 103 | 101.9 | 58.7 | 58.9 | 58.8 | 41.3 | 41.1 | 41.2 | 11.8 | 11.5 | 11.65 | 33.7 | 37.3 | 35.5 | 8.5 | 6.6 | 7.55 | 40.5 | 97.3 | 96 |
| 59 | 8 cm Heel | 28 | 168 | 55 | 19.5 | 36 | 111.5 | 1.43 | 93.8 | 1.13 | 1.06 | 1.095 | 1.53 | 1.58 | 1.555 | 94 | 97.2 | 95.6 | 62.1 | 57.3 | 59.7 | 37.9 | 42.7 | 40.3 | 7.7 | 10.4 | 9.05 | 44.7 | 37.7 | 41.2 | 8 | 8.9 | 8.45 | 58.6 | 98.7 | 97.9 |
| 60 | 8 cm Heel | 28 | 172 | 65 | 22 | 38 | 117.4 | 1.32 | 91.3 | 1 | 0.98 | 0.99 | 1.2 | 1.32 | 1.26 | 73.7 | 81 | 77.35 | 58 | 60 | 59 | 42 | 40 | 41 | 8 | 9.8 | 8.9 | 40 | 38 | 39 | 10 | 9.2 | 9.6 | 63.6 | 97.9 | 97.8 |
| 61 | 8 cm Heel | 28 | 176 | 66 | 21.3 | 37 | 94.9 | 1.36 | 96.6 | 1.29 | 1.25 | 1.27 | 1.74 | 1.68 | 1.71 | 99.2 | 95.8 | 97.5 | 62.1 | 61.8 | 61.95 | 37.9 | 38.2 | 38.05 | 11.4 | 13.6 | 12.5 | 37.2 | 37.9 | 37.55 | 6.7 | 7.2 | 6.95 | 40.1 | 96.9 | 93 |
| 62 | 8 cm Heel | 28 | 177 | 81 | 25.9 | 39 | 90.6 | 1.4 | 94.8 | 1.39 | 1.56 | 1.475 | 1.92 | 1.69 | 1.805 | 109.9 | 96.6 | 103.25 | 60.2 | 58 | 59.1 | 39.8 | 42 | 40.9 | 7.6 | 12.2 | 9.9 | 44.3 | 38.3 | 41.3 | 7.4 | 8.2 | 7.8 | 7.8 | 96 | 83.9 |
| 63 | 8 cm Heel | 29 | 162 | 45 | 17.1 | 36 | 94.4 | 1.44 | 95.7 | 1.36 | 1.41 | 1.385 | 1.99 | 1.8 | 1.895 | 113.6 | 103.1 | 108.35 | 57.5 | 57.6 | 57.55 | 42.5 | 42.4 | 42.45 | 6.2 | 6.4 | 6.3 | 44.5 | 45.3 | 44.9 | 6.4 | 6.2 | 6.3 | 26.9 | 96.9 | 89.6 |
| 64 | 8 cm Heel | 29 | 162 | 46 | 17.5 | 36 | 104.2 | 1.66 | 83 | 1.2 | 1.31 | 1.255 | 2.15 | 1.86 | 2.005 | 124.3 | 107.4 | 115.85 | 55.8 | 57.3 | 56.55 | 44.2 | 42.7 | 43.45 | 7.5 | 7.2 | 7.35 | 41 | 42 | 41.5 | 6.4 | 6.3 | 6.35 | 63.7 | 97.5 | 94.7 |
| 65 | 8 cm Heel | 29 | 165 | 59 | 21.7 | 36 | 100.5 | 1.67 | 96.2 | 1.21 | 1.16 | 1.185 | 2.08 | 1.83 | 1.955 | 120 | 105.5 | 112.75 | 57.2 | 58.3 | 57.75 | 42.8 | 41.7 | 42.25 | 8.7 | 8.5 | 8.6 | 40 | 40.2 | 40.1 | 7.2 | 6.7 | 6.95 | 72.9 | 97.7 | 97.8 |
| 66 | 8 cm Heel | 29 | 165 | 65 | 23.9 | 38 | 102.4 | 1.93 | 91.3 | 1.18 | 1.24 | 1.21 | 2.52 | 2.14 | 2.33 | 145.8 | 123.7 | 134.75 | 57.7 | 58 | 57.85 | 42.3 | 42 | 42.15 | 7.8 | 4.8 | 6.3 | 43.9 | 46 | 44.95 | 6.8 | 6.3 | 6.55 | 79.6 | 98 | 98.9 |
| 67 | 8 cm Heel | 29 | 180 | 59 | 18.2 | 40 | 113.7 | 1.41 | 97.1 | 1.05 | 1.06 | 1.055 | 1.5 | 1.46 | 1.48 | 92.1 | 89.3 | 90.7 | 61.8 | 59.9 | 60.85 | 38.2 | 40.1 | 39.15 | 10.8 | 11.4 | 11.1 | 39.5 | 37.9 | 38.7 | 9.7 | 11.7 | 10.7 | 59.1 | 95.9 | 91 |
| 68 | 8 cm Heel | 29 | 183 | 64 | 19.1 | 40 | 112.4 | 1.36 | 97.7 | 1.06 | 1.07 | 1.065 | 1.45 | 1.46 | 1.455 | 89 | 89.6 | 89.3 | 61.6 | 62.7 | 62.15 | 38.4 | 37.3 | 37.85 | 13.2 | 11.6 | 12.4 | 37.1 | 38 | 37.55 | 10 | 11.4 | 10.7 | 72.4 | 95.7 | 96.3 |
| 69 | 8 cm Heel | 30 | 161 | 62 | 23.9 | 36 | 114.7 | 1.49 | 96.6 | 1.05 | 1.05 | 1.05 | 1.57 | 1.54 | 1.555 | 96.4 | 94.7 | 95.55 | 61.9 | 60.8 | 61.35 | 38.1 | 39.2 | 38.65 | 11.1 | 11.5 | 11.3 | 39.4 | 38.2 | 38.8 | 9.6 | 12.8 | 11.2 | 72.4 | 93.9 | 91.8 |
| 70 | 8 cm Heel | 30 | 164 | 63 | 23.4 | 38 | 112.9 | 1.8 | 91 | 1.08 | 1.09 | 1.085 | 1.78 | 2.05 | 1.915 | 107.3 | 123.4 | 115.35 | 58.2 | 63.4 | 60.8 | 41.8 | 36.6 | 39.2 | 9.9 | 12.1 | 11 | 37 | 41.5 | 39.25 | 7.6 | 5.1 | 6.35 | 40.3 | 96.9 | 98.2 |
| 71 | 8 cm Heel | 30 | 175 | 82 | 26.8 | 40 | 121.2 | 1.42 | 92.6 | 0.99 | 0.98 | 0.985 | 1.39 | 1.4 | 1.395 | 83.8 | 84.1 | 83.95 | 59.9 | 56.8 | 58.35 | 40.1 | 43.2 | 41.65 | 8.6 | 7.8 | 8.2 | 43.2 | 40.1 | 41.65 | 7 | 6.9 | 6.95 | 75 | 97 | 98 |
| 72 | 8 cm Heel | 31 | 169 | 61 | 21.4 | 37 | 110.8 | 1.69 | 96 | 1.12 | 1.09 | 1.105 | 1.74 | 1.92 | 1.83 | 104.6 | 115.5 | 110.05 | 60.3 | 59 | 59.65 | 39.7 | 41 | 40.35 | 10.1 | 9.3 | 9.7 | 41.1 | 40.9 | 41 | 8 | 6.4 | 7.2 | 60.1 | 98.6 | 99.1 |
| 73 | 8 cm Heel | 31 | 171 | 70 | 23.9 | 39 | 111.7 | 1.26 | 94.7 | 1.08 | 1.08 | 1.08 | 1.36 | 1.34 | 1.35 | 75.8 | 74.6 | 75.2 | 61.7 | 59.3 | 60.5 | 38.3 | 40.7 | 39.5 | 8.4 | 12.3 | 10.35 | 41.1 | 38.4 | 39.75 | 5.8 | 7.3 | 6.55 | 82.5 | 93.6 | 99.2 |
| 74 | 8 cm Heel | 32 | 165 | 50 | 18.4 | 37 | 112.8 | 1.25 | 96.2 | 1.06 | 1.07 | 1.065 | 1.35 | 1.33 | 1.34 | 74.8 | 73.9 | 74.35 | 62.4 | 56.7 | 59.55 | 37.6 | 43.3 | 40.45 | 7.3 | 11.9 | 9.6 | 43.5 | 37.7 | 40.6 | 6.2 | 6.6 | 6.4 | 83.4 | 93.4 | 99 |
| 75 | 8 cm Heel | 32 | 177 | 60 | 19.2 | 39 | 110.6 | 1.18 | 98 | 1.09 | 1.1 | 1.095 | 1.29 | 1.29 | 1.29 | 71.5 | 71.4 | 71.45 | 61.6 | 59.8 | 60.7 | 38.4 | 40.2 | 39.3 | 10.7 | 11.4 | 11.05 | 39.9 | 38.6 | 39.25 | 6.4 | 7.1 | 6.75 | 90.6 | 94.9 | 99.1 |
| 1 | 12 cm Heel | 18 | 162 | 57 | 21.7 | 39 | 126.3 | 1.41 | 97.8 | 1 | 0.95 | 0.975 | 1.36 | 1.39 | 1.375 | 82.7 | 84.6 | 83.65 | 56.7 | 58.1 | 57.4 | 43.3 | 41.9 | 42.6 | 8.5 | 6.3 | 7.4 | 42.3 | 45.5 | 43.9 | 6.1 | 4.3 | 5.2 | 94.8 | 98.9 | 97.6 |
| 2 | 12 cm Heel | 19 | 161 | 52 | 20.1 | 38 | 121.3 | 1.36 | 89.4 | 1.02 | 0.99 | 1.005 | 1.37 | 1.37 | 1.37 | 83.4 | 83.3 | 83.35 | 58.7 | 55.4 | 57.05 | 41.3 | 44.6 | 42.95 | 9.5 | 5.7 | 7.6 | 43.6 | 42.3 | 42.95 | 5.4 | 5.7 | 5.55 | 67.4 | 95.6 | 88.9 |
| 3 | 12 cm Heel | 19 | 165 | 53 | 19.5 | 39 | 128.3 | 1.51 | 65.7 | 1.07 | 1.04 | 1.055 | 1.59 | 1.6 | 1.595 | 97 | 97.4 | 97.2 | 59.3 | 64.2 | 61.75 | 40.7 | 35.8 | 38.25 | 12.4 | 9.7 | 11.05 | 38.5 | 42.9 | 40.7 | 5.2 | 6.6 | 5.9 | 22.3 | 84.2 | 70.1 |
| 4 | 12 cm Heel | 19 | 168 | 48 | 17 | 37 | 115.1 | 1.77 | 96.5 | 1.13 | 1.07 | 1.1 | 1.84 | 2 | 1.92 | 111.6 | 121.3 | 116.45 | 63.1 | 60.3 | 61.7 | 36.9 | 39.7 | 38.3 | 10.2 | 9.3 | 9.75 | 43.1 | 40.3 | 41.7 | 10.2 | 11.2 | 10.7 | 86.6 | 86.9 | 75.9 |
| 5 | 12 cm Heel | 19 | 169 | 61 | 21.4 | 38 | 125.7 | 2.32 | 96.1 | 0.95 | 1 | 0.975 | 2.11 | 1.32 | 1.715 | 127.9 | 140.6 | 134.25 | 64.5 | 57.5 | 61 | 35.5 | 42.5 | 39 | 10.3 | 13 | 11.65 | 41.2 | 33.4 | 37.3 | 9.3 | 9.9 | 9.6 | 96.4 | 97.1 | 97.7 |
| 6 | 12 cm Heel | 19 | 172 | 59 | 19.9 | 39 | 101.3 | 1.79 | 92.1 | 1.4 | 1.5 | 1.45 | 2 | 1.3 | 1.65 | 120.9 | 139.4 | 130.15 | 52.7 | 63.1 | 57.9 | 47.3 | 36.9 | 42.1 | 8.4 | 10.2 | 9.3 | 35.3 | 44.6 | 39.95 | 9.9 | 8.8 | 9.35 | 90 | 95.7 | 36.7 |
| 7 | 12 cm Heel | 19 | 172 | 69 | 23.3 | 40 | 136.8 | 1.97 | 94.7 | 0.93 | 0.91 | 0.92 | 1.77 | 1.75 | 1.76 | 110.4 | 109.5 | 109.95 | 61.9 | 59.7 | 60.8 | 38.1 | 40.3 | 39.2 | 11.6 | 12.4 | 12 | 39.1 | 35.7 | 37.4 | 6.9 | 7.9 | 7.4 | 65.6 | 93.1 | 91 |
| 8 | 12 cm Heel | 19 | 173 | 67 | 22.4 | 38 | 125.2 | 1.76 | 94.7 | 1.01 | 1 | 1.005 | 1.78 | 1.71 | 1.745 | 111.4 | 106.8 | 109.1 | 61.7 | 53.8 | 57.75 | 38.3 | 46.2 | 42.25 | 7.2 | 9.7 | 8.45 | 45.6 | 37.4 | 41.5 | 8.5 | 9.8 | 9.15 | 79.2 | 96.6 | 78.5 |
| 9 | 12 cm Heel | 20 | 159 | 77 | 30.5 | 37 | 123.7 | 1.68 | 96.6 | 0.97 | 0.97 | 0.97 | 1.63 | 1.65 | 1.64 | 101.8 | 102.9 | 102.35 | 60.6 | 59.5 | 60.05 | 39.4 | 40.5 | 39.95 | 9.6 | 10.7 | 10.15 | 40.4 | 39.5 | 39.95 | 11.4 | 13.3 | 12.35 | 93.4 | 97.9 | 84 |
| 10 | 12 cm Heel | 20 | 163 | 61 | 23 | 39 | 117 | 1.41 | 73.4 | 1.14 | 1.18 | 1.16 | 1.67 | 1.7 | 1.685 | 102.8 | 105.2 | 104 | 63 | 58.1 | 60.55 | 37 | 41.9 | 39.45 | 13.7 | 8.4 | 11.05 | 40.3 | 36.6 | 38.45 | 5.8 | 7.4 | 6.6 | 21.7 | 88.1 | 81.5 |
| 11 | 12 cm Heel | 20 | 164 | 76 | 28.3 | 39 | 107.3 | 1.35 | 88 | 1.04 | 1.19 | 1.115 | 1.44 | 1.53 | 1.485 | 89.1 | 94.2 | 91.65 | 59.8 | 58.8 | 59.3 | 40.2 | 41.2 | 40.7 | 11.6 | 12.5 | 12.05 | 33.9 | 36.7 | 35.3 | 9.9 | 7.9 | 8.9 | 41.6 | 92.1 | 98.3 |
| 12 | 12 cm Heel | 20 | 167 | 58 | 20.8 | 37 | 124 | 1.44 | 71.6 | 1.01 | 1.16 | 1.085 | 1.38 | 1.79 | 1.585 | 85.4 | 110.4 | 97.9 | 68.2 | 58.9 | 63.55 | 31.8 | 41.1 | 36.45 | 16.2 | 7.8 | 12 | 43.2 | 37.3 | 40.25 | 6.6 | 7.8 | 7.2 | 23.3 | 88.8 | 91 |
| 13 | 12 cm Heel | 20 | 169 | 65 | 22.8 | 37 | 104.7 | 1.7 | 96.8 | 1.18 | 1.28 | 1.23 | 1.87 | 1.91 | 1.89 | 114 | 116.7 | 115.35 | 59 | 54.2 | 56.6 | 41 | 45.8 | 43.4 | 9.6 | 7.7 | 8.65 | 41.3 | 37.7 | 39.5 | 11.7 | 10.5 | 11.1 | 89.2 | 96.9 | 85.3 |
| 14 | 12 cm Heel | 21 | 158 | 57 | 22.8 | 36 | 65.9 | 1.05 | 87.4 | 1.68 | 1.2 | 1.44 | 1.95 | 2.06 | 2.005 | 119.1 | 125.4 | 122.25 | 44.4 | 54 | 49.2 | 55.6 | 46 | 50.8 | 5.6 | 4.6 | 5.1 | 32.6 | 44.8 | 38.7 | 15.2 | 7.3 | 11.25 | 62.3 | 80.6 | 71.2 |
| 15 | 12 cm Heel | 21 | 163 | 57 | 21.5 | 38 | 120.7 | 1.46 | 97 | 1 | 0.98 | 0.99 | 1.43 | 1.43 | 1.43 | 87.4 | 87.5 | 87.45 | 59.8 | 59.9 | 59.85 | 40.2 | 40.1 | 40.15 | 10.2 | 10.6 | 10.4 | 39.2 | 38.5 | 38.85 | 8.8 | 8.9 | 8.85 | 88 | 96.8 | 88.2 |
| 16 | 12 cm Heel | 21 | 165 | 60 | 22 | 37 | 105.1 | 1.56 | 82.7 | 1.16 | 1.15 | 1.155 | 1.75 | 1.84 | 1.795 | 105.4 | 110.7 | 108.05 | 62 | 54.5 | 58.25 | 38 | 45.5 | 41.75 | 10.9 | 7.5 | 9.2 | 45.4 | 35.7 | 40.55 | 4.8 | 8 | 6.4 | 65.9 | 93.9 | 92.5 |
| 17 | 12 cm Heel | 21 | 165 | 63 | 23.1 | 38 | 113.2 | 1.47 | 68.6 | 1.08 | 1.08 | 1.08 | 1.53 | 1.63 | 1.58 | 91.9 | 98.2 | 95.05 | 64 | 55.4 | 59.7 | 36 | 44.6 | 40.3 | 12.9 | 4.9 | 8.9 | 46.5 | 36.8 | 41.65 | 4.9 | 9.2 | 7.05 | 66.8 | 88.1 | 86 |
| 18 | 12 cm Heel | 21 | 172 | 73 | 24.7 | 40 | 124.7 | 2.71 | 88.8 | 0.88 | 0.97 | 0.925 | 1.27 | 1.25 | 1.26 | 90.7 | 81.4 | 86.05 | 50 | 59.4 | 54.7 | 50 | 40.6 | 45.3 | 6.4 | 1.2 | 3.8 | 40.2 | 51.8 | 46 | 2 | 0.1 | 1.05 | 13.7 | 81.5 | 79.7 |
| 19 | 12 cm Heel | 21 | 176 | 62 | 20 | 37 | 106.1 | 1.45 | 83.2 | 1.37 | 1.34 | 1.355 | 1.86 | 1.93 | 1.895 | 109.2 | 113.8 | 111.5 | 60.7 | 59.9 | 60.3 | 39.3 | 40.1 | 39.7 | 8.5 | 12.2 | 10.35 | 42.2 | 39 | 40.6 | 9.5 | 8.3 | 8.9 | 78.1 | 90.3 | 91.4 |
| 20 | 12 cm Heel | 21 | 177 | 65 | 20.7 | 39 | 96.3 | 1.22 | 92 | 1.18 | 1.26 | 1.22 | 1.47 | 1.51 | 1.49 | 86.3 | 88.6 | 87.45 | 63.5 | 61 | 62.25 | 36.5 | 39 | 37.75 | 13.3 | 16 | 14.65 | 33.8 | 33.4 | 33.6 | 13 | 10.3 | 11.65 | 70.2 | 98 | 95.6 |
| 21 | 12 cm Heel | 22 | 157 | 51 | 20.7 | 36 | 101.7 | 1.12 | 96.2 | 1.17 | 1.2 | 1.185 | 1.29 | 1.34 | 1.315 | 75.9 | 79.1 | 77.5 | 62.8 | 62.1 | 62.45 | 37.2 | 37.9 | 37.55 | 13.1 | 12.7 | 12.9 | 35.6 | 36.9 | 36.25 | 9.7 | 7.4 | 8.55 | 76.8 | 97.9 | 83.9 |
| 22 | 12 cm Heel | 22 | 162 | 52 | 19.8 | 38 | 125.7 | 1.61 | 91.9 | 0.98 | 0.96 | 0.97 | 1.56 | 1.56 | 1.56 | 92 | 92 | 92 | 64 | 63.5 | 63.75 | 36 | 36.5 | 36.25 | 13.7 | 11.5 | 12.6 | 38.7 | 37 | 37.85 | 9.1 | 15.4 | 12.25 | 76.8 | 98.4 | 97.2 |

| IDENTIFICATION | | DEMOGRAPHICS | | | | | GENERAL GAIT | | | Stride Duration (s) | | | Stride Length (m) | | | % Stride Length (% height) | | | Stance Duration (%) | | | Swing Duration (%) | | | Double Support (%) | | | Single Support (%) | | | Propulsion Index | | | Pelvis Symmetry (Symmetry Index) | | |
|---|---|---|---|---|---|---|---|---|---|---|---|---|---|---|---|---|---|---|---|---|---|---|---|---|---|---|---|---|---|---|---|---|---|---|---|---|
| ID | Condition | Age (yr) | Height (cm) | Weight (kg) | BMI (kg/m$^2$) | Shoe Size (EU) | Cadence (steps/min) | Speed (m/s) | Symmetry Index | Left | Right | Avg | Left | Right | Avg | Left | Right | Avg | Left | Right | Avg | Left | Right | Avg | First Left | First Right | Avg First | Left | Right | Avg | Left | Right | Avg | Tilt | Obliquity | Rotation |
| 23 | 12 cm Heel | 22 | 162 | 53 | 20.2 | 39 | 135.3 | 1.65 | 87 | 0.91 | 1 | 0.955 | 1.49 | 1.64 | 1.565 | 87.6 | 96.3 | 91.95 | 64.5 | 61.3 | 62.9 | 35.5 | 38.7 | 37.1 | 12.1 | 9.9 | 11 | 41.7 | 41.2 | 41.45 | 8.5 | 10 | 9.25 | 71.9 | 98.8 | 60.1 |
| 24 | 12 cm Heel | 22 | 162 | 56 | 21.3 | 37 | 130.1 | 1.73 | 92.5 | 0.94 | 0.93 | 0.935 | 1.58 | 1.6 | 1.59 | 92.8 | 94.1 | 93.45 | 62.1 | 63.3 | 62.7 | 37.9 | 36.7 | 37.3 | 13.8 | 11.6 | 12.7 | 36.6 | 37.1 | 36.85 | 8.8 | 9.9 | 9.35 | 88.4 | 98.5 | 48.5 |
| 25 | 12 cm Heel | 22 | 168 | 63 | 22.3 | 38 | 119.6 | 2.18 | 90.8 | 1.01 | 1.01 | 1.01 | 1.19 | 2.2 | 1.695 | 126.6 | 127 | 126.8 | 59.4 | 60.7 | 60.05 | 40.6 | 39.3 | 39.95 | 8.4 | 10.9 | 9.65 | 40.1 | 40.8 | 40.45 | 12.4 | 11.4 | 11.9 | 88.2 | 96.3 | 94.5 |
| 26 | 12 cm Heel | 22 | 168 | 63 | 22.3 | 39 | 119.4 | 1.81 | 95.3 | 0.99 | 1.03 | 1.01 | 1.79 | 1.85 | 1.82 | 103.5 | 107.1 | 105.3 | 61.3 | 60.1 | 60.7 | 38.7 | 39.9 | 39.3 | 11.5 | 11.9 | 11.7 | 37.2 | 33.7 | 35.45 | 7.8 | 9.4 | 8.6 | 88.6 | 96.5 | 95.8 |
| 27 | 12 cm Heel | 22 | 170 | 64 | 22.1 | 37 | 96 | 1.5 | 91.5 | 1.24 | 1.28 | 1.26 | 1.83 | 1.88 | 1.855 | 105.8 | 108.8 | 107.3 | 58.7 | 57.1 | 57.9 | 41.3 | 42.9 | 42.1 | 10.3 | 9.4 | 9.85 | 40.8 | 37.5 | 39.15 | 8.9 | 8.7 | 8.8 | 78.8 | 94.1 | 76.8 |
| 28 | 12 cm Heel | 22 | 173 | 59 | 19.7 | 37 | 118.5 | 1.8 | 90.5 | 1.2 | 1.18 | 1.19 | 1.89 | 1.97 | 1.93 | 108 | 112.6 | 110.3 | 51.7 | 66.9 | 59.3 | 48.3 | 33.1 | 40.7 | 9.5 | 5.3 | 7.4 | 36.7 | 49.7 | 43.2 | 10.7 | 7.2 | 8.95 | 62.4 | 92 | 58.9 |
| 29 | 12 cm Heel | 23 | 163 | 81 | 30.5 | 38 | 100.3 | 1.5 | 87.3 | 1.18 | 1.17 | 1.175 | 1.8 | 1.69 | 1.745 | 102.8 | 96.8 | 99.8 | 59.5 | 63.3 | 61.4 | 40.5 | 36.7 | 38.6 | 10.1 | 12 | 11.05 | 37.5 | 38.9 | 38.2 | 13.9 | 10.8 | 12.35 | 37.3 | 97 | 94 |
| 30 | 12 cm Heel | 23 | 164 | 48 | 17.8 | 38 | 107.5 | 1.58 | 92.6 | 1.13 | 1.12 | 1.125 | 1.79 | 1.75 | 1.77 | 102.6 | 100 | 101.3 | 59.1 | 61.2 | 60.15 | 40.9 | 38.8 | 39.85 | 10.2 | 8.9 | 9.55 | 39.5 | 42.4 | 40.95 | 13.6 | 9.7 | 11.65 | 57.4 | 98 | 94.7 |
| 31 | 12 cm Heel | 23 | 166 | 60 | 21.8 | 38 | 132.9 | 1.55 | 98.5 | 0.91 | 0.91 | 0.91 | 1.4 | 1.43 | 1.415 | 87.3 | 89.1 | 88.2 | 69.9 | 70.7 | 70.3 | 30.1 | 29.3 | 29.7 | 20.2 | 19.6 | 19.9 | 30 | 30.2 | 30.1 | 16.3 | 17.4 | 16.85 | 93.5 | 97.2 | 80.3 |
| 32 | 12 cm Heel | 23 | 167 | 50 | 17.9 | 37 | 129.4 | 1.52 | 94.7 | 0.95 | 0.91 | 0.93 | 1.42 | 1.4 | 1.41 | 88.8 | 87.8 | 88.3 | 65.3 | 70.5 | 67.9 | 34.7 | 29.5 | 32.1 | 17.4 | 17.1 | 17.25 | 30.1 | 35.8 | 32.95 | 17 | 14.7 | 15.85 | 53.2 | 97.2 | 89.5 |
| 33 | 12 cm Heel | 23 | 172 | 57 | 19.3 | 39 | 126.6 | 1.42 | 89.5 | 0.96 | 0.95 | 0.955 | 1.36 | 1.35 | 1.355 | 85 | 84.3 | 84.65 | 69.8 | 63.9 | 66.85 | 30.2 | 36.1 | 33.15 | 18 | 15.6 | 16.8 | 35.8 | 30 | 32.9 | 11.6 | 16 | 13.8 | 52 | 96.6 | 95.8 |
| 34 | 12 cm Heel | 23 | 175 | 53 | 17.3 | 38 | 120.4 | 1.16 | 78.5 | 1.04 | 1 | 1.02 | 1.19 | 1.16 | 1.175 | 68.1 | 66.5 | 67.3 | 63 | 67.8 | 65.4 | 37 | 32.2 | 34.6 | 10.6 | 19.2 | 14.9 | 33.2 | 37.8 | 35.5 | 10.7 | 9.2 | 9.95 | 71.6 | 93 | 94.8 |
| 35 | 12 cm Heel | 23 | 175 | 63 | 20.6 | 37 | 117.9 | 1.12 | 97.3 | 1.06 | 1.02 | 1.04 | 1.17 | 1.14 | 1.155 | 67.1 | 65.3 | 66.2 | 66.1 | 64.5 | 65.3 | 33.9 | 35.5 | 34.7 | 15.1 | 14.9 | 15 | 36 | 34.2 | 35.1 | 10.8 | 10.2 | 10.5 | 73.4 | 99.1 | 98.8 |
| 36 | 12 cm Heel | 23 | 175 | 68 | 22.2 | 39 | 114.2 | 1.12 | 87.8 | 1.04 | 1.05 | 1.045 | 1.18 | 1.16 | 1.17 | 67.5 | 66.4 | 66.95 | 64.9 | 68.6 | 66.75 | 35.1 | 31.4 | 33.25 | 13.5 | 19.7 | 16.6 | 31.4 | 34.9 | 33.15 | 12.8 | 9.6 | 11.2 | 71.5 | 94.2 | 97.1 |
| 37 | 12 cm Heel | 24 | 159 | 56 | 22.2 | 36 | 121.3 | 1.64 | 94.3 | 0.98 | 1 | 0.99 | 1.63 | 1.62 | 1.625 | 99.7 | 98.6 | 99.15 | 61.6 | 61.6 | 61.6 | 38.4 | 38.4 | 38.4 | 9.8 | 13.9 | 11.85 | 38.3 | 37.4 | 37.85 | 11.7 | 10.6 | 11.15 | 95.5 | 98.3 | 97.3 |
| 38 | 12 cm Heel | 24 | 163 | 61 | 23 | 37 | 119.8 | 1.6 | 97.1 | 1 | 1 | 1 | 1.59 | 1.6 | 1.595 | 97.1 | 97.6 | 97.35 | 60.4 | 60.3 | 60.35 | 39.6 | 39.7 | 39.65 | 9.5 | 11.6 | 10.55 | 39.4 | 39.2 | 39.3 | 10.9 | 10 | 10.45 | 95 | 97.9 | 98.9 |
| 39 | 12 cm Heel | 24 | 166 | 51 | 18.5 | 36 | 119.1 | 1.56 | 97 | 1.01 | 1.01 | 1.01 | 1.6 | 1.56 | 1.58 | 97.4 | 95.4 | 96.4 | 61.5 | 63.5 | 62.5 | 38.5 | 36.5 | 37.5 | 11.6 | 12.4 | 12 | 37.4 | 38.5 | 37.95 | 11.5 | 10.2 | 10.85 | 95.3 | 98.7 | 97.5 |
| 40 | 12 cm Heel | 24 | 167 | 46 | 16.5 | 36 | 118.8 | 1.36 | 97.7 | 1.01 | 1.01 | 1.01 | 1.37 | 1.37 | 1.37 | 80.5 | 80.3 | 80.4 | 68.1 | 67.3 | 67.7 | 31.9 | 32.7 | 32.3 | 16.1 | 18.5 | 17.3 | 32.6 | 32.1 | 32.35 | 12.2 | 11.3 | 11.75 | 91.5 | 99.2 | 89.6 |
| 41 | 12 cm Heel | 24 | 171 | 55 | 18.8 | 38 | 122.6 | 1.41 | 97.4 | 1.01 | 1 | 1.005 | 1.41 | 1.41 | 1.41 | 82.8 | 83.2 | 83 | 65.9 | 63.5 | 64.7 | 34.1 | 36.5 | 35.3 | 14.7 | 15 | 14.85 | 36.6 | 35.1 | 35.85 | 11.6 | 11.5 | 11.55 | 43.2 | 97.5 | 81.7 |
| 42 | 12 cm Heel | 24 | 172 | 61 | 20.6 | 38 | 119.2 | 1.44 | 91.6 | 1.02 | 1.02 | 1.02 | 1.46 | 1.47 | 1.465 | 85.9 | 86.3 | 86.1 | 63.6 | 60.3 | 61.95 | 36.4 | 39.7 | 38.05 | 14.4 | 11.2 | 12.8 | 37.9 | 36.5 | 37.2 | 13.1 | 11.8 | 12.45 | 19.5 | 96.6 | 91.2 |
| 43 | 12 cm Heel | 24 | 177 | 66 | 21.1 | 39 | 117.9 | 1.34 | 95.5 | 1.05 | 1.03 | 1.04 | 1.4 | 1.38 | 1.39 | 81.2 | 80.1 | 80.65 | 62.2 | 61.5 | 61.85 | 37.8 | 38.5 | 38.15 | 14.6 | 10.7 | 12.65 | 37.1 | 38.6 | 37.85 | 11.4 | 9.3 | 10.35 | 46 | 98.5 | 98.7 |
| 44 | 12 cm Heel | 25 | 162 | 54 | 20.6 | 37 | 116.4 | 1.4 | 88.3 | 1.04 | 1.02 | 1.03 | 1.45 | 1.45 | 1.45 | 83.6 | 83.7 | 83.65 | 56.4 | 60.3 | 58.35 | 43.6 | 39.7 | 41.65 | 8.2 | 8.8 | 8.5 | 39.8 | 43.6 | 41.7 | 13.6 | 10.2 | 11.9 | 72.5 | 98.6 | 98.7 |
| 45 | 12 cm Heel | 25 | 169 | 59 | 20.7 | 38 | 116.4 | 1.4 | 94.1 | 1.03 | 1.03 | 1.03 | 1.45 | 1.44 | 1.445 | 83.6 | 83.4 | 83.5 | 57.8 | 60.2 | 59 | 42.2 | 39.8 | 41 | 8.7 | 9.3 | 9 | 39.6 | 42.4 | 41 | 11.1 | 9 | 10.05 | 77.3 | 98.9 | 98.5 |
| 46 | 12 cm Heel | 25 | 172 | 54 | 18.3 | 36 | 125.5 | 1.44 | 94.8 | 0.96 | 0.94 | 0.95 | 1.36 | 1.38 | 1.37 | 82.7 | 84 | 83.35 | 61.9 | 60.5 | 61.2 | 38.1 | 39.5 | 38.8 | 11.2 | 11.4 | 11.3 | 38.9 | 37.7 | 38.3 | 12.4 | 12.1 | 12.25 | 38.1 | 72.8 | 97.4 |
| 47 | 12 cm Heel | 26 | 162 | 47 | 17.9 | 36 | 120.2 | 1.4 | 93 | 1.05 | 1.05 | 1.05 | 1.43 | 1.44 | 1.435 | 87.1 | 87.6 | 87.35 | 64 | 62.5 | 63.25 | 36 | 37.5 | 36.75 | 14.5 | 12.3 | 13.4 | 36.9 | 36.6 | 36.75 | 16.6 | 15.2 | 15.9 | 52.4 | 65.2 | 94.4 |
| 48 | 12 cm Heel | 26 | 164 | 50 | 18.6 | 37 | 121.6 | 1.5 | 94.3 | 0.99 | 0.98 | 0.985 | 1.48 | 1.48 | 1.48 | 90.5 | 90.2 | 90.35 | 61.9 | 59.3 | 60.6 | 38.1 | 40.7 | 39.4 | 10.1 | 11.4 | 10.75 | 40.7 | 37.7 | 39.2 | 14.3 | 13.6 | 13.95 | 54.7 | 68.9 | 96.9 |
| 49 | 12 cm Heel | 26 | 169 | 58 | 20.3 | 37 | 123.6 | 1.38 | 98.4 | 0.97 | 0.98 | 0.975 | 1.33 | 1.35 | 1.34 | 78 | 78.8 | 78.4 | 65.4 | 64.3 | 64.85 | 34.6 | 35.7 | 35.15 | 15.1 | 14.2 | 14.65 | 36.3 | 35 | 35.65 | 14.2 | 13.3 | 13.75 | 92.5 | 98.3 | 98.2 |
| 50 | 12 cm Heel | 26 | 172 | 55 | 18.6 | 37 | 114.5 | 1.2 | 97.3 | 1.04 | 1.05 | 1.045 | 1.26 | 1.26 | 1.26 | 74 | 73.8 | 73.9 | 64.9 | 63.5 | 64.2 | 35.1 | 36.5 | 35.8 | 15.7 | 15 | 15.35 | 34.4 | 34.7 | 34.55 | 13.5 | 10.6 | 12.05 | 97 | 98.1 | 97.4 |
| 51 | 12 cm Heel | 26 | 173 | 77 | 25.7 | 40 | 108 | 1.1 | 90.2 | 1.25 | 1.22 | 1.235 | 1.36 | 1.54 | 1.45 | 79.3 | 89.9 | 84.6 | 71.4 | 51.1 | 61.25 | 28.6 | 48.9 | 38.75 | 12.7 | 10.9 | 11.8 | 49.6 | 27.8 | 38.7 | 8.8 | 11.5 | 10.15 | 83.2 | 96.4 | 89 |
| 52 | 12 cm Heel | 27 | 158 | 61 | 24.4 | 37 | 120.5 | 1.54 | 55.4 | 1 | 1.01 | 1.005 | 1.54 | 1.56 | 1.55 | 93.4 | 94.5 | 93.95 | 58.9 | 69 | 63.95 | 41.1 | 31 | 36.05 | 8.5 | 20.1 | 14.3 | 31.2 | 39.9 | 35.55 | 19.3 | 10.5 | 14.9 | 35.4 | 79.6 | 87.1 |
| 53 | 12 cm Heel | 27 | 162 | 64 | 24.4 | 37 | 122.2 | 1.59 | 67.9 | 1.01 | 0.98 | 0.995 | 1.58 | 1.58 | 1.58 | 95.8 | 95.5 | 95.65 | 56.5 | 64.5 | 60.5 | 43.5 | 35.5 | 39.5 | 6.9 | 11.8 | 9.35 | 36.1 | 45.4 | 40.75 | 16.3 | 11.8 | 14.05 | 56.9 | 86.9 | 93.9 |
| 54 | 12 cm Heel | 27 | 163 | 51 | 19.2 | 36 | 118.3 | 1.52 | 85.9 | 1.03 | 1.03 | 1.03 | 1.55 | 1.55 | 1.55 | 94.2 | 93.9 | 94.05 | 56.9 | 61.5 | 59.2 | 43.1 | 38.5 | 40.8 | 7.3 | 11.1 | 9.2 | 39.1 | 43.2 | 41.15 | 15.5 | 13 | 14.25 | 44.2 | 93.8 | 97.3 |
| 55 | 12 cm Heel | 27 | 164 | 66 | 24.5 | 36 | 139.4 | 1.73 | 55.9 | 0.92 | 0.9 | 0.91 | 1.57 | 1.58 | 1.575 | 97.8 | 97.9 | 97.85 | 70.2 | 63.9 | 67.05 | 29.8 | 36.1 | 32.95 | 22.9 | 9.2 | 16.05 | 38.2 | 30.3 | 34.25 | 11.4 | 13.8 | 12.6 | 38.3 | 88.1 | 85.1 |
| 56 | 12 cm Heel | 27 | 172 | 57 | 19.3 | 39 | 142.8 | 1.78 | 52.3 | 0.91 | 0.89 | 0.9 | 1.59 | 1.6 | 1.595 | 98.6 | 99.2 | 98.9 | 73.2 | 62.4 | 67.8 | 26.8 | 37.6 | 32.2 | 23.1 | 11.7 | 17.4 | 38.7 | 27.4 | 33.05 | 10.3 | 16.4 | 13.35 | 36.4 | 85.8 | 80.6 |
| 57 | 12 cm Heel | 28 | 162 | 60 | 22.9 | 36 | 140.8 | 1.57 | 81.7 | 1.03 | 0.9 | 0.965 | 1.35 | 1.53 | 1.44 | 93.3 | 94.8 | 94.05 | 78.8 | 66.6 | 72.7 | 21.2 | 33.4 | 27.3 | 16.4 | 17.3 | 16.85 | 48.9 | 26.5 | 37.7 | 9.3 | 19.1 | 14.2 | 38.2 | 95.8 | 89.8 |
| 58 | 12 cm Heel | 28 | 163 | 52 | 15.8 | 37 | 147.6 | 2.04 | 95.5 | 0.93 | 0.87 | 0.9 | 1.75 | 1.66 | 1.705 | 107.5 | 101.7 | 104.6 | 59.5 | 69.6 | 64.55 | 40.5 | 30.4 | 35.45 | 12.9 | 12.9 | 12.9 | 36.3 | 42.3 | 39.3 | 10.5 | 7.8 | 9.15 | 36 | 94.7 | 97.3 |
| 59 | 12 cm Heel | 28 | 168 | 55 | 19.5 | 36 | 114.1 | 1.21 | 94.7 | 1.02 | 1.1 | 1.06 | 1.31 | 1.23 | 1.27 | 80.5 | 75.6 | 78.05 | 67.1 | 64 | 65.55 | 32.9 | 36 | 34.45 | 15.9 | 15.9 | 15.9 | 33.7 | 33.1 | 33.4 | 10.4 | 11.2 | 10.8 | 82 | 96.3 | 97 |
| 60 | 12 cm Heel | 28 | 172 | 65 | 22 | 38 | 145.7 | 1.83 | 94.9 | 0.86 | 0.88 | 0.87 | 1.45 | 1.53 | 1.49 | 89.1 | 93.7 | 91.4 | 62.8 | 64.7 | 63.75 | 37.2 | 35.3 | 36.25 | 13.9 | 13 | 13.45 | 36.2 | 38 | 37.1 | 9.4 | 9.5 | 9.45 | 88.2 | 91 | 93.9 |
| 61 | 12 cm Heel | 28 | 176 | 66 | 21.3 | 37 | 101.1 | 1.59 | 95.1 | 1.19 | 1.16 | 1.175 | 1.76 | 1.93 | 1.845 | 100.5 | 110.5 | 105.5 | 59.4 | 59.3 | 59.35 | 40.6 | 40.7 | 40.65 | 12.2 | 8 | 10.1 | 39.6 | 38.4 | 39 | 12.1 | 11.5 | 11.8 | 41.8 | 98.5 | 95 |
| 62 | 12 cm Heel | 28 | 177 | 81 | 25.9 | 39 | 106.7 | 1.25 | 92.8 | 1.14 | 1.16 | 1.15 | 1.5 | 1.39 | 1.445 | 85.5 | 79.3 | 82.4 | 57.7 | 58.5 | 58.1 | 42.3 | 41.5 | 41.9 | 6.9 | 7.6 | 7.25 | 43.3 | 44 | 43.65 | 9.2 | 8.6 | 8.9 | 38.8 | 98.4 | 94.6 |
| 63 | 12 cm Heel | 29 | 162 | 45 | 17.1 | 36 | 102.8 | 1.25 | 88.3 | 1.18 | 1.19 | 1.185 | 1.53 | 1.41 | 1.47 | 87.4 | 80.8 | 84.1 | 60.9 | 56.2 | 58.55 | 39.1 | 43.8 | 41.45 | 8.9 | 8.1 | 8.5 | 44 | 39.2 | 41.6 | 9 | 9.5 | 9.25 | 34.5 | 97.6 | 95.2 |
| 64 | 12 cm Heel | 29 | 162 | 46 | 17.5 | 36 | 122.9 | 1.76 | 75.3 | 1.1 | 1.05 | 1.075 | 1.99 | 1.83 | 1.91 | 115.2 | 105.7 | 110.45 | 64.2 | 53.8 | 59 | 35.8 | 46.2 | 41 | 11.8 | 5.2 | 8.5 | 45.4 | 36.2 | 40.8 | 5.8 | 6.2 | 6 | 53.7 | 84.6 | 93.3 |
| 65 | 12 cm Heel | 29 | 165 | 59 | 21.7 | 36 | 102.3 | 1.67 | 92.7 | 1.19 | 1.22 | 1.205 | 2.09 | 1.9 | 1.995 | 120.5 | 109.9 | 115.2 | 62.4 | 62.3 | 62.35 | 37.6 | 37.7 | 37.65 | 13 | 11.5 | 12.25 | 39.3 | 37.5 | 38.4 | 7.2 | 5.9 | 6.55 | 65.4 | 98.1 | 99 |
| 66 | 12 cm Heel | 29 | 165 | 65 | 23.9 | 38 | 102.9 | 1.73 | 94.5 | 1.19 | 1.21 | 1.2 | 2.1 | 1.92 | 2.01 | 121.6 | 111 | 116.3 | 60.2 | 60.9 | 60.55 | 39.8 | 39.1 | 39.45 | 11.1 | 9 | 10.05 | 40.4 | 40.2 | 40.3 | 7.3 | 5.9 | 6.6 | 61.3 | 98.6 | 99 |
| 67 | 12 cm Heel | 29 | 180 | 59 | 18.2 | 40 | 100 | 1.03 | 98.1 | 1.2 | 1.2 | 1.2 | 1.24 | 1.23 | 1.235 | 76.3 | 75.6 | 75.95 | 61.5 | 62.4 | 61.95 | 38.5 | 37.6 | 38.05 | 12.5 | 11.8 | 12.15 | 37.3 | 38.4 | 37.85 | 7.9 | 8.6 | 8.25 | 92.5 | 97.3 | 98.8 |
| 68 | 12 cm Heel | 29 | 183 | 64 | 19.1 | 40 | 101.7 | 1.06 | 97.4 | 1.18 | 1.19 | 1.185 | 1.25 | 1.26 | 1.255 | 76.9 | 77.4 | 77.15 | 61.8 | 63.1 | 62.45 | 38.2 | 36.9 | 37.55 | 11.5 | 13.1 | 12.3 | 37.2 | 38.6 | 37.9 | 8.2 | 8.3 | 8.25 | 89.6 | 96.5 | 96.2 |
| 69 | 12 cm Heel | 30 | 161 | 62 | 23.9 | 36 | 105.7 | 1.1 | 98.4 | 1.13 | 1.15 | 1.14 | 1.24 | 1.25 | 1.245 | 76.3 | 76.8 | 76.55 | 64.7 | 63.5 | 64.1 | 35.3 | 36.5 | 35.9 | 13.7 | 13.9 | 13.8 | 37.2 | 35.4 | 36.3 | 7.9 | 9.9 | 8.9 | 87.2 | 98.4 | 97.1 |
| 70 | 12 cm Heel | 30 | 164 | 63 | 23.4 | 38 | 119.6 | 2.25 | 84.4 | 1.03 | 0.97 | 1 | 2.05 | 1.38 | 1.715 | 123.2 | 143.3 | 133.25 | 66.3 | 62.2 | 64.25 | 33.7 | 37.8 | 35.75 | 17.6 | 9.9 | 13.75 | 35.6 | 33.7 | 34.65 | 9.8 | 11.5 | 10.65 | 40.2 | 94.9 | 96.8 |
| 71 | 12 cm Heel | 30 | 175 | 82 | 26.8 | 40 | 121.7 | 2.25 | 95.9 | 1.01 | 1.02 | 1.015 | 1.34 | 2.16 | 1.75 | 140.7 | 130 | 135.35 | 61.3 | 61.2 | 61.25 | 38.7 | 38.8 | 38.75 | 10 | 11.3 | 10.65 | 39.9 | 40.9 | 40.4 | 6.8 | 6.7 | 6.75 | 49.3 | 99.1 | 99.3 |
| 72 | 12 cm Heel | 31 | 169 | 61 | 21.4 | 37 | 117.9 | 1.9 | 90.8 | 1.04 | 1.03 | 1.035 | 1.92 | 1.97 | 1.945 | 115.4 | 118.8 | 117.1 | 61.5 | 62.4 | 61.95 | 38.5 | 37.6 | 38.05 | 11.7 | 11.3 | 11.5 | 38.4 | 39.3 | 38.85 | 9.3 | 6.5 | 7.9 | 63.7 | 98.5 | 98.7 |

| IDENTIFICATION | | DEMOGRAPHICS | | | | | GENERAL GAIT | | | Stride Duration (s) | | | Stride Length (m) | | | % Stride Length (% height) | | | Stance Duration (%) | | | Swing Duration (%) | | | Double Support (%) | | | Single Support (%) | | | Propulsion Index | | | Pelvis Symmetry (Symmetry Index) | | |
|---|---|---|---|---|---|---|---|---|---|---|---|---|---|---|---|---|---|---|---|---|---|---|---|---|---|---|---|---|---|---|---|---|---|---|---|---|---|---|
| ID | Condition | Age (yr) | Height (cm) | Weight (kg) | BMI (kg/m$^2$) | Shoe Size (EU) | Cadence (steps/min) | Speed (m/s) | Symmetry Index | Left | Right | Avg | Left | Right | Avg | Left | Right | Avg | Left | Right | Avg | Left | Right | Avg | First Left | First Right | Avg First | Left | Right | Avg | Left | Right | Avg | Tilt | Obliquity | Rotation |
| 73 | 12 cm Heel | 31 | 171 | 70 | 23.9 | 39 | 113 | 1.27 | 97.1 | 1.06 | 1.07 | 1.065 | 1.37 | 1.35 | 1.36 | 75.9 | 75.1 | 75.5 | 59.4 | 56.8 | 58.1 | 40.6 | 43.2 | 41.9 | 7.2 | 7.9 | 7.55 | 43.7 | 41.6 | 42.65 | 7.1 | 7.6 | 7.35 | 54.5 | 95.2 | 89.7 |
| 74 | 12 cm Heel | 32 | 165 | 50 | 18.4 | 37 | 111.7 | 1.21 | 94.7 | 1.07 | 1.08 | 1.075 | 1.31 | 1.3 | 1.305 | 72.8 | 72.4 | 72.6 | 61.2 | 56.6 | 58.9 | 38.8 | 43.4 | 41.1 | 6.8 | 11.1 | 8.95 | 43.8 | 38.8 | 41.3 | 7.6 | 9.5 | 8.55 | 36.4 | 94.9 | 99.1 |
| 75 | 12 cm Heel | 32 | 177 | 60 | 19.2 | 39 | 111.2 | 1.2 | 97.7 | 1.09 | 1.08 | 1.085 | 1.3 | 1.3 | 1.3 | 72.2 | 72 | 72.1 | 62.2 | 63.2 | 62.7 | 37.8 | 36.8 | 37.3 | 11.8 | 12.7 | 12.25 | 37.8 | 38.2 | 38 | 6.7 | 7.7 | 7.2 | 60.4 | 91.8 | 79.8 |

*Abbreviations: BMI = body-mass index; EU = European shoe sizing. Values are reported as recorded by the instrumented gait analysis; no values were missing.*
