# Supplementary material for: Footwear Heel Height and Gait Biomechanics in Healthy Young Women: A Within-Subject Analysis of Spatiotemporal Parameters, Propulsion, and Pelvic Kinematics
Source: Life (Basel). 2026 Jun 10;16(6):977. doi: 10.3390/life16060977 (PMC13300841; doi:10.3390/life16060977)
Supplement: Supplementary file 1 [file life-16-00977-s001.zip › Table_S2_Effect_sizes.pdf]

Table S2. Effect sizes for post-hoc pairwise comparisons (Wilcoxon signed-rank)

Matched-pairs rank-biserial correlation (r) for each pairwise footwear comparison; |r| ~ 0.1 / 0.3 / 0.5 denote small / medium / large effects. p-adj = Bonferroni-corrected p-value (x6), significant at p-adj < 0.05. N = 75; df = 74. Significant rows are shaded.

Gait parameter	Comparison	Rank-biserial r	Adjusted p (p-adj)	Significant
Cadence (steps/min)	Barefoot vs Ballerina	-0.027	1.000	No
	Barefoot vs 8 cm Heel	-0.099	1.000	No
	Barefoot vs 12 cm Heel	-0.068	1.000	No
	Ballerina vs 8 cm Heel	-0.086	1.000	No
	Ballerina vs 12 cm Heel	-0.034	1.000	No
	8 cm Heel vs 12 cm Heel	0.020	1.000	No
Speed (m/s)	Barefoot vs Ballerina	0.330	0.082	No
	Barefoot vs 8 cm Heel	0.414	0.011	Yes
	Barefoot vs 12 cm Heel	0.348	0.052	No
	Ballerina vs 8 cm Heel	0.364	0.037	Yes
	Ballerina vs 12 cm Heel	0.087	1.000	No
	8 cm Heel vs 12 cm Heel	-0.151	1.000	No
Symmetry Index	Barefoot vs Ballerina	0.093	1.000	No
	Barefoot vs 8 cm Heel	-0.254	0.358	No
	Barefoot vs 12 cm Heel	-0.383	0.025	Yes
	Ballerina vs 8 cm Heel	-0.246	0.387	No
	Ballerina vs 12 cm Heel	-0.342	0.060	No
	8 cm Heel vs 12 cm Heel	-0.161	1.000	No
Left Stride Duration (s)	Barefoot vs Ballerina	-0.126	1.000	No
	Barefoot vs 8 cm Heel	0.006	1.000	No
	Barefoot vs 12 cm Heel	0.027	1.000	No
	Ballerina vs 8 cm Heel	0.290	0.213	No
	Ballerina vs 12 cm Heel	0.168	1.000	No
	8 cm Heel vs 12 cm Heel	0.008	1.000	No
Right Stride Duration (s)	Barefoot vs Ballerina	-0.023	1.000	No
	Barefoot vs 8 cm Heel	0.083	1.000	No
	Barefoot vs 12 cm Heel	0.150	1.000	No
	Ballerina vs 8 cm Heel	0.176	1.000	No
	Ballerina vs 12 cm Heel	0.121	1.000	No
	8 cm Heel vs 12 cm Heel	0.067	1.000	No
Avg Stride Duration (s)	Barefoot vs Ballerina	-0.069	1.000	No
	Barefoot vs 8 cm Heel	0.057	1.000	No
	Barefoot vs 12 cm Heel	0.101	1.000	No
	Ballerina vs 8 cm Heel	0.233	0.491	No
	Ballerina vs 12 cm Heel	0.133	1.000	No
	8 cm Heel vs 12 cm Heel	0.036	1.000	No
Left Stride Length (m)	Barefoot vs Ballerina	0.265	0.286	No
	Barefoot vs 8 cm Heel	0.333	0.073	No
	Barefoot vs 12 cm Heel	0.242	0.413	No
	Ballerina vs 8 cm Heel	0.421	0.009	Yes
	Ballerina vs 12 cm Heel	0.138	1.000	No
	8 cm Heel vs 12 cm Heel	-0.286	0.194	No
Right Stride Length (m)	Barefoot vs Ballerina	0.375	0.029	Yes
	Barefoot vs 8 cm Heel	0.427	0.008	Yes
	Barefoot vs 12 cm Heel	0.423	0.009	Yes
	Ballerina vs 8 cm Heel	0.392	0.022	Yes
	Ballerina vs 12 cm Heel	0.162	1.000	No
	8 cm Heel vs 12 cm Heel	-0.148	1.000	No

Gait parameter	Comparison	Rank-biserial r	Adjusted p (p-adj)	Significant
Avg Stride Length (m)	Barefoot vs Ballerina	0.327	0.083	No
	Barefoot vs 8 cm Heel	0.362	0.039	Yes
	Barefoot vs 12 cm Heel	0.339	0.065	No
	Ballerina vs 8 cm Heel	0.435	0.006	Yes
	Ballerina vs 12 cm Heel	0.193	0.883	No
	8 cm Heel vs 12 cm Heel	-0.234	0.497	No
% Left Stride Length (% height)	Barefoot vs Ballerina	0.296	0.155	No
	Barefoot vs 8 cm Heel	0.363	0.038	Yes
	Barefoot vs 12 cm Heel	0.355	0.045	Yes
	Ballerina vs 8 cm Heel	0.459	0.003	Yes
	Ballerina vs 12 cm Heel	0.234	0.485	No
	8 cm Heel vs 12 cm Heel	-0.234	0.469	No
% Right Stride Length (% height)	Barefoot vs Ballerina	0.382	0.024	Yes
	Barefoot vs 8 cm Heel	0.447	0.005	Yes
	Barefoot vs 12 cm Heel	0.468	0.003	Yes
	Ballerina vs 8 cm Heel	0.428	0.008	Yes
	Ballerina vs 12 cm Heel	0.249	0.376	No
	8 cm Heel vs 12 cm Heel	-0.063	1.000	No
Avg % Stride Length (% height)	Barefoot vs Ballerina	0.341	0.062	No
	Barefoot vs 8 cm Heel	0.392	0.019	Yes
	Barefoot vs 12 cm Heel	0.411	0.012	Yes
	Ballerina vs 8 cm Heel	0.475	0.002	Yes
	Ballerina vs 12 cm Heel	0.247	0.376	No
	8 cm Heel vs 12 cm Heel	-0.116	1.000	No
Left Stance Duration (%)	Barefoot vs Ballerina	-0.446	0.005	Yes
	Barefoot vs 8 cm Heel	-0.065	1.000	No
	Barefoot vs 12 cm Heel	0.035	1.000	No
	Ballerina vs 8 cm Heel	0.288	0.182	No
	Ballerina vs 12 cm Heel	0.356	0.046	Yes
	8 cm Heel vs 12 cm Heel	0.041	1.000	No
Right Stance Duration (%)	Barefoot vs Ballerina	-0.341	0.061	No
	Barefoot vs 8 cm Heel	-0.074	1.000	No
	Barefoot vs 12 cm Heel	0.044	1.000	No
	Ballerina vs 8 cm Heel	0.237	0.448	No
	Ballerina vs 12 cm Heel	0.328	0.086	No
	8 cm Heel vs 12 cm Heel	0.160	1.000	No
Avg Stance Duration (%)	Barefoot vs Ballerina	-0.583	<0.001	Yes
	Barefoot vs 8 cm Heel	-0.155	1.000	No
	Barefoot vs 12 cm Heel	0.035	1.000	No
	Ballerina vs 8 cm Heel	0.279	0.223	No
	Ballerina vs 12 cm Heel	0.382	0.024	Yes
	8 cm Heel vs 12 cm Heel	0.152	1.000	No
Left Swing Duration (%)	Barefoot vs Ballerina	0.447	0.005	Yes
	Barefoot vs 8 cm Heel	0.065	1.000	No
	Barefoot vs 12 cm Heel	-0.032	1.000	No
	Ballerina vs 8 cm Heel	-0.288	0.181	No
	Ballerina vs 12 cm Heel	-0.356	0.046	Yes
	8 cm Heel vs 12 cm Heel	-0.040	1.000	No
Right Swing Duration (%)	Barefoot vs Ballerina	0.341	0.062	No
	Barefoot vs 8 cm Heel	0.074	1.000	No
	Barefoot vs 12 cm Heel	-0.044	1.000	No
	Ballerina vs 8 cm Heel	-0.236	0.451	No

Gait parameter	Comparison	Rank-biserial r	Adjusted p (p-adj)	Significant
	Ballerina vs 12 cm Heel	-0.328	0.086	No
	8 cm Heel vs 12 cm Heel	-0.160	1.000	No
Avg Swing Duration (%)	Barefoot vs Ballerina	0.583	<0.001	Yes
	Barefoot vs 8 cm Heel	0.154	1.000	No
	Barefoot vs 12 cm Heel	-0.035	1.000	No
	Ballerina vs 8 cm Heel	-0.279	0.223	No
	Ballerina vs 12 cm Heel	-0.382	0.024	Yes
	8 cm Heel vs 12 cm Heel	-0.152	1.000	No
First Left Double Support (%)	Barefoot vs Ballerina	-0.551	<0.001	Yes
	Barefoot vs 8 cm Heel	-0.059	1.000	No
	Barefoot vs 12 cm Heel	0.174	1.000	No
	Ballerina vs 8 cm Heel	0.303	0.141	No
	Ballerina vs 12 cm Heel	0.522	<0.001	Yes
	8 cm Heel vs 12 cm Heel	0.096	1.000	No
First Right Double Support (%)	Barefoot vs Ballerina	-0.484	0.002	Yes
	Barefoot vs 8 cm Heel	-0.195	0.885	No
	Barefoot vs 12 cm Heel	-0.141	1.000	No
	Ballerina vs 8 cm Heel	0.139	1.000	No
	Ballerina vs 12 cm Heel	0.189	0.924	No
	8 cm Heel vs 12 cm Heel	0.066	1.000	No
Avg First Double Support (%)	Barefoot vs Ballerina	-0.588	<0.001	Yes
	Barefoot vs 8 cm Heel	-0.116	1.000	No
	Barefoot vs 12 cm Heel	0.023	1.000	No
	Ballerina vs 8 cm Heel	0.277	0.228	No
	Ballerina vs 12 cm Heel	0.379	0.026	Yes
	8 cm Heel vs 12 cm Heel	0.110	1.000	No
Left Single Support (%)	Barefoot vs Ballerina	0.358	0.042	Yes
	Barefoot vs 8 cm Heel	0.033	1.000	No
	Barefoot vs 12 cm Heel	-0.058	1.000	No
	Ballerina vs 8 cm Heel	-0.261	0.299	No
	Ballerina vs 12 cm Heel	-0.325	0.086	No
	8 cm Heel vs 12 cm Heel	-0.179	1.000	No
Right Single Support (%)	Barefoot vs Ballerina	0.444	0.005	Yes
	Barefoot vs 8 cm Heel	0.072	1.000	No
	Barefoot vs 12 cm Heel	-0.055	1.000	No
	Ballerina vs 8 cm Heel	-0.273	0.238	No
	Ballerina vs 12 cm Heel	-0.362	0.041	Yes
	8 cm Heel vs 12 cm Heel	-0.043	1.000	No
Avg Single Support (%)	Barefoot vs Ballerina	0.581	<0.001	Yes
	Barefoot vs 8 cm Heel	0.094	1.000	No
	Barefoot vs 12 cm Heel	-0.013	1.000	No
	Ballerina vs 8 cm Heel	-0.294	0.160	No
	Ballerina vs 12 cm Heel	-0.400	0.017	Yes
	8 cm Heel vs 12 cm Heel	-0.067	1.000	No
Left Propulsion Index	Barefoot vs Ballerina	0.190	0.934	No
	Barefoot vs 8 cm Heel	0.342	0.067	No
	Barefoot vs 12 cm Heel	0.641	<0.001	Yes
	Ballerina vs 8 cm Heel	0.267	0.277	No
	Ballerina vs 12 cm Heel	0.620	<0.001	Yes
	8 cm Heel vs 12 cm Heel	0.409	0.013	Yes
Right Propulsion Index	Barefoot vs Ballerina	0.206	0.758	No
	Barefoot vs 8 cm Heel	0.388	0.021	Yes

Gait parameter	Comparison	Rank-biserial r	Adjusted p (p-adj)	Significant
	Barefoot vs 12 cm Heel	0.612	<0.001	Yes
	Ballerina vs 8 cm Heel	0.395	0.026	Yes
	Ballerina vs 12 cm Heel	0.528	<0.001	Yes
	8 cm Heel vs 12 cm Heel	0.239	0.442	No
Avg Propulsion Index	Barefoot vs Ballerina	0.200	0.805	No
	Barefoot vs 8 cm Heel	0.419	0.011	Yes
	Barefoot vs 12 cm Heel	0.660	<0.001	Yes
	Ballerina vs 8 cm Heel	0.437	0.006	Yes
	Ballerina vs 12 cm Heel	0.691	<0.001	Yes
	8 cm Heel vs 12 cm Heel	0.414	0.011	Yes
TILT - Symmetry Index	Barefoot vs Ballerina	0.027	1.000	No
	Barefoot vs 8 cm Heel	-0.186	0.970	No
	Barefoot vs 12 cm Heel	-0.071	1.000	No
	Ballerina vs 8 cm Heel	-0.210	0.702	No
	Ballerina vs 12 cm Heel	-0.090	1.000	No
	8 cm Heel vs 12 cm Heel	0.085	1.000	No
OBLIQUITY - Symmetry Index	Barefoot vs Ballerina	0.157	1.000	No
	Barefoot vs 8 cm Heel	-0.128	1.000	No
	Barefoot vs 12 cm Heel	-0.270	0.271	No
	Ballerina vs 8 cm Heel	-0.384	0.025	Yes
	Ballerina vs 12 cm Heel	-0.431	0.007	Yes
	8 cm Heel vs 12 cm Heel	-0.247	0.381	No
ROTATION - Symmetry Index	Barefoot vs Ballerina	-0.126	1.000	No
	Barefoot vs 8 cm Heel	-0.082	1.000	No
	Barefoot vs 12 cm Heel	-0.237	0.448	No
	Ballerina vs 8 cm Heel	-0.022	1.000	No
	Ballerina vs 12 cm Heel	-0.250	0.359	No
	8 cm Heel vs 12 cm Heel	-0.314	0.108	No
